# Correlates of cardiorespiratory fitness in a population-based sample of middle-aged adults: cross-sectional analyses in the SCAPIS study

**DOI:** 10.1136/bmjopen-2022-066336

**Published:** 2022-12-14

**Authors:** Mats Börjesson, Örjan Ekblom, Daniel Arvidsson, Emerald G Heiland, Daniel Väisänen, Göran Bergström, Elin Ekblom-Bak

**Affiliations:** 1 Center for Health and Performance, Department of Molecular and Clinical Medicine, University of Gothenburg, Gothenburg, Sweden; 2 Department of Medicine, Geraiatric and Acute Medicine Östra, Sahlgrenska University Hospital, Goteborg, Sweden; 3 Department of Physical Activity and Health, Swedish School of Sport and Health Sciences GIH, Stockholm, Sweden; 4 Center for Health and Performance, Department of Food and Nutrition, and Sport Science, University of Gothenburg, Gothenburg, Sweden; 5 Department of Surgical Sciences, Medical Epidemiology, Uppsala University, Uppsala, Sweden; 6 Sahlgrenska Center for Cardiovascular and Metabolic Research, Wallenberg Laboratory, Sahlgrenska University Hospital, Gothenburg, Sweden; 7 Department of Molecular and Clinical Medicine, University of Gothenburg, Gothenburg, Sweden

**Keywords:** EPIDEMIOLOGY, PREVENTIVE MEDICINE, PUBLIC HEALTH

## Abstract

**Objectives:**

This study aimed to identify main sex-specific correlates of cardiorespiratory fitness (CRF) in a population-based, urban sample of Swedish adults.

**Design:**

Cross-sectional.

**Setting:**

Multi-site study at university hospitals, data from the Gothenburg site.

**Participants:**

A total of 5308 participants (51% women, aged 50–64 years) with a valid estimated VO_2_max, from submaximal cycle test, in the Swedish CArdioPulmonary bioImage Study (SCAPIS), were included.

**Primary and secondary outcomes:**

A wide range of correlates were examined including (a) sociodemographic and lifestyle behaviours, (b) perceived health, anthropometrics and chronic conditions and (c) self-reported as well as accelerometer-derived physical activity and sedentary behaviours. Both continuous levels of estimated VO_2_max as well as odds ratios (OR) and confidence intervals (CI)s of low VO_2_max (lowest sex-specific tertile) were reported.

**Results:**

In multivariable regression analyses, higher age, being born abroad, short education, high waist circumference, poor perceived health, high accelerometer-derived time in sedentary and low in vigorous physical activity, as well as being passive commuter, correlated independently and significantly with low VO_2_max in both men and women (OR range 1.31–9.58). Additionally in men, financial strain and being an ex-smoker are associated with higher odds for low VO_2_max (OR 2.15; 95% CI 1.33 to 3.48 and OR 1.40; 95% CI 1.09 to 1.80), while constant stress with lower odds (OR 0.61; 95% CI 0.43 to 0.85). Additionally in women, being a regular smoker is associated with lower odds for low VO_2_max (OR 0.64; 95% CI 0.45 to 0.92).

**Conclusions:**

The present study provides important reference material on CRF and correlates of CRF in a general middle-aged population, which can be valuable for future research, clinical practice and public health work. If relations are causal, increased knowledge about specific subgroups will aid in the development of appropriate, targeted interventions.

**Strengths and limitations of this study**

- The large population-based sample with estimated VO_2_max from a submaximal test and accelerometer-measured physical activity provides the power required to analyse multiple relevant correlates of cardiorespiratory fitness.
- Several high-risk subgroups with significantly lower estimated VO_2_max were identified.
- However, the cross-sectional design of the study prevents any causal inference, and we have no information on any genetic contribution to variation between subgroups of correlates.
- Although based on a large sample from the general population, participants included were younger, leaner, more physically active and had more often a university degree compared with excluded participants.

## Background

Levels of cardiorespiratory fitness (CRF), mainly determined by recent levels of moderate-to-vigorous physical activity and genetics,^1^ is important for aerobic performance and engagement in daily exercise activities. It is also a strong predictor of several non-communicable diseases and all-cause mortality.^2–4^ Unfortunately, data indicate a decline in CRF on population levels over the last decades.^5 6^ CRF is typically lower in older ages,^7^ in individuals with shorter education,^8^ daily smokers and obese persons, yet higher in those engaging regularly in leisure time physical activity (PA) or exercise.^9^ However, a broader knowledge of key determinants for CRF level is scarce, especially from population-based samples. For example, whether and how CRF levels vary by psychosocial factors and types of domain-specific PA behaviour are not fully elucidated or show conflicting results.^9 10^ Moreover, previous studies of PA correlates on CRF are often limited by the use of self-reported PA.^11^ Also, it is still not fully explored whether and how the age-related lower levels of CRF are associated with less exercise performed, or instead is due to age-related changes in key physiological capacities itself.^12^ Previous studies have mainly been based on selected samples, consisting of smaller cohorts (n<2000), which limits the power for comparisons between different subgroups.^9^ More detailed knowledge of current correlates in unselected samples is needed to understand variations of CRF in the population, not only for the development of targeted assessments of CRF in the population but also the generation of appropriate interventions.

The aim of the present study is to identify main sex-specific correlates of CRF in a population-based, urban sample of 50–64 year-old men and women, including a wide range of correlates: (a) sociodemographic factors and lifestyle behaviours, (b) perceived health, anthropometrics and chronic conditions and (c) self-reported as well as accelerometer-derived PA and sedentary behaviours. A secondary aim is to study whether the commonly reported lower CRF with older age might be influenced by level of accelerometer-derived sedentary time and/or vigorous PA.

## Methods

Data were retrieved from the Swedish CArdioPulmonary bioImage Study (SCAPIS).^13^ In 2012, a comprehensive pilot study was conducted and in 2013–2018 the full SCAPIS study was carried out in collaboration with six Swedish university hospitals (Gothenburg, Linköping, Malmö/Lund, Stockholm, Umeå and Uppsala), aiming to recruit 5000 individuals (men and women, 50–64 years) from their respective municipality. Data collection took place over two to three occasions within a 2-week period. The participants underwent extensive imaging and functional studies of the heart, lungs and metabolism, filled in an extensive questionnaire, and wore an accelerometer during 7 days. At each test-site, there was a possibility to include site-specific measurements. At the Gothenburg site, a submaximal cycle test was performed to estimate VO_2_max. In the pilot study, a total of 2243 participants were recruited from the census register, and 1111 (49.5%) agreed to participate. In the full study in Gothenburg, 12 109 were recruited and 6266 (52%) participated. Thus, a total of 7377 were eligible and 5308 participants (72%) provided valid estimated VO_2_max.

### Assessment of cardiorespiratory fitness

CRF was assessed as estimated VO_2_max using the Ekblom-Bak submaximal cycle ergometer test.^14^ The test uses the difference in heart rate response between 4 min cycling on a lower, standardised work rate (30 watts) and 4 min cycling on a higher, individually chosen work rate (aiming at 13–14 at the Borg Rating of Perceived Exertion scale). Pedal frequency is held constant at both work rates (60 revolutions per minute) and heart rate is measured as the mean during the last minute at each work rate. The heart rate difference is then related to the increase in work rate between the two work rates, and introduced in sex-specific validated algorithms for estimation of VO_2_max. The test has shown high validity in reference to direct measurement of VO_2_max in this age-group (r^2^=0.84, SE of estimate 0.33 L/min).^14^ Submaximal testing of CRF has been shown to predict all-cause mortality and cardiovascular disease (CVD) events in large, contemporary populations,^15 16^ which highlights the possibility to use submaximal testing in larger population samples. To minimise well-known errors with submaximal testing, participants were requested to refrain from consuming a heavy meal or drinking coffee during the hours before the test. A priori exclusion criteria included symptoms of ongoing infections, known unstable CVD, electrocardiography patterns indicative of cardiac disease, use of beta-blockers, weight >125 kg or resting heart rate of >100 bpm (n=466). Moreover, participants did not take part in the testing if refrained from participating in testing (n=1209), did not receive a valid fitness test due to lack of test criteria fulfilment, did not fulfil the test or malfunction of heart rate monitors or the ergometer (n=394).

### Assessment of anthropometrics and chronic conditions

Measurements of weight, height and waist circumference were assessed during the first visit to the test centre. Body mass index (BMI) was calculated (kg/m^2^), and waist circumference was measured at the midpoint between the top of the iliac crest and the lower margin of the lowest palpable rib. Prevalent depressive symptoms lasting 2 weeks or more during the last 12 months were self-reported. Prevalent chronic conditions were defined as reporting diagnosed disease or surgical treatment for CVD (ie, myocardial infarction, angina pectoris, stroke, congestive heart failure, atrial fibrillation), hypertension, lung disease, dyslipidaemia, diabetes, rheumatic disease and/or cancer.

### Assessment of physical activity pattern

Self-reported PA included commuting habits, physical working situation, exercise, leisure time sitting and total PA (see online supplemental additional file 1). Sedentary behaviour and PA were also derived from triaxial accelerometers (ActiGraph model GT3X and GT3X+, ActiGraph, Pensacola, Florida, USA). Participants were instructed to wear the accelerometer on an elastic belt over the right hip during all waking hours for at least seven consecutive days, except during water-based activities. ActiLife V.6.10.1 software was used to initialise the accelerometers and to download and process the collected data. The accelerometer recorded raw data (30 Hz) from all three axes, which were combined into a resulting vector, and extracted as 60 s epochs using a low frequency extension filter. Sedentary was defined as <200 counts per minute (cpm),^17^ low intensity PA as 200–2689 cpm, moderate intensity PA as 2690–6166 cpm and vigorous PA (VPA) as ≥6167 cpm.^18^ Non-wear time was defined as ≥60 consecutive minutes with no movement (0 cpm), with allowance for maximum 2 min of counts between 0 and 200 cpm. Wear time was calculated as 24 hours minus non-wear time. A minimum of 600 min of valid daily wear time for at least 4 days was required for inclusion.^19^ Prolonged sedentary time was defined as ≥20 min of cpm below 200, with no allowance for interruption above threshold.

10.1136/bmjopen-2022-066336.supp1 Supplementary data

### Assessment of sociodemographic, lifestyle and perceived symptoms and health

Marital status, country of birth, educational level, employment status, financial strain and smoking status were self-reported and grouped according to definitions presented in table 1. Pack-years of smoking was derived by multiplying the average number of cigarettes smoked per day by the number of years smoking, divided by the number of cigarettes per package. The 10-item screening tool Alcohol Use Disorders Identification Test (AUDIT) was used to define alcohol abuse,^20^ and categorised into three groups, ‘No/Low’, ‘Moderate’ and ‘High’, according to the AUDIT score 0–7, 8–15 and >15 for men, and 0–5, 6–13 and >13 for women. Sleep, stress, perceived control at work and in leisure-time, as well as general health, were also self-reported and grouped as presented in table 2.

**Table 1:** Estimated VO_2_max (mL/kg/min) in association with sociodemographic factors and lifestyle behaviour correlates

	n	Estimated VO_2_max	Low estimated VO_2_max	
		Median (95% CI)	% within subgroup	OR* (95% CI)
Sex				
Men	2590	36.2 (35.8 to 36.5)†	na‡	na
Women	2718	30.4 (30.1 to 30.7)	na	na
Age				
50–54 years	1881	34.9 (34.5 to 35.1)§	24	1 (ref)
55–59 years	1812	33.7 (33.2 to 34.0)	32	1.46 (1.27 to 1.69)
60–64 years	1615	31.5 (31.0 to 31.8)	45	2.57 (2.23 to 2.97)
Marital status				
Married/cohabitation	3766	33.8 (33.6 to 34.1)†	31	1 (ref)
Divorced/single/widower	1461	32.1 (31.7 to 32.6)	38	1.37 (1.21 to 1.56)
Born in Sweden				
Yes	4341	33.7 (33.4 to 33.9)†	31	1 (ref)
No	923	32.0 (31.3 to 32.5)	43	1.75 (1.51 to 2.03)
Educational level				
University degree	2480	34.3 (33.9 to 34.6)§	26	1 (ref)
High school/vocational education	2215	32.8 (32.4 to 33.2)	38	1.85 (1.63 to 2.10)
Elementary school	555	31.6 (30.8 to 32.4)	50	2.82 (2.32 to 3.42)
Employment				
Working, ≥50% of full-time	4342	33.9 (33.6 to 34.1)§	30	1 (ref)
Retired	231	30.3 (29.8 to 31.4)	53	1.55 (1.17 to 2.05)
Disability pension/sickness pension/sick leave	299	29.3 (28.4 to 30.2)	54	2.60 (2.05 to 3.31)
Unemployed/student	365	31.3 (30.9 to 32.2)	45	1.88 (1.51 to 2.35)
Financial strain				
No	4759	33.6 (33.4 to 33.9)†	32	1 (ref)
Yes	406	30.1 (29.6 to 30.7)	51	2.51 (2.04 to 3.10)
Smoking habits				
Never smoker	2463	34.6 (34.2 to 34.9)§	29	1 (ref)
Former smokers	2015	32.3 (31.9 to 32.6)	38	1.38 (1.21 to 1.57)
Regular smoker/sometimes	716	32.2 (31.8 to 32.9)	39	1.61 (1.35 to 1.92)
Pack-years				
Never smokers (0)	2463	34.6 (34.2 to 34.9)	29	1 (ref)
Former smokers, low (<15 pack-years)	1220	33.0 (32.6 to 33.7)	30	1.00 (0.86 to 1.17)
Former smokers, heavy (≥15 pack-years)	795	31.1 (30.5 to 31.6)	49	2.17 (1.83 to 2.56)
Current smokers, low (<15 pack-years)	226	33.6 (32.4 to 34.8)	30	1.11 (0.82 to 1.50)
Current smokers, heavy (≥15 pack-years)	490	31.8 (31.1 to 32.3)	42	1.88 (1.53 to 2.30)
Alcohol use				
No/low	3948	33.7 (33.4 to 34.0)	30	1 (ref)
Moderate	639	33.3 (33.0 to 33.9)	34	1.26 (1.05 to 1.51)
High	33	32.5 (30.0 to 33.9)	52	3.06 (1.51 to 6.19)

*Adjusted for sex and age, comparing the lowest sex-specific tertile of estimated VO_2_max with the two higher tertiles.

†Significantly different from other subgroup (Mann-Whitney U test, p<0.0001).

‡Percentage in low estimated VO_2_max and OR not applicable for comparison between men and women, as tertiles were sex-specific.

§Kruskal-Wallis analysis of variance p<0.0001.

**Table 2:** Estimated VO_2_max (mL/kg/min) in association with perceived health, anthropometrics and chronic condition correlates

		Estimated VO_2_max	Low estimated VO_2_max	
	n	Median (95% CI)	% within subgroup	OR* (95% CI)
Sleep				
Very well/well	2401	34.2 (33.9 to 34.5)†	31	1 (ref)
Rather well/badly/very badly	2835	32.6 (32.2 to 32.9)	35	1.22 (1.09 to 1.38)
Stress				
No stress/some stress during last 5 years	4089	33.7 (33.4 to 33.9)†	33	1 (ref)
Constant stress last 1–5 years	1129	32.2 (31.8 to 32.6)	36	1.25 (1.08 to 1.44)
Control at work				
Strongly agree/agree	3846	33.8 (33.6 to 34.1)†	31	1 (ref)
Neutral/do not agree/do not agree at all	1111	32.3 (31.9 to 32.8)	35	1.23 (1.06 to 1.42)
Control in life				
Do not agree at all/do not agree	2976	34.0 (33.7 to 34.3)†	30	1 (ref)
Neutral/agree/strongly agree	2193	32.5 (32.1 to 32.8)	37	1.46 (1.29 to 1.64)
General health				
Excellent/very good	2698	35.0 (34.8 to 35.3)‡	23	1 (ref)
Good	1818	32.5 (32.2 to 33.0)	40	2.41 (2.11 to 2.75)
Somewhat bad/bad	729	29.4 (28.8 to 30.1)	56	4.80 (4.02 to 5.73)
Body mass index with estimated VO_2_max in L/min				
<18	11	2.09 (1.79 to 2.55)‡	55	0.65 (0.19 to 2.29)
18.0–24.9	2025	2.38 (2.35 to 2.40)	36	1 (ref)§
25.0–29.9	2341	2.75 (2.70 to 2.80)	33	2.32 (1.95 to 2.76)
30.0–34.9	728	2.63 (2.52 to 2.73)	29	5.79 (4.30 to 7.81)
≥35	203	2.38 (2.29 to 2.49)	29	16.6 (10.2 to 27.1)
Waist circumference				
Low waist (<88 cm women, <102 cm men)	3191	36.4 (36.2 to 36.7)†	15	1 (ref)
High waist (≥88 cm women, ≥102 cm men)	2114	28.9 (28.6 to 29.2)	61	10.2 (8.8 to 11.7)
Depression symptoms				
No	3687	33.9 (33.7 to 34.2)†	32	1 (ref)
Yes	1495	32.0 (31.6 to 32.3)	37	1.33 (1.17 to 1.51)
Chronic conditions¶				
0	3465	33.9 (33.7 to 34.3)‡	30	1 (ref)
1–2	1705	32.3 (31.9 to 32.8)	39	1.38 (1.22 to 1.57)
≥3	138	31.0 (29.7 to 32.7)	51	2.09 (1.48 to 2.96)
Cardiovascular disease, hypertension and/or lung disease				
No	4069	33.7 (33.5 to 34.0)	32	1 (ref)
Yes	1239	32.0 (31.5 to 32.5)	37	1.42 (1.24 to 1.62)

*Adjusted for sex and age, comparing the lowest sex-specific tertile of estimated VO_2_max with the two higher tertiles.

†Significantly different from other subgroup (Mann-Whitney U test, p<0.0001).

‡Kruskal-Wallis analysis of variance p<0.0001.

§Sex-specific tertiles based on L/min. Analyses additionally adjusted for weight in kg.

¶Includes cardiovascular disease, hypertension, lung disease, dyslipidaemia, diabetes, rheumatic disease and cancer.

### Patient and public involvement

No patient involved.

### Statistical analysis

CRF was skewed (Kolmogorov-Smirnov test), hence, descriptive data are presented as medians and 95% CIs. Low CRF was defined as the lowest sex-specific tertile (cut-off 27.75 mL/kg/min for women and 33.65 mL/kg/min for men). ORs with 95% CIs for low CRF in relation to the subgroups of the correlates were calculated using binary logistic regression modelling adjusting for sex and age. Interaction between sex and each correlate was assessed by adding an interaction term to the model, and significant interactions were defined as p<0.05 for the interaction term. To study independent associations of correlates with low CRF, all correlates not displaying multicollinearity (Spearman’s ρ>0.6) were entered in a backward logistic multivariable regression model, with low CRF as the dependent variable. To study whether the relationship between age-group and estimated VO_2_max was modified by VPA or prolonged sedentary, general linear modelling with post hoc analyses was used including an interaction term for age-group×VPA or age-group×prolonged sedentary, and adjusting for sex. The VPA variable was divided into four groups: 0 min/week, 0 to 37.5 min/week (half of the recommended VPA level), >37.5 to <75 min/week (the recommended VPA level) and ≥75 min/week.^21^ For sedentary behaviour, the quartiles from table 3 were used. Statistical analyses were performed using SPSS (V.26.0, 2019, SPSS, Chicago, Illinois, USA) and R (R V.4.1.1) with R packages ggplot2 and ggeffects.

**Table 3:** Estimated VO_2_max (mL/kg/min) in association with self-reported and accelerometer-derived physical activity pattern correlates

	n	Estimated VO_2_max	Low estimated VO_2_max	
		Median (95% CI)	% within subgroup	OR^C^ (95% CI)
Self-report				
Commuting habits*				
No active commuting	3057	33.1 (32.8 to 33.4)†	37	1 (ref)
Any active commuting, further divided into:	1738	34.6 (34.2 to 35.0)	23	0.47 (0.41 to 0.54)
Partly active commuter, bike	671	34.8 (34.1 to 35.5)	20	0.41 (0.33 to 0.50)
Partly active commuter, walking	196	30.4 (29.3 to 31.6)	41	1.12 (0.83 to 1.52)
Partly active commuter, mix	19	30.2 (28.8 to 33.5)	21	0.45 (0.15 to 1.38)
All year active commuter, bike	407	38.1 (36.9 to 39.2)	10	0.18 (0.13 to 0.25)
All year active commuter, walking	415	33.0 (32.2 to 33.4)	31	0.71 (0.57 to 0.89)
All year active commuter, mix	30	34.7 (30.6 to 37.3)	23	0.51 (0.22 to 1.22)
Physical working situation				
Sedentary to light	3921	33.7 (33.5 to 34.1)‡	30	1 (ref)
Sometimes to frequently heavy	945	32.5 (32.0 to 33.1)	38	1.44 (1.24 to 1.67)
Exercise habits				
Never	1384	31.0 (30.7 to 31.4)†	51	1 (ref)
Irregular	1067	31.3 (30.8 to 31.7)	43	0.75 (0.64 to 0.88)
1–2 times per week	1032	33.5 (33.0 to 34.1)	27	0.35 (0.29 to 0.41)
2–3 times per week	1089	35.2 (34.8 to 35.7)	20	0.25 (0.21 to 0.30)
>3 times per week	649	38.3 (37.8 to 38.9)	12	0.14 (0.11 to 0.19)
Total physical activity				
Sedentary	540	30.0 (29.1 to 30.5)†	63	1 (ref)
Light	2366	31.5 (31.2 to 31.9)	42	0.39 (0.32 to 0.48)
Moderate	1613	34.9 (34.6 to 35.4)	21	0.14 (0.11 to 0.18)
Regular exercise/training	669	38.6 (38.0 to 39.1)	9	0.06 (0.05 to 0.09)
Leisure time sitting				
Q1; <4 hours per day	1121	34.1 (33.5 to 34.7)	28	1 (ref)
Q2; 4–6 hours per day	1039	33.6 (33.0 to 34.2)	32	1.24 (1.02 to 1.49)
Q3; 6–8.5 hours per day	925	34.0 (33.5 to 34.5)	29	1.11 (0.91 to 1.36)
Q4; >8.5 hours per day	958	33.7 (33.3 to 34.2)	32	1.31 (1.08 to 1.59)
% of time spent in sedentary				
Q1; <47%	1267	34.1 (33.5 to 34.5)†	26	1 (ref)
Q2; 47%–54%	1265	33.6 (33.1 to 34.1)	28	1.19 (0.99 to 1.42)
Q3; 54%–61%	1266	33.4 (33.0 to 33.7)	33	1.52 (1.27 to 1.81)
Q4; >61%	1267	33.0 (32.3 to 33.3)	43	2.44 (2.05 to 2.91)
% of time spent in prolonged sedentary (of total wear time)				
Q1; <13%	1266	34.1 (33.6 to 34.5)†	25	1 (ref)
Q2; 13%–19%	1267	33.5 (33.0 to 34.0)	28	1.14 (0.96 to 1.37)
Q3; 19%–26%	1265	33.5 (33.1 to 34.0)	35	1.60 (1.35 to 1.91)
Q4; >26%	1267	32.7 (32.2 to 33.1)	42	2.18 (1.83 to 2.60)
% of time spent in light physical activity				
Q1; <33%	1267	33.7 (33.2 to 34.0)	39	1 (ref)
Q2; 33%–40%	1266	33.3 (32.9 to 33.7)	34	0.78 (0.66 to 0.92)
Q3; 40%–46%	1265	33.5 (33.1 to 34.0)	29	0.60 (0.51 to 0.72)
Q4; >46%	1267	33.2 (32.8 to 33.8)	28	0.56 (0.47 to 0.66)
% of time spent in moderate physical activity				
Q1; <3.8%	1267	31.6 (31.6 to 32.5)†	44	1 (ref)
Q2; 3.8%–5.3%	1265	33.3 (32.7 to 33.7)	32	0.62 (0.52 to 0.73)
Q3; 5.3%–7.2%	1266	33.8 (33.3 to 34.1)	29	0.52 (0.44 to 0.62)
Q4; >7.2%	1267	34.9 (34.3 to 35.3)	26	0.44 (0.37 to 0.52)
% of time spent in vigorous physical activity				
Q1; <0.02%	1266	30.5 (30.1 to 31.0)†	49	1 (ref)
Q2; 0.02%–0.1%	1266	31.7 (31.3 to 32.1)	43	0.80 (0.69 to 0.94)
Q3; 0.1%–0.6%	1267	34.3 (34.0 to 34.7)	23	0.34 (0.28 to 0.40)
Q4; >0.6%	1266	36.7 (36.3 to 37.1)	16	0.21 (0.18 to 0.26)

^c^Adjusted for sex and age, comparing the lowest sex-specific tertile of estimated VO2maxwith the two higher tertiles.

*Analysed both as comparing No active commuting and Any active commuting, as well as further dividing Any active commuting into; Partly commuting, defined as going by bike or walking at least one season out of four; All year commuting, defined as going by bike or walking all year around. Partly and all year commuting ‘walking’ or ‘bike’ were defined as the majority of seasons commuted with that mode of active commuting. Mix was defined when the seasons of active commuting were spent equally between the two modes.

†Kruskal-Wallis analysis of variance p<0.0001.

‡Significantly different from other subgroup (Mann-Whitney U test, p<0.0001).

## Results

Of the total 7377 participants eligible for exercise testing in the study, 5308 provided valid estimates for VO_2_max. Participants included, compared with participants not included (n=2069), were more often women (54% vs 51%, p=0.040), younger (age 57.2 vs 58.2 years, p<0.001), had lower BMI (26.5 vs 27.7 kg/m^2^, p<0.001), had more often university degree (47% vs 35%, p<0.001) and more often reported to be weekly regular exercisers (53% vs 40%, p<0.001). Additionally, among included participants, 14% were regular smokers, 2.3% had established CVD, 17% had hypertension, 11% had dyslipidaemia and 2.5% had diabetes (all self-reported).

Associations between CRF and the different correlates are presented in tables 1–3 and online supplemental appendix figures 1 and 2, with sex-specific analyses presented in online supplemental appendix table 1. The results can be summarised as follows:

10.1136/bmjopen-2022-066336.supp2 Supplementary data

Sociodemographic factors and lifestyle behaviours (table 1): Women, older age, divorced/single/widowed, participants not born in Sweden, lower education, employment <50% of full-time/retired/pension/unemployed, with financial strain or being regular and former smokers had lower estimated CRF and higher OR for having low CRF, compared with their counterparts. Older men (60–64 years) were more likely to have lower CRF than older women (p=0.045), with no other interactions seen between sex and the other sociodemographic variables. For lifestyle factors, there were significant interactions between sex and smoking, such that men being regular smokers/sometimes smokers, and with more pack-years in life, were more likely to have low CRF (p=0.004 and p=0.008) compared with smoking women.

Perceived health, anthropometrics and chronic conditions (table 2 and online supplemental appendix figure 1): Participants reporting poor sleep, high stress, low control at work, low perceived degree of control in life, poorer general health, having higher BMI, higher waist circumference, prevalence of depression symptoms and higher number of chronic conditions had lower estimated CRF. Women with low perceived degree of control in life were more likely to have low CRF than their male counterparts (p=0.004). Higher BMI and higher waist circumference were more strongly associated with low CRF in women than in men (p=0.001 and p=0.041).

Self-reported and accelerometer-derived PA pattern (table 3 and online supplemental appendix figure 2): Participants engaging in any active commuting had higher CRF compared with those with no active commuting. This was mainly seen among partly-year and all-year commuters by bike. Participants with a more strenuous physical working situation, less weekly exercise, lower self-reported total PA level and high self-reported sitting time had lower CRF. Women reporting higher total PA had lower OR for low CRF (p=0.021) compared with men. Higher accelerometer-derived time spent in sedentary and prolonged sedentary time were associated with lower CRF, whereas more time spent in low intensity PA, moderate PA and VPA were associated with higher CRF. Women with higher sedentary time were more likely to have lower CRF compared with men (p=0.033).

**Figure 1:**
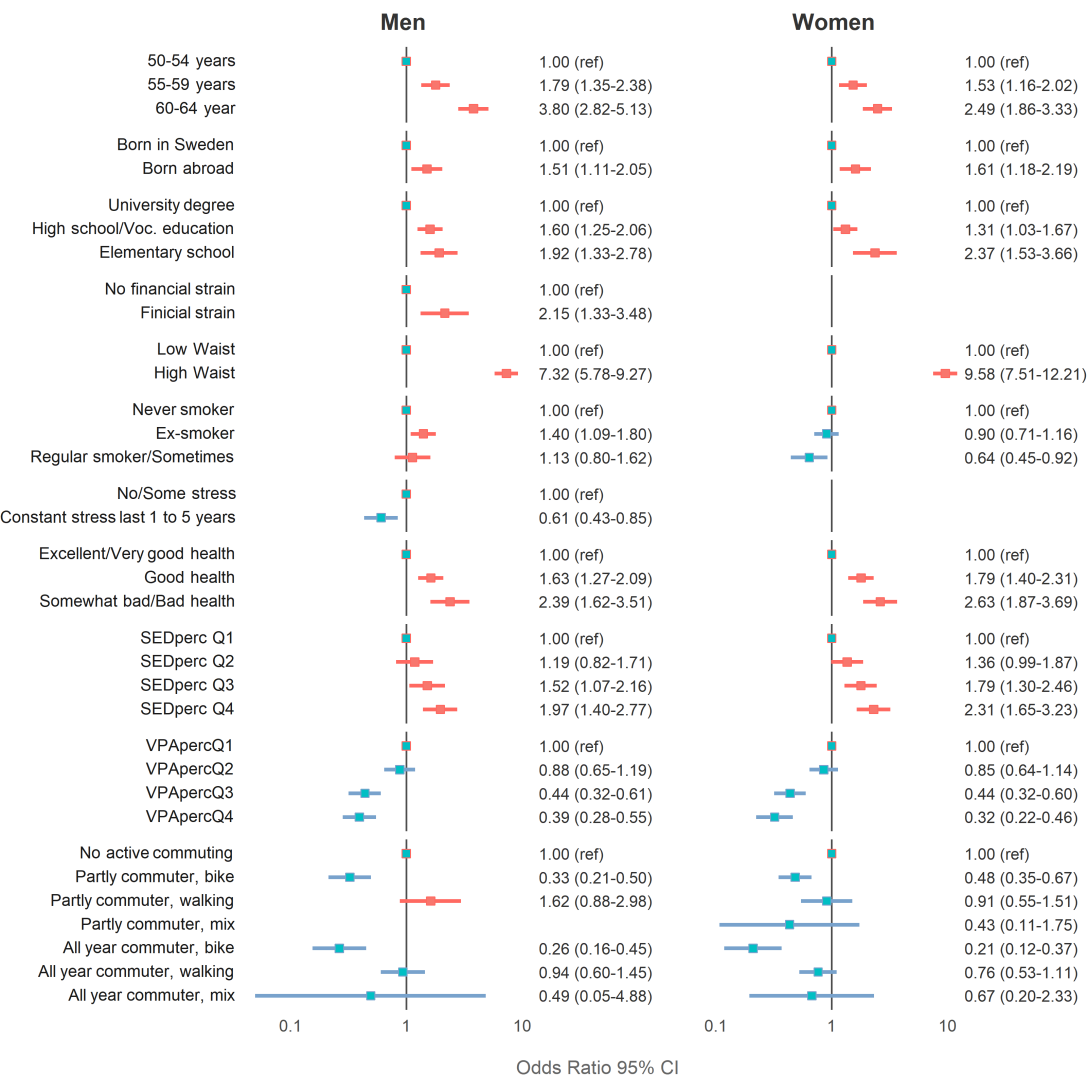
ORs (95% CIs) for low cardiorespiratory fitness in association with correlates in the backward multiregression model. ORs are presented on log-scale. perc, per cent; Q, quartile; SED, sedentary; voc, vocational; VPA, vigorous physical activity.

In the multivariable regression model (figure 1), older age-group, being born abroad, having lower education, high waist circumference, poorer general health and more time spent sedentary, were associated with a higher OR for low CRF in both men and women. More time spent in VPA and commuting by bike (partly and all-year) were associated with a lower OR. Specifically in men, financial strain and being an ex-smoker were associated with higher OR, and constant stress with a lower OR, for a low CRF. Specifically in women, being a regular smoker was associated with lower OR for low CRF, with no significant associations with financial strain or stress.

**Figure 2:**
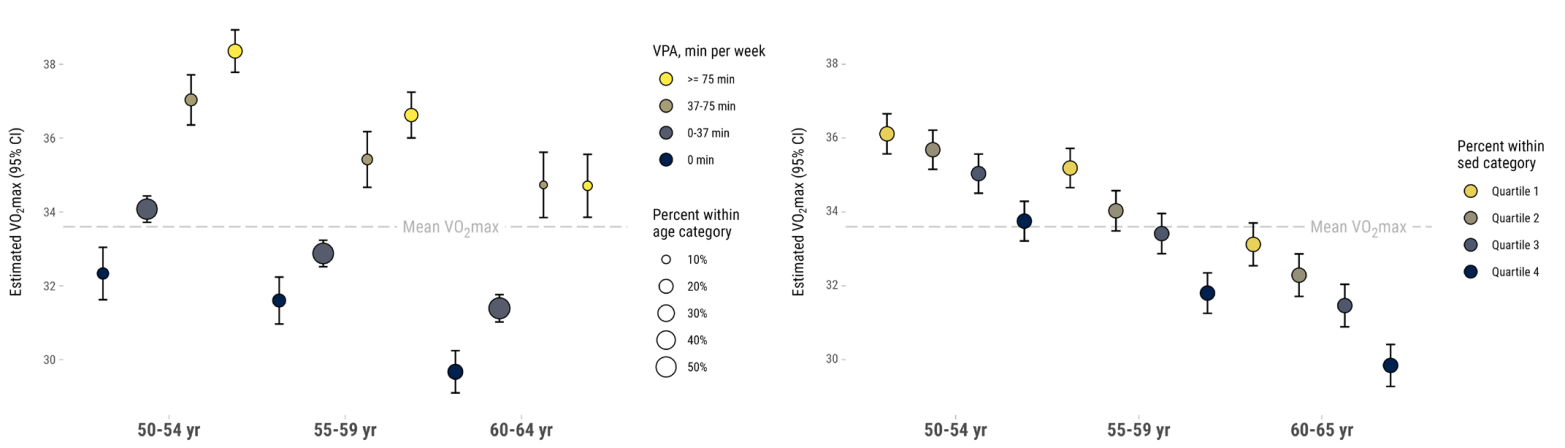
Mean estimated VO_2_max (95% CI) in association with age-group (x-axis) and vigorous physical activity (VPA) level in min/week (left) and percentage of time in sedentary (right).

In figure 2, higher levels of VPA were more common in younger age-groups (left part of figure). However, higher levels of VPA are associated with significantly higher CRF in all age-groups (p<0.001). There was no overall interaction between VPA and age-group (p=0.372). Stratified analyses showed that there was no significant difference in CRF between participants with 37.5–75 min and ≥75 min of VPA per week in the oldest age-group (p=1.00). Also, participants aged 50–54 years and 55–59 years with 0 min/week of VPA had similar CRF (p=0.305 for differences between groups), and so did participants aged 55–59 and 60–64 years with 37.5–75 min/week (p=1.000 for differences between groups). In the right part of figure 2, higher levels of time spent sedentary were associated with lower CRF in all age-groups. There was no overall interaction effect between sedentary time and age-group (p=0.596). Stratified analyses showed that there was some overlapping between age-groups and time sedentary for CRF.

## Discussion

The present study identified multiple correlates of CRF in a large, population-based sample of middle-aged, urban Swedish adults from the SCAPIS study. The results confirm some previously documented correlates, but also adds important novel aspects, including correlates of depression symptoms, commuting habits and accelerometer-derived PA. Some of the most interesting correlates are discussed, as follows:

First, the strong, positive association between CRF and length of education confirms previous results,^8–10^ while similar associations with being born in Sweden, being employed and having an economic buffer, adds novel information regarding sociodemographic aspects. These associations may largely be mediated by level of VPA. Individuals in these subgroups have previously been shown to engage in higher levels of daily VPA compared with their counterparts,^22^ and a strong agreement between socioeconomic factors and both PA and CRF has been reported.^8^ The association between lower CRF in groups with lower socioeconomic status is also in line with higher observed incidence of cardiovascular, mood-related disorders and premature mortality in lower socioeconomic strata compared with those of higher socioeconomic status.^23^ Also, in a recent paper on risk of severe COVID-19, the associations between socioeconomic factors such as educational level, income and occupational group and severe COVID-19 risk, were largely mediated by the CRF level of the participants.^24^

Second, participants reporting more physically demanding work had in general a lower CRF, compared with more sedentary workers. While this is in line with previous reports from the Swedish working population,^25^ this may at first be counterintuitive as higher occupational PA could be considered as a ‘work-out’ contributing to health benefits and higher CRF. However, although more physically demanding occupations induce a higher mean intensity compared with more sedentary occupations, the relative intensity of ‘active jobs’ have been shown to be maybe too low to improve VO_2_max.^26^ Rather, the higher mean intensity may be tiring enough for the individual, which could lead to lower levels of fitness-enhancing leisure time VPA.^22^ This, together with a more pronounced increase in body weight in blue-collar (more active) occupational groups,^27^ may contribute to the general lower CRF levels in these groups. Also, the mean intensity during work has been associated with lower heart rate variability and a higher heart rate the following night.^26^ This suggests an induced imbalance in the autonomic cardiac modulation, which may be one potential underlying mechanism for the detrimental effects on health proposed for occupational PA, also called the ‘physical activity paradox’.^28^ Thus, differences in CRF may partly contribute to the observed paradox.

Third, we could confirm the established relation between self-reported total and leisure-time PA and CRF,^9^ however, expanding on knowledge regarding CRF in active commuters. Any active commuting was associated with higher CRF, however, the higher CRF was mainly driven by partly and all-year commuters by bike. This is line with higher levels of VPA shown in active commuters.^22^ Although the present study cannot clarify whether it was the active commuting per se that contributed to higher CRF levels, mean intensity during normal commuting in habitual cycle commuters has been shown to be mainly constituted of the lower end of vigorous intensity^29^ for longer activity bouts (>10 min). This then often adds up to recommended levels of 150 min per week.^30^ Thus, regular active commuting by bike requires an at least moderate or even high CRF.

Fourth, previous data on correlates between CRF and accelerometer-assessed PA patterns are scarce and inconclusive.^9^ We found that higher accelerometer-derived estimates of the different components of the PA pattern provided a strong association between CRF level and time in sedentary (negative association), and MPA and VPA (positive associations). Interestingly, both time in sedentary and in VPA remained significantly associated with CRF in the multivariable regression model, which implies separate importance of these intensities for CRF level.

Finally, previous research is unclear about whether higher PA level would ameliorate lower CRF levels among older individuals.^9^ The cross-sectional analyses in our study showed that older individuals had lower CRF than younger, with strong associations between higher VPA and less time spent sedentary, and CRF in all age-groups. More time in VPA may be required for older individuals in order to maintain a certain CRF, as the mean estimated VO_2_max in individuals 60–64 years old engaging in 37 min or more of VPA per week was overlapping with the VO_2_max in individuals 50–54 years old doing less than 37 min of VPA per week. It is also possible that relations are reversed or at least bidirectional, that is, that a higher CRF will enable an individual to be physically active, including higher intensity activities. Indeed, previous studies have shown that those being active at higher age, have superior CRF compared with less active peers.^12^ Age-related medico-physiological changes, possibly affecting the possibility to be active, such as loss of muscle mass or increasing concomitant disease and medications, may affect the intensity of the VPA performed.^7^

### Strengths and limitations

The cross-sectional design of the study prevents any causal inference and we cannot rule out reverse causality. We have no information on any genetic contribution to variation between subgroups of correlates. Although we have a large sample from the general population, those who participated in the fitness testing did differ from the source population. Therefore, translation of the results to populations that are less healthy and active and of other socioeconomic status (eg, lower education), should be done with caution. Further, the large sample size with estimated CRF from a submaximal test and accelerometer-measured PA provided the power required to analyse multiple correlates relevant for CRF. It is unknown if regular bicycling affects validity of a cycle ergometer test. Local adaptations to the thigh and gluteal muscles from regular cycling may alter systemic circulatory response to standard cycling exercise. If so, the finding that regular bike commuters had higher CRF may in part be ascribed to varying validity.

## Conclusions

This study in a large sample of middle-aged men and women delineates multiple modifiable as well as non-modifiable correlates of CRF. In addition, we identified specific subgroups as potential high-risk groups for CRF-related disorders, which constitute targets for behavioural change to increase CRF, if relations are causal. These groups include individuals with higher age, being born abroad, lower education, high waist circumference, poor perceived health, high accelerometer-derived time in sedentary and low in VPA, as well as passive commuters. Across all age-groups, higher VPA and less time spent sedentary, were associated with a higher CRF. Although having limited possibilities for causal inference, the present study provides important reference material of CRF and correlates in a general middle-aged population, which can be valuable for future research, clinical practice and public health work. Specifically, increased knowledge about specific subgroups will aid in the personalised prescription of exercise in healthcare. In light of the higher prevalence of low CRF in later years,^5^ a great challenge remains in implementing effective interventions to support health in identified parts of the population.

## Supplementary Material

Reviewer comments

Author's manuscript

## Data Availability

Data are available upon reasonable request. Due to the nature of the sensitive personal data and study materials, they cannot be made freely available. However, by contacting the corresponding author or study organisation (www.scapis.org), procedures for sharing data, analytical methods and study materials for reproducing the results or replicating the procedure can be arranged following Swedish legislation.

## References

[1] Bouchard C, An P, Rice T, et al. Familial aggregation of VO(2max) response to exercise training: results from the HERITAGE Family Study. J Appl Physiol 1999;87:1003–8. 10.1152/jappl.1999.87.3.1003 10484570

[2] Kodama S, Saito K, Tanaka S, et al. Cardiorespiratory fitness as a quantitative predictor of all-cause mortality and cardiovascular events in healthy men and women: a meta-analysis. JAMA 2009;301:2024–35. 10.1001/jama.2009.681 19454641

[3] Kandola A, Ashdown-Franks G, Stubbs B, et al. The association between cardiorespiratory fitness and the incidence of common mental health disorders: a systematic review and meta-analysis. J Affect Disord 2019;257:748–57. 10.1016/j.jad.2019.07.088 31398589 PMC6997883

[4] Laukkanen JA, Isiozor NM, Kunutsor SK. Objectively assessed cardiorespiratory fitness and all-cause mortality risk: an updated meta-analysis of 37 cohort studies involving 2,258,029 participants. Mayo Clin Proc 2022;97:1054–73. 10.1016/j.mayocp.2022.02.029 35562197

[5] Ekblom-Bak E, Ekblom Örjan, Andersson G, et al. Decline in cardiorespiratory fitness in the Swedish working force between 1995 and 2017. Scand J Med Sci Sports 2019;29:232–9. 10.1111/sms.13328 30351472 PMC7379642

[6] Lamoureux NR, Fitzgerald JS, Norton KI, et al. Temporal trends in the cardiorespiratory fitness of 2,525,827 adults between 1967 and 2016: a systematic review. Sports Med 2019;49:41–55. 10.1007/s40279-018-1017-y 30390202

[7] Katzel LI, Sorkin JD, Fleg JL. A comparison of longitudinal changes in aerobic fitness in older endurance athletes and sedentary men. J Am Geriatr Soc 2001;49:1657–64. 10.1111/j.1532-5415.2001.49276.x 11844000

[8] Lindgren M, Börjesson M, Ekblom Örjan, et al. Physical activity pattern, cardiorespiratory fitness, and socioeconomic status in the SCAPIS pilot trial - A cross-sectional study. Prev Med Rep 2016;4:44–9. 10.1016/j.pmedr.2016.04.010 27413660 PMC4929080

[9] Zeiher J, Ombrellaro KJ, Perumal N, et al. Correlates and determinants of cardiorespiratory fitness in adults: a systematic review. Sports Med Open 2019;5:39. 10.1186/s40798-019-0211-2 31482208 PMC6722171

[10] Ombrellaro KJ, Perumal N, Zeiher J, et al. Socioeconomic correlates and determinants of cardiorespiratory fitness in the general adult population: a systematic review and meta-analysis. Sports Med Open 2018;4:25. 10.1186/s40798-018-0137-0 29882063 PMC5992110

[11] Ekblom Örjan, Ekblom-Bak E, Bolam KA, et al. Concurrent and predictive validity of physical activity measurement items commonly used in clinical settings--data from SCAPIS pilot study. BMC Public Health 2015;15:978. 10.1186/s12889-015-2316-y 26415512 PMC4587776

[12] Hawkins S, Wiswell R. Rate and mechanism of maximal oxygen consumption decline with aging: implications for exercise training. Sports Med 2003;33:877–88. 10.2165/00007256-200333120-00002 12974656

[13] Bergström G, Berglund G, Blomberg A, et al. The Swedish cardiopulmonary BioImage study: objectives and design. J Intern Med 2015;278:645–59. 10.1111/joim.12384 26096600 PMC4744991

[14] Björkman F, Ekblom-Bak E, Ekblom Örjan, et al. Validity of the revised Ekblom Bak cycle ergometer test in adults. Eur J Appl Physiol 2016;116:1627–38. 10.1007/s00421-016-3412-0 27311582 PMC4983286

[15] Laukkanen JA, Kunutsor SK, Yates T, et al. Prognostic relevance of cardiorespiratory fitness as assessed by submaximal exercise testing for all-cause mortality: a UK Biobank prospective study. Mayo Clin Proc 2020;95:867–78. 10.1016/j.mayocp.2019.12.030 32370851

[16] Ekblom-Bak E, Ekblom B, Söderling J, et al. Sex- and age-specific associations between cardiorespiratory fitness, CVD morbidity and all-cause mortality in 266.109 adults. Prev Med 2019;127:105799. 10.1016/j.ypmed.2019.105799 31454664

[17] Aguilar-Farías N, Brown WJ, Peeters GMEEG. ActiGraph GT3X+ cut-points for identifying sedentary behaviour in older adults in free-living environments. J Sci Med Sport 2014;17:293–9. 10.1016/j.jsams.2013.07.002 23932934

[18] Sasaki JE, John D, Freedson PS. Validation and comparison of ActiGraph activity monitors. J Sci Med Sport 2011;14:411–6. 10.1016/j.jsams.2011.04.003 21616714

[19] Migueles JH, Cadenas-Sanchez C, Ekelund U, et al. Accelerometer data collection and processing criteria to assess physical activity and other outcomes: a systematic review and practical considerations. Sports Med 2017;47:1821–45. 10.1007/s40279-017-0716-0 28303543 PMC6231536

[20] Babor TF, de la Fuente JR, Saunders J. The alcohol use disorders identification test. guidelines for use in primary health care. World Health Organization, 2001.

[21] Bull FC, Al-Ansari SS, Biddle S, et al. World Health organization 2020 guidelines on physical activity and sedentary behaviour. Br J Sports Med 2020;54:1451–62. 10.1136/bjsports-2020-102955 33239350 PMC7719906

[22] Ekblom-Bak E, Börjesson M, Bergman F, et al. Accelerometer derived physical activity patterns in 27.890 middle-aged adults: the SCAPIS cohort study. Scand J Med Sci Sports 2022;32:866–80. 10.1111/sms.14131 35080270 PMC9302631

[23] Lewer D, Jayatunga W, Aldridge RW, et al. Premature mortality attributable to socioeconomic inequality in England between 2003 and 2018: an observational study. Lancet Public Health 2020;5:e33–41. 10.1016/S2468-2667(19)30219-1 31813773 PMC7098478

[24] Ekblom-Bak E, Väisänen D, Ekblom B, et al. Cardiorespiratory fitness and lifestyle on severe COVID-19 risk in 279,455 adults: a case control study. Int J Behav Nutr Phys Act 2021;18:135. 10.1186/s12966-021-01198-5 34666788 PMC8524225

[25] Väisänen D, Kallings LV, Andersson G, et al. Lifestyle-associated health risk indicators across a wide range of occupational groups: a cross-sectional analysis in 72,855 workers. BMC Public Health 2020;20:1656. 10.1186/s12889-020-09755-6 33148214 PMC7641800

[26] Korshøj M, Lund Rasmussen C, de Oliveira Sato T, et al. Heart rate during work and heart rate variability during the following night: a day-by-day investigation on the physical activity paradox among blue-collar workers. Scand J Work Environ Health 2021;47:387–94. 10.5271/sjweh.3965 33929548 PMC8259705

[27] Väisänen D, Kallings LV, Andersson G, et al. Cardiorespiratory fitness in occupational Groups-Trends over 20 years and future forecasts. Int J Environ Res Public Health 2021;18. doi:10.3390/ijerph18168437. [Epub ahead of print: 10 Aug 2021]. PMC8394663 34444184

[28] Holtermann A, Krause N, van der Beek AJ, et al. The physical activity paradox: six reasons why occupational physical activity (OPA) does not confer the cardiovascular health benefits that leisure time physical activity does. Br J Sports Med 2018;52:149–50. 10.1136/bjsports-2017-097965 28798040

[29] Schantz P, Salier Eriksson J, Rosdahl H. Perspectives on exercise intensity, volume and energy expenditure in habitual cycle Commuting. Front Sports Act Living 2020;2:65. 10.3389/fspor.2020.00065 33345056 PMC7739755

[30] Stigell E, Schantz P. Active commuting behaviors in a Nordic metropolitan setting in relation to modality, gender, and health recommendations. Int J Environ Res Public Health 2015;12:15626–48. 10.3390/ijerph121215008 26690193 PMC4690944

