# Supplementary material for: Correlates of cardiorespiratory fitness in a population-based sample of middle-aged adults: cross-sectional analyses in the SCAPIS study
Source: BMJ Open. 2022 Dec 14;12(12):e066336. doi: 10.1136/bmjopen-2022-066336 (PMC9756301; doi:10.1136/bmjopen-2022-066336)
Supplement: Supplementary data [file bmjopen-2022-066336supp001.pdf]

Additional file 1. Questions from the SCAPIS questionnaire used in the present analyses. Reference to the original question is given if available.

__Educational level__
What is your highest completed level of education?
No formal education degree
Elementary school
High school / Vocational education
University degree

__Employment status__
What is your current occupation?
Employed, _____% of fulltime
On leave / parental leave
Studying
Labour market measure
Unemployed
Retired
Early or sick retiree

__Financial strain__
If you were to suddenly end up in a situation where you had to raise SEK 20,000 (≈EURO 2,000) in one week, would you be able to do it?
Yes
No

Strandhagen, E., et al. (2010). "Selection bias in a population survey with registry linkage: potential effect on socioeconomic gradient in cardiovascular risk." Eur J Epidemiol 25(3): 163-172.

__Marital status__
What is your current marital status?
Alone
Divorced
Married/Cohabitat
Widow

__Born in Sweden__
Are you born in Sweden?
Yes
No

__Smoking habits__
Do you smoke?
No, have never smoked
Yes, smokes regularly
No, stopped smoking
Yes, smokes occasionally

<u>Commuting habits</u>
Mode of travel to work:
Spring
Car
Bus/tram/train
Bicycling
Walking

Summer
Car
Bus/tram/train
Bicycling
Walking

Autumn
Car
Bus/tram/train
Bicycling
Walking

Winter
Car
Bus/tram/train
Bicycling
Walking

Wennberg, P., et al (2006). The effects of commuting activity and occupational and leisure time physical activity on risk of myocardial infarction. European Journal of Cardiovascular Prevention & Rehabilitation December 2006 13: 924-930

<u>Sleep</u>
How well are you sleeping usually?
Very well
Well
Rather well
Badly
Very badly

Adapted from the Basic Nordics Sleep Questionnaire Partinen, M. and T. Gislason (1995). "Basic Nordic Sleep Questionnaire (BNSQ): a quantitated measure of subjective sleep complaints." J Sleep Res 4(S1): 150-155.

<u>Stress</u>
By stress we mean feeling tense, irritable, anxious or having sleeping difficulties as a result of conditions at work or at home. Did you experience this?
Never experienced stress
≥1 period of stress
≥1 period of stress during the last 5 years
several periods of stress during the last 5 years
permanent stress during the last year or the last 5 years

Adapted from: Rosengren, A., et al. (2004). "Association of psychosocial risk factors with risk of acute myocardial infarction in 11119 cases and 13648 controls from 52 countries (the INTERHEART study): case-control study." Lancet 364(9438): 953-962.

Rosengren, A., et al. (1991). "Self-perceived psychological stress and incidence of coronary artery disease in middle-aged men." Am J Cardiol 68(11): 1171-1175.

Control at work

At work I feel that I have control over what happens in most situations

Strongly disagree
Disagree
Neutral
Agree
Strongly agree

Adapted from: Rosengren, A., et al. (2004). "Association of psychosocial risk factors with risk of acute myocardial infarction in 11119 cases and 13648 controls from 52 countries (the INTERHEART study): case-control study." Lancet 364(9438): 953-962.

Bobak, M., et al. (2000). "Socioeconomic factors, material inequalities, and perceived control in self-rated health: cross-sectional data from seven post-communist countries." Soc Sci Med 51(9): 1343-1350. + references therein.

Control in life

I feel that what happens in my life is often determined by factors beyond my control

Strongly disagree
Disagree
Neutral
Agree
Strongly agree

Adapted from: Rosengren, A., et al. (2004). "Association of psychosocial risk factors with risk of acute myocardial infarction in 11119 cases and 13648 controls from 52 countries (the INTERHEART study): case-control study." Lancet 364(9438): 953-962.

Bobak, M., et al. (2000). "Socioeconomic factors, material inequalities, and perceived control in self-rated health: cross-sectional data from seven post-communist countries." Soc Sci Med 51(9): 1343-1350. + references therein.

General health

In general, would you say your health is:

Excellent
Very good
Good
Fair
Poor

Adapted from the Swedish version of the SF-36 questionnaire.
Sullivan, M., et al. (1995). "The Swedish SF-36 Health Survey--I. Evaluation of data quality, scaling assumptions, reliability and construct validity across general populations in Sweden." Soc Sci Med 41(10): 1349-1358.

Sullivan, M. and J. Karlsson (1998). "The Swedish SF-36 Health Survey III. Evaluation of criterion-based validity: results from normative population." J Clin Epidemiol 51(11): 1105-1113.

Persson, L. O., et al. (1998). "The Swedish SF-36 Health Survey II. Evaluation of clinical validity: results from population studies of elderly and women in Gothenborg." J Clin Epidemiol 51(11): 1095-1103

<u>Depression symptoms</u>

During the past 4 weeks, have you had any of the following problems with your work or other regular daily activities as a result of any emotional problems (such as feeling depressed or anxious)?

Yes
No

Adapted from the Swedish version of the SF-36 questionnaire.
Sullivan, M., et al. (1995). "The Swedish SF-36 Health Survey--I. Evaluation of data quality, scaling assumptions, reliability and construct validity across general populations in Sweden." Soc Sci Med 41(10): 1349-1358.

Sullivan, M. and J. Karlsson (1998). "The Swedish SF-36 Health Survey III. Evaluation of criterion-based validity: results from normative population." J Clin Epidemiol 51(11): 1105-1113.
Persson, L. O., et al. (1998). "The Swedish SF-36 Health Survey II. Evaluation of clinical validity: results from population studies of elderly and women in Gothenborg." J Clin Epidemiol 51(11): 1095-1103

<u>Prevalent chronic conditions</u>

Which of these diseases has a doctor diagnosed you or have you been surgically treated for?

Myocardial infarction
Angina pectoris
Atrial fibrillation
Heart failure
Heart valve disease
CABG or PCI
Intervention of arterial stenosis other than coronary artery stenosis
Aortic intervention
Stroke
Hypertension
Dyslipidemia
Diabetes
Doctor-diagnosed COPD, chronic bronchitis or emphysema
Other lung disease
Rheumatic disease
Cancer

<u>Physical working situation</u>

Degree of physical activity at work

Physically heavy most of the time
Sometimes physically heavy
Light and mobile

Light but partly mobile
Sedentary or standing

Wareham, N. J., et al. (2003). "Validity and repeatability of a simple index derived from the short physical activity questionnaire used in the European Prospective Investigation into Cancer and Nutrition (EPIC) study." Public Health Nutr 6(4): 407-413.

InterAct, C., et al. (2012). "Validity of a short questionnaire to assess physical activity in 10 European countries." Eur J Epidemiol 27(1): 15-25.

Exercise habits

How often have you exercised or exercised in workout clothes in the last three months, in order to improve your fitness and/or to feel good?
Never
Occasionally - not regularly
1-2 times a week
2-3 times a week
More than 3 times per week

Wareham, N. J., et al. (2003). "Validity and repeatability of a simple index derived from the short physical activity questionnaire used in the European Prospective Investigation into Cancer and Nutrition (EPIC) study." Public Health Nutr 6(4): 407-413.

InterAct, C., et al. (2012). "Validity of a short questionnaire to assess physical activity in 10 European countries." Eur J Epidemiol 27(1): 15-25.

Total physical activity

How much physical movement and exertion have you had in the last 12 months?
Sedentary
Light exercise
Moderate exercise
Regular, vigorous exercise

Sweden PHao. The Swedish national public health survey. http://www.folkhalsomyndigheten.se/documents/statistik-uppfoljning/enkater-undersokningar/nationella-folkhalsoenkaten/frageformular/formular-nationella-folkhalsoenkaten-2012.pdf. 2012.

Leisure time sitting

The last question is about the time you spent sitting on weekdays during the last 7 days. Include time spent at work, at home, while doing course work and during leisure time. This may include time spent sitting at a desk, visiting friends, reading, or sitting or lying down to watch television. How much time did you spend sitting on a day?
____minutes

Adapted from the IPAQ questionnaire: Craig, C. L., et al. (2003). "International physical activity questionnaire: 12-country reliability and validity." Med Sci Sports Exerc 35(8): 1381-1395.
