# Supplementary material for: Correlates of cardiorespiratory fitness in a population-based sample of middle-aged adults: cross-sectional analyses in the SCAPIS study
Source: BMJ Open. 2022 Dec 14;12(12):e066336. doi: 10.1136/bmjopen-2022-066336 (PMC9756301; doi:10.1136/bmjopen-2022-066336)

Appendix Table 1. Sex-specific estimated VO_2max (ml/min/kg) in association with all correlates presented in Table 1 to 3.

		**Estimated VO_2max**			**Low estimated VO_2max**		
		Men		Women	Men	Women	p-value for interaction
**Age**	n	Median (95% CI)	n	Median (95% CI)	OR[a] (95% CI)	OR (95% CI)	p=0.045
50-54 years	906	37.8 (37.3-38.3)	975	31.9 (31.4-32.3)	1 (ref)	1 (ref)	
55-59 years	885	36.3 (35.7-36.8)	927	30.5 (30.0-31.0)	1.49 (1.21-1.84)	1.44 (1.18-1.75)	
60-64 years	799	33.9 (33.6-34.7)	816	28.6 (28.1-29.1)	2.98 (2.42-3.66)	2.23 (1.83-2.73)	
**Civil status**							
Married/Cohabitation	1935	36.5 (36.0-36.8)	1831	30.7 (30.4-31.2)	1 (ref)	1 (ref)	p=0.655
Divorced/Single/Widower	610	35.3 (34.7-36.1)	851	29.7 (29.1-30.2)	1.43 (1.17-1.73)	1.34 (1.12-1.59)	
**Born in Sweden**							
Yes	2090	36.5 (36.1-36.8)	2251	30.7 (30.4-31.1)	1 (ref)	1 (ref)	p=0.633
No	472	34.9 (34.1-35.5)	451	28.9 (28.3-29.3)	1.85 (1.49-2.28)	1.68 (1.36-2.07)	
**Educational level**							
University degree	1087	37.5 (37.0-38.0)	1393	31.5 (31.2-32.0)	1 (ref)	1 (ref)	p=0.294
High school/Vocational education	1147	35.5 (35.1-35.9)	1068	29.5 (29.1-29.9)	1.86 (1.54-2.24)	1.83 (1.54-2.18)	
Elementary school	321	34.0 (33.3-34.8)	234	27.4 (26.5-28.6)	2.54 (1.95-3.32)	3.21 (2.41-4.28)	
**Employment**							
Working, ≥ 50% of full-time	2117	36.7 (36.5-37.0)	2225	31.0 (30.6-31.4)	1 (ref)	1 (ref)	p=0.574
Retired	123	33.3 (32.1-34.7)	108	27.6 (26.6-29.0)	1.48 (1.01-2.19)	1.60 (1.07-2.41)	
Disability pension/Sickness pension/Sick leave	115	33.7 (32.9-34.8)	184	26.7 (26.0-28.0)	2.16 (1.47-3.18)	2.91 (2.14-3.97)	
Unemployed/Student	191	33.6 (32.6-34.5)	174	29.3 (28.4-30.1)	2.27 (1.67-3.09)	1.53 (1.11-2.11)	

**Financial strain**							
No	2328	36.5 (36.1-36.8)	2431	30.7 (30.1-31.1)	1 (ref)	1 (ref)	p=0.293
Yes	177	33.0 (31.9-34.3)	229	28.0 (26.9-28.7)	2.91 (2.11-4.00)	2.24 (1.69-2.96)	
**Smoking habits**							
Never smoker	1287	37.3 (36.8-37.9)	1176	31.4 (31.0-31.8)	1 (ref)	1 (ref)	p=0.004
Ex-smoker	897	35.0 (34.6-35.5)	1118	29.9 (29.3-30.3)	1.65 (1.37-1.99)	1.17 (0.98-1.39)	
Regular smoker/Sometimes	341	35.1 (34.0-36.1)	375	29.9 (29.3-30.3)	2.02 (1.57-2.60	1.28 (0.99-1.64)	
**Pack-years**							
Never smokers (0)	1287	37.3 (36.8-37.9)	1176	31.4 (31.0-31.8)	1 (ref)	1 (ref)	p=0.008
Former smokers, low (<15 pack-years)	500	36.1 (35.4-36.7)	720	30.5 (30.1-31.1)	1.13 (0.90-1.43)	0.89 (0.73-1.09)	
Former smokers, heavy (≥15 pack-years)	397	33.4 (33.1-34.3)	398	28.1 (27.6-29.1)	2.57 (2.03-3.27)	1.82 (1.43-2.30)	
Current smokers, low (<15 pack-years)	106	36.5 (34.8-37.6)	120	31.6 (30.0-32.9)	1.57 (1.02-2.40)	0.79 (0.51-1.22)	
Current smokers, heavy (≥15 pack-years)	235	34.8 (33.6-35.6)	255	29.3 (28.3-29.9)	2.26 (1.69-3.02)	1.56 (1.18-2.07)	
**Alcohol abuse**							
No/Low	1881	36.6 (36.3-36.9)	2067	31.0 (30.5-31.3)	1 (ref)	1 (ref)	p=0.226
Moderate	355	35.4 (34.8-36.4)	284	30.8 (30.1-31.3)	1.43 (1.12-1.82)	1.08 (0.82-1.42)	
High	22	33.7 (31.1-35.0)	11	27.3 (24.6-33.3)	2.72 (1.13-6.50)	3.92 (1.18-13.04)	
**Sleep**							
Very well/well	1348	36.7 (36.2-37.0)	1053	30.8 (30.3-31.4)	1 (ref)	1 (ref)	p=0.253
Rather well/Badly/Very badly	1198	35.7 (35.4-36.1)	1637	30.2 (29.9-30.5)	1.31 (1.11-1.56)	1.14 (0.97-1.35)	
**Stress**							
No stress/Some stress during last 5 years	2140	36.3 (35.9-36.7)	1949	30.5 (30.3-31.0)	1 (ref)	1 (ref)	p=0.267
Constant stress last 1 to 5 years	400	35.6 (35.1-36.6)	729	29.9 (29.4-30.6)	1.14 (0.91-1.44)	1.32 (1.10-1.58)	

**At work I feel that that I have control in most situations**							
Strongly agree/Agree	1993	36.5 (36.1-36.8)	1853	30.8 (30.4-31.2)	1 (ref)	1 (ref)	p=0.730
Neutral/Do not agree/Do not agree at all	444	35.9 (35.1-36.6)	667	30.1 (29.5-30.9)	1.20 (0.96-1.50)	1.25 (1.04-1.51)	
**I feel that what happens in my life is often determined by factors beyond my control**							
Do not agree at all/Do not agree	1489	36.5 (36.1-36.9)	1487	31.3 (30.9-31.7)	1 (ref)	1 (ref)	p=0.004
Neutral/Agree/Strongly agree	1022	35.9 (35.4-36.4)	1171	29.3 (28.9-29.7)	1.22 (1.03-1.45)	1.72 (1.45-2.02)	
**General health**							
Excellent/Very good	1281	38.0 (37.5-38.3)	1417	32.1 (31.8-32.6)	1 (ref)	1 (ref)	p=0.116
Good	966	34.9 (34.5-35.4)	852	29.1 (28.6-29.7)	2.31 (1.91-2.79)	2.52 (2.08-3.04)	
Somewhat bad/Bad	300	33.3 (32.3-34.0)	429	26.4 (25.7-27.3)	4.13 (3.15-5.41)	5.40 (4.27-6.83)	
**BMI and estVO$_2$max in L·min^{-1}**							
<24.9	748	3.07 (3.03-3.12)	1288	2.16 (2.14-2.18)	1 (ref)	1 (ref)	p=0.001
25.0-29.9	1391	3.16 (3.13-3.19)	950	2.14 (2.12-2.17)	1.87 (1.46-2.40)	2.70 (2.11-3.45)	
30.0-34.9	379	3.21 (3.16-3.27)	349	2.18 (2.13-2.23)	4.15 (2.70-6.37)	7.36 (4.85-11.17)	
≥35	72	3.18 (3.06-3.30)	131	2.20 (2.10-2.25)	12.7 (5.7-28.3)	18.2 (9.63-34.3)	
**Waist circumference**							
Low waist (<88 cm women, <102 cm men)	1725	38.5 (38.2-38.9)	1466	34.0 (33.6-34.4)	1 (ref)	1 (ref)	p=0.041
High waist (≥88 cm women, ≥102cm men)	863	32.0 (31.6-32.5)	1251	26.7 (26.4-27.0)	9.15 (7.52-11.1)	11.5 (9.44-14.1)	
**Depression symptoms**							
No	2003	36.3 (35.9-36.7)	1684	30.8 (30.4-31.3)	1 (ref)	1 (ref)	p=0.230
Yes	519	36.0 (35.4-36.6)	976	29.9 (29.3-30.2)	1.21 (0.99-1.50)	1.41 (1.19-1.67)	
**Chronic conditions***							
0	1693	36.8 (36.4-37.1)	1772	31.1 (30.7-31.4)	1 (ref)	1 (ref)	p=0.186
1-2	816	35.1 (34.7-35.7)	889	29.3 (28.8-29.9)	1.30 (1.09-1.56)	1.46 (1.23-1.74)	

=>3	81	34.0 (32.7-35.3)	57	23.8 (22.6-29.1)	1.62 (1.02-2.56)	2.92 (1.70-5.03)	
**CVD, Hypertension and/or Lung disease**							
No	1980	36.6 (36.2-36.9)	2089	31.0 (30.5-31.3)	1 (ref)	1 (ref)	p=0.142
Yes	610	35.0 (34.6-35.6)	629	28.8 (28.3-29.4)	1.26 (1.04-1.53)	1.58 (1.31-1.90)	
**Commuting***							
No active commuting	1638	35.4 (35.1-35.8)	1419	29.9 (29.5-30.3)	1 (ref)	1 (ref)	p=0.831
Partly commuter, bike	270	38.6 (37.7-39.1)	401	32.1 (31.6-33.0)	0.35 (0.25-0.48)	0.45 (0.34-0.58)	
Partly commuter, walking	68	34.5 (32.7-35.8)	128	28.9 (28.1-29.8)	1.42 (0.86-2.33)	0.99 (0.68-1.44)	
Partly commuter, mix	3	44.2 (38.1-50.9)	16	29.8 (28.2-33.2)	-	0.57 (0.18-1.79)	
All year commuter, bike	203	41.3 (40.5-42.4)	204	35.0 (34.2-36.2)	0.23 (0.15-0.35)	0.14 (0.08-0.23)	
All year commuter, walking	162	36.9 (35.6-38.1)	253	30.3 (29.7-31.3)	0.79 (0.56-1.12)	0.66 (0.49-0.89)	
All year commuter, mix	8	40.2 (36.8-41.1)	22	32.3 (29.0-36.1)	0.26 (0.03-2.18)	0.61 (0.24-1.59)	
**Physical working situation**							
Sedentary to light	1894	36.7 (36.3-37.0)	2027	31.0 (30.5-31.4)	1 (ref)	1 (ref)	p=0.775
Sometimes to frequently heavy	498	35.3 (34.8-36.1)	447	29.8 (29.1-30.4)	1.48 (1.20-1.83)	1.40 (1.12-1.74)	
**Exercise habits**							
Never	722	33.7 (33.4-34.1)	662	27.4 (26.7-28.0)	1 (ref)	1 (ref)	p=0.333
Irregular	496	34.8 (34.0-35.3)	571	28.7 (28.2-29.3)	0.78 (0.62-0.99)	0.71 (0.57-0.90)	
1-2 times per week	456	36.7 (36.1-37.3)	576	30.7 (30.3-31.4)	0.35 (0.27-0.46)	0.34 (0.26-0.43)	
2-3 times per week	507	37.9 (37.2-38.5)	582	33.0 (32.3-33.6)	0.29 (0.22-0.38)	0.22 (0.17-0.28)	
>3 times per week	356	40.5 (39.7-41.0)	293	35.4 (34.6-36.2)	0.14 (0.10-0.20)	0.14 (0.10-0.21)	
**Total physical activity**							
Sedentary	315	32.4 (31.5-33.2)	225	25.5 (24.5-26.5)	1 (ref)	1 (ref)	p=0.021
Light	1098	34.9 (34.6-35.3)	1268	28.8 (28.4-29.1)	0.42 (0.32-0.55)	0.34 (0.25-0.46)	

Moderate	735	37.4 (37.0-38.2)	878	32.7 (32.2-33.2)	0.18 (0.14-0.25)	0.10 (0.07-0.14)	
Regular exercise/training	367	40.9 (40.1-41.5)	302	35.6 (35.0-36.2)	0.06 (0.04-0.10)	0.06 (0.04-0.09)	
**Leisure time sitting**							
Q1; <4 h per day	525	37.5 (37.0-38.1)	596	30.9 (30.1-31.7)	1 (ref)	1 (ref)	p=0.159
Q2; 4-6 h per day	499	36.2 (35.7-37.0)	540	30.8 (30.1-31.4)	1.29 (0.98-1.69)	1.19 (0.92-1.54)	
Q3; 6-8.5 h per day	438	36.9 (36.1-37.9)	487	31.3 (30.5-32.0)	1.06 (0.79-1.41)	1.16 (0.88-1.52)	
Q4; >8.5 h per day	527	36.3 (35.6-37.0)	431	30.3 (29.9-31.2)	1.18 (0.89-1.55)	1.46 (1.11-1.93)	
**% of time spent in Sedentary**							
Q1; <47%	462	37.5 (36.8-38.1)	805	31.9 (31.1-32.3)	1 (ref)	1 (ref)	p=0.033
Q2; 47%-54%	539	37.1 (36.6-37.7)	726	30.8 (30.2-31.4)	1.11 (0.83-1.48)	1.23 (0.97-1.54)	
Q3; 54%-61%	650	36.4 (35.8-37.0)	616	30.1 (29.5-30.7)	1.30 (0.99-1.71)	1.66 (1.31-2.09)	
Q4; >61%	803	34.8 (34.3-35.5)	464	27.9 (27.3-28.4)	1.97 (1.52-2.55)	3.01 (2.35-3.85)	
**% of time spent in prolonged Sedentary (of total wear time)**							
Q1; <13%	474	37.6 (37.0-38.2)	792	31.7 (30.8-32.2)	1 (ref)	1 (ref)	p=0.210
Q2; 13%-19%	536	37.2 (36.6-37.6)	731	31.1 (30.5-31.7)	1.24 (0.93-1.66)	1.08 (0.86-1.35)	
Q3; 19%-26%	673	36.1 (35.5-36.7)	592	30.1 (29.5-30.5)	1.55 (1.18-2.03)	1.64 (1.30-2.07)	
Q4; >26%	771	34.9 (34.4-35.5)	496	28.1 (27.7-28.6)	2.00 (1.54-2.60)	2.42 (1.90-3.08)	
**% of time spent in Light physical activity**							
Q1; <33%	840	35.4 (34.8-35.8)	427	28.6 (27.9-29.7)	1 (ref)	1 (ref)	p=0.124
Q2; 33%-40%	629	36.1 (35.5-36.7)	637	30.3 (29.8-31.0)	0.87 (0.70-1.09)	0.66 (0.51-0.85)	
Q3; 40%-46%	542	37.1 (36.7-37.8)	723	30.6 (30.0-31.2)	0.64 (0.50-0.81)	0.53 (0.41-0.69)	
Q4; >46%	443	37.0 (36.4-37.6)	824	31.0 (30.5-31.8)	0.67 (0.52-0.86)	0.46 (0.36-0.59)	
**% of time spent in Moderate physical activity**							
Q1; <3.8%	655	34.7 (34.1-35.3)	612	28.3 (27.7-28.9)	1 (ref)	1 (ref)	p=0.062

Q2; 3.8%-5.3%	581	36.4 (35.7-37.0)	684	30.1 (29.7-30.7)	0.61 (0.48-0.78)	0.61 (0.49-0.77)	
Q3; 5.3%-7.2%	594	37.0 (36.2-37.7)	672	31.0 (30.3-31.5)	0.56 (0.44-0.71)	0.48 (0.38-0.61)	
Q4; >7.2%	624	37.2 (36.7-37.9)	643	32.1 (31.3-32.5)	0.51 (0.40-0.65)	0.38 (0.30-0.48)	
**% of time spent in Vigorous physical activity**							
Q1;<0.02%	557	33.7 (33.3-34.2)	709	28.0 (27.4-28.5)	1 (ref)	1 (ref)	p=0.060
Q2; 0.02%-0.1%	566	34.9 (34.1-35.6)	700	28.7 (28.2-29.4)	0.79 (0.62-1.00)	0.82 (0.67-1.02)	
Q3; 0.1%-0.6%	640	37.1 (36.6-37.6)	627	31.4 (31.0-31.9)	0.37 (0.29-0.47)	0.31 (0.25-0.40)	
Q4; >0.6%	691	38.7 (38.1-39.4)	575	34.7 (33.9-35.3)	0.26 (0.20-0.33)	0.17 (0.13-0.23)	

[a] Adjusted for sex and age, comparing the lowest sex-specific tertile of estimated VO_2max with the two higher tertiles

[#] Sex-specific tertiles based on L/min. Analyses additionally adjusted for weight in kg.

Appendix Figure 1. Association between continous levels of estimated VO2max, BMI and waist circumference.

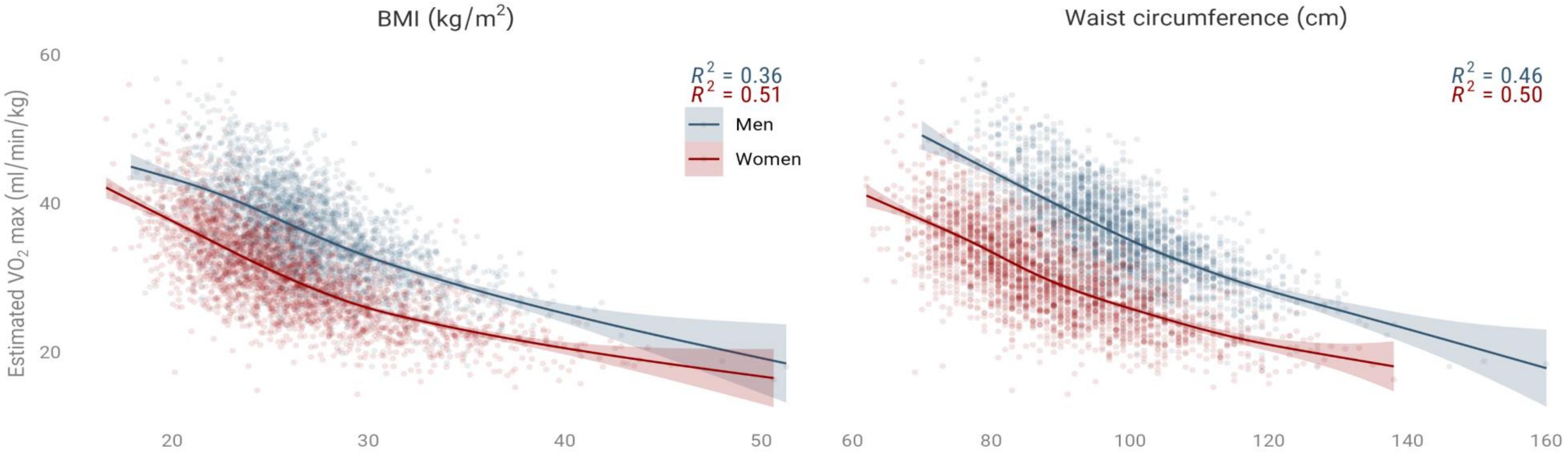

Appendix Figure 2. Association between estimated VO2max and continuous levels of accelerometer assessed sedentary time and physical activity of different intensities.

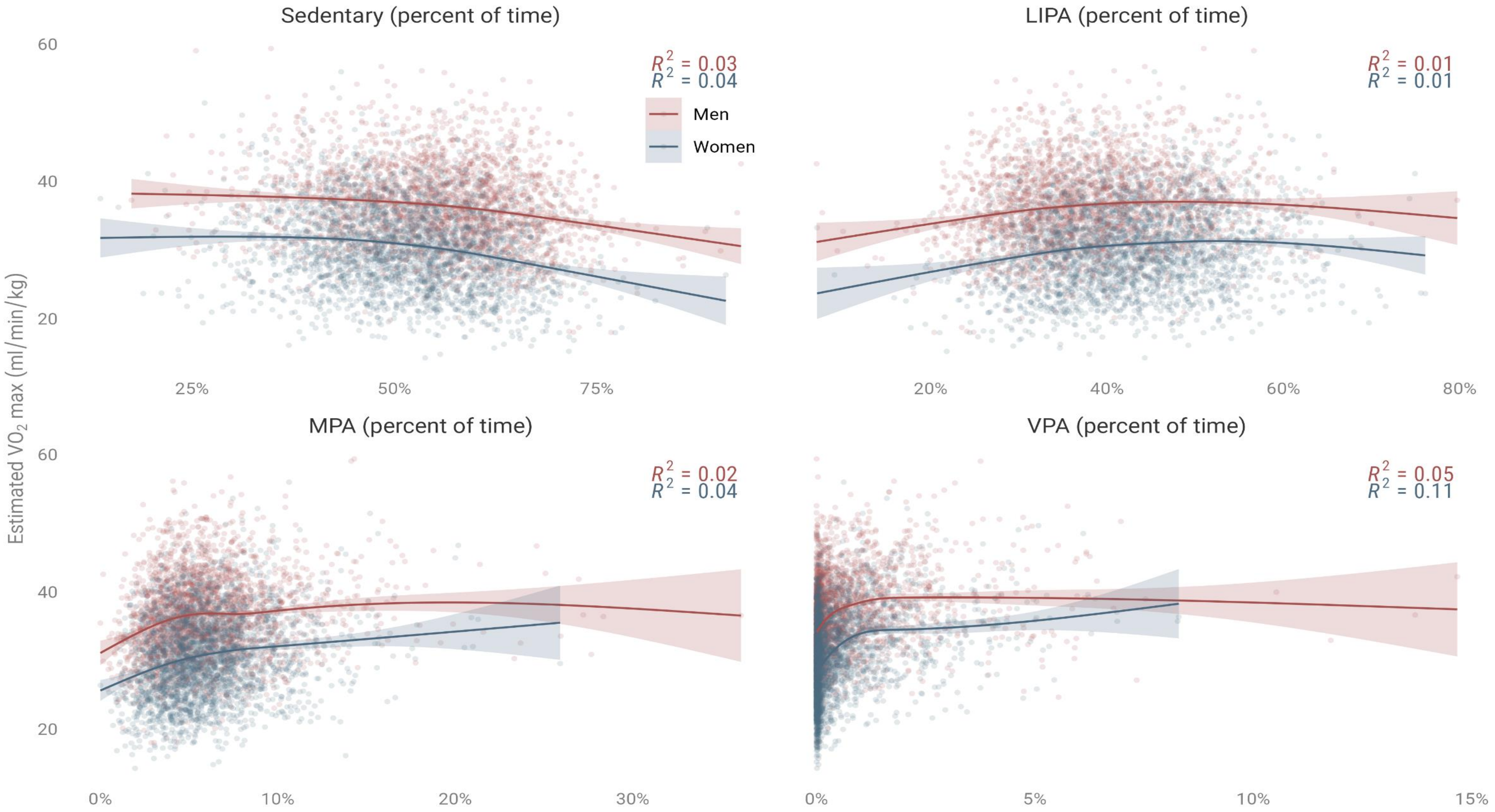

Supplement: Supplementary data [file bmjopen-2022-066336supp002.pdf]
