# Supplementary material for: Correlates of cardiorespiratory fitness in a population-based sample of middle-aged adults: cross-sectional analyses in the SCAPIS study
Source: BMJ Open. 2022 Dec 14;12(12):e066336. doi: 10.1136/bmjopen-2022-066336 (PMC9756301; doi:10.1136/bmjopen-2022-066336)
Supplement: Author's manuscript [file bmjopen-2022-066336.draft_revisions.pdf]

BMJ Open is committed to open peer review. As part of this commitment we make the peer review history of every article we publish publicly available.

When an article is published we post the peer reviewers' comments and the authors' responses online. We also post the versions of the paper that were used during peer review. These are the versions that the peer review comments apply to.

The versions of the paper that follow are the versions that were submitted during the peer review process. They are not the versions of record or the final published versions. They should not be cited or distributed as the published version of this manuscript.

BMJ Open is an open access journal and the full, final, typeset and author-corrected version of record of the manuscript is available on our site with no access controls, subscription charges or pay-per-view fees (http://bmjopen.bmj.com).

If you have any questions on BMJ Open's open peer review process please email info.bmjopen@bmj.com

BMJ Open

Correlates of cardiorespiratory fitness in middle-age – cross-sectional analyses in 5308 individuals in the SCAPIS study

Journal:	*BMJ Open*
Manuscript ID	bmjopen-2022-066336
Article Type:	Original research
Date Submitted by the Author:	04-Jul-2022
Complete List of Authors:	Börjesson, Mats; University of Gothenburg, Center for Health and Performance, Department of Molecular and Clinical Medicine,; Sahlgrenska University Hospital<br>Ekblom, Örjan; GIH, Department of Physical Activity and Health<br>Arvidsson, D; University of Gothenburg, Center for Health and Performance, Department of Food and Nutrition, and Sport Science<br>Heiland, Emerald G; GIH, Department of Physical Activity and Health<br>Väisänen, Daniel; GIH, Department of Physical Activity and Health<br>Bergström, Göran; Sahlgrenska University Hospital, Sahlgrenska Center for Cardiovascular and Metabolic Research, Wallenberg Laboratory; University of Gothenburg, Department of Molecular and Clinical Medicine<br>Ekblom-Bak, Elin; GIH, Department of Physical Activity and Health
Keywords:	EPIDEMIOLOGY, PREVENTIVE MEDICINE, PUBLIC HEALTH

SCHOLARONE™
Manuscripts

I, the Submitting Author has the right to grant and does grant on behalf of all authors of the Work (as defined in the below author licence), an exclusive licence and/or a non-exclusive licence for contributions from authors who are: i) UK Crown employees; ii) where BMJ has agreed a CC-BY licence shall apply, and/or iii) in accordance with the terms applicable for US Federal Government officers or employees acting as part of their official duties; on a worldwide, perpetual, irrevocable, royalty-free basis to BMJ Publishing Group Ltd ("BMJ") its licensees and where the relevant Journal is co-owned by BMJ to the co-owners of the Journal, to publish the Work in this journal and any other BMJ products and to exploit all rights, as set out in our licence.

The Submitting Author accepts and understands that any supply made under these terms is made by BMJ to the Submitting Author unless you are acting as an employee on behalf of your employer or a postgraduate student of an affiliated institution which is paying any applicable article publishing charge ("APC") for Open Access articles. Where the Submitting Author wishes to make the Work available on an Open Access basis (and intends to pay the relevant APC), the terms of reuse of such Open Access shall be governed by a Creative Commons licence – details of these licences and which Creative Commons licence will apply to this Work are set out in our licence referred to above.

Other than as permitted in any relevant BMJ Author's Self Archiving Policies, I confirm this Work has not been accepted for publication elsewhere, is not being considered for publication elsewhere and does not duplicate material already published. I confirm all authors consent to publication of this Work and authorise the granting of this licence.

For peer review only

TITLE PAGE

Title of the paper: Correlates of cardiorespiratory fitness in middle-age – cross-sectional analyses in 5308 individuals in the SCAPIS study

Authors: Mats Börjesson[1, 2], Örjan Ekblom[3], Daniel Arvidsson[4], Emerald G. Heiland[3, 5], Daniel Väisänen[3], Göran Bergström[6, 7], Elin Ekblom-Bak[3]

[1]Center for Health and Performance, Department of Molecular and Clinical Medicine, Sahlgrenska Academy, Gothenburg University, Sweden.

[2]Dept MGA, Sahlgrenska University Hospital, Region Västra Götaland, Gothenburg, Sweden

[3]Department of Physical Activity and Health, The Swedish School of Sport and Health Sciences, Stockholm, Sweden.

[4]Center for Health and Performance, Department of Food and Nutrition and Sport Science, Faculty of Education, University of Gothenburg.

[5]Department of Surgical Sciences, Medical Epidemiology, Uppsala University, Uppsala, Sweden.

[6]Department of Molecular and Clinical Medicine, Institute of Medicine, Sahlgrenska Academy, University of Gothenburg, Gothenburg, Sweden.

[7]Department of Clinical Physiology, Sahlgrenska University Hospital, Region Västra Götaland, Gothenburg, Sweden.

Corresponding author: Elin Ekblom-Bak, The Swedish School of Sport and Health Sciences, Department of Physical Activity and Health, Stockholm, Sweden. Box 5626, S-114 56 Stockholm, Sweden. Phone: +46812053861. Email: eline@gih.se

For peer review only - http://bmjopen.bmj.com/site/about/guidelines.xhtml

ABSTRACT

Objectives: The study aimed to identify main sex-specific correlates of cardiorespiratory fitness (CRF) in a population-based, urban sample of 50–64-year-old Swedish men and women.

Design: Cross-sectional.

Setting: Multi-site study at university hospitals

Participants: A total of 5,308 participants (51% women, aged 50–64 years) with a valid estimated VO_2max from submaximal cycle testing from the Swedish CArdioPulmonary bioImage study, SCAPIS, were included.

Primary and secondary outcomes: A wide range of correlates were examined including a) sociodemographic and lifestyle behaviors b) perceived health, anthropometrics, and chronic conditions, and c) self-reported as well as accelerometer-derived PA and sedentary behaviors. Both continuous levels of estimated VO_2max as well as risk (odds ratio) of low VO_2max (lowest sex-specific tertile) were reported.

Results: Higher age, born abroad, having a short education, high waist circumference, being a daily smoker, having poor perceived health, high accelerometer-derived time in sedentary and low in VPA, as well as being passive commuter correlated independently with low estimated VO_2max in both men and women. Specifically in men, financial strain and being an ex-smoker associated with higher risk, and constant stress with lower risk, for low VO_2max. Specifically in women, being a regular smoker associated with lower risk of low VO_2max.

Conclusions: The present study provides important reference material of a general middle-aged population for future research, clinical practice, and public health work. If relations are causal, increased knowledge about specific sub-groups will aid in the development of appropriate, targeted interventions.

For peer review only - http://bmjopen.bmj.com/site/about/guidelines.xhtml

Strengths and limitations of this study

- The large sample size with estimated CRF from a submaximal test and accelerometer-measured PA provided the power required to analyse multiple correlates relevant for CRF.
- However, the cross-sectional design of the study prevents any causal inference and we cannot rule out reverse causality.
- We have no information on any genetic contribution to variation between sub-groups of correlates.
- Although we have a large sample from the general population, those who participated in the fitness testing did differ from the source population. Therefore, we need to cautious in generalizing to populations that are less healthy and active, and of other socioeconomic status.

Keywords Cardiorespiratory fitness, VO_2max, population based, correlates, SCAPIS, accelerometer-derived physical activity, reference material

WORD COUNT: 3302 words

For peer review only

BACKGROUND

Cardiorespiratory fitness (CRF) assessed as maximal oxygen consumption (VO_2max) is as a strong, independent predictor for several non-communicable diseases and premature mortality (1, 2) as well as for aerobic performance and engagement in daily and exercise activities. Unfortunately, data indicate a decline in CRF on population levels over the last decades (3, 4).

CRF level is mainly determined by recent moderate-to-vigorous physical activity (MVPA) performed with also a strong genetic component (25-50% reported) (5). CRF is typically lower in older ages (6) and in individuals with shorter education (7), with negative association with smoking and obesity, and positive association with leisure time PA or exercise (8). However, a broader knowledge of key determinants for CRF level is scarce, especially from population-based samples. For example, whether and how CRF levels vary with psychosocial factors and type of domain-specific PA behavior are not fully elucidated or show conflicting results (8, 9). Moreover, previous studies of PA correlates on CRF are often limited by the use of self-reported PA (10). Also, it is still not fully explored whether and how the age-related lower levels of CRF are associated with less exercise performed, or instead is due to age-related changes in key physiological capacities itself (11). Previous studies have mainly been based on selected samples, consisting of smaller cohorts (n<2000), which limits the power for comparisons between different sub-groups (8). More detailed knowledge of current correlates in unselected samples is essential to understand variations of CRF in the population, which is needed not least for the development of appropriate, targeted interventions.

The aim of the present study is to identify main sex-specific correlates of CRF in a population-based, urban sample of 50–64-year-old men and women, based on a wide range of correlates including a) sociodemographic and lifestyle behaviors b) perceived health, anthropometrics, and chronic conditions, and c) self-reported as well as accelerometer-derived PA and sedentary behaviors. A secondary aim is to study whether the commonly reported

For peer review only - http://bmjopen.bmj.com/site/about/guidelines.xhtml

lower CRF with older age might be influenced by level of accelerometer-derived sedentary time and/or vigorous PA.

METHODS

Data was retrieved from The Swedish CArdioPulmonary bioImage Study (SCAPIS) (12). In 2012, a comprehensive pilot study was conducted and in 2013-2018 the full SCAPIS study was carried out in collaboration with six Swedish university hospitals (Gothenburg, Linköping, Malmö/Lund, Stockholm, Umeå, and Uppsala), aiming to recruit 5000 individuals (men and women, 50-64 years) from their respective municipality. Data collection took place over two to three occasions within a 2-week period. The participants underwent extensive imaging and functional studies of the heart, lungs, and metabolism, filled in an extensive questionnaire and wore an accelerometer during seven days. At each test-site, there was a possibility to include site-specific measurements. At the Gothenburg site, a submaximal cycle test was performed to estimate VO_2max. In the pilot study, a total of 2,243 participants were recruited from the census register, and 1,111 (49.5%) agreed to participate. In the full study in Gothenburg, 12,109 were recruited and 6,266 (52%) participated. Thus, a total of 7,377 were eligible and 5,308 participants (72%) provided valid estimated VO_2max. The study was approved by the ethics board (Dnr 2010-228-31M, Dnr 2011-449-32M and Dnr 638-16) and adheres to the Declaration of Helsinki. All participants provided written informed consent.

For peer review only

Assessment of cardiorespiratory fitness

VO_2max was estimated using Ekblom-Bak submaximal cycle ergometer test (13). The test has shown high validity in reference to direct measurement of VO_2max in this age-group (r^2=0.84, standard error of estimate 0.33 L□min^{-1}) (13). To minimize well-known errors with submaximal testing, participants were requested to refrain from consuming a heavy meal or

For peer review only - http://bmjopen.bmj.com/site/about/guidelines.xhtml

drinking coffee during the hours before the test. A priori exclusion criteria included symptoms of on-going infections, known cardiovascular disease, electrocardiography patterns indicative of cardiac disease, use of beta-blockers, weight>125 kg or resting heart rate of >100 bpm (n=466). Moreover, participants did not take part in the testing if refrained from participating in testing (n=1209), or did not receive a valid fitness test due to lack of test criteria fulfilment, did not fulfil the test, or malfunction of heart rate monitors or the ergometer (n=394).

Assessment of anthropometrics and chronic conditions

Measurements of weight, height, and waist circumference were assessed during the first visit to the test centre. Body mass index (BMI) was calculated (kg□m^{-2}), and waist circumference was measured at the midpoint between the top of the iliac crest and the lower margin of the lowest palpable rib. Weight at 20 years of age, as well as prevalent depressive symptoms lasting 2 weeks or more during the last 12 months were self-reported. Prevalent chronic conditions were defined as reporting diagnosed disease or surgical treatment for cardiovascular disease (myocardial infarction, angina pectoris, stroke, congestive heart failure, atrial fibrillation), hypertension, lung disease, dyslipidaemia, diabetes, rheumatic disease, and/or cancer.

Assessment of physical activity pattern

Self-reported PA included commuting habits, physical working situation, exercise, leisure time sitting, and total PA (see Additional File 1). Sedentary behavior and PA were also derived from tri-axial accelerometers (ActiGraph model GT3X and GT3X+, ActiGraph LCC, Pensacola, FL, USA). Participants were instructed to wear the accelerometer on an elastic belt over the right hip during all waking hours for at least 7 consecutive days, except during water-based activities. ActiLife v.6.10.1 software was used to initialize the accelerometers and to

For peer review only

For peer review only - http://bmjopen.bmj.com/site/about/guidelines.xhtml

download and process the collected data. The accelerometer recorded raw data (30 Hz) from all three axes, which were combined into a resulting vector, and extracted as 60 second epochs using a low frequency extension filter. Sedentary was defined as <200 counts per minute (cpm) (14), low intensity PA as 200-2689 cpm, moderate intensity PA as 2690-6166 cpm, and vigorous PA (VPA) as ≥6167 cpm (15). Non-wear time was defined as ≥60 consecutive minutes with no movement (0 cpm), with allowance for maximum 2 minutes of counts between 0 and 200 cpm. Wear time was calculated as 24 hours minus non-wear time. A minimum of 600 minutes of valid daily wear time for at least 4 days was required for inclusion (16). Prolonged sedentary was defined as ≥20 minutes of cpm below 200, with no allowance for interruption above threshold.

Assessment of sociodemographic, lifestyle, and perceived symptoms and health

Marital status, country of birth, educational level, employment status, financial strain, and smoking status were self-reported and grouped according to definitions presented in Table 1. Pack-years of smoking was derived by multiplying the average number of cigarettes smoked per day by the number of years smoking, divided by the number of cigarettes per package. The 10-item screening tool AUDIT was used to define alcohol abuse (17), and categorized into three groups "No/Low", "Moderate", "High", according to the AUDIT score 0-7, 8-15, and >15 for men, and 0-5, 6-13, and >13 for women. Sleep, stress, perceived control at work and in leisure-time, as well as general health, were also self-reported and grouped as presented in Table 2.

Patient and public involvement

No patient involved.

For peer review only - http://bmjopen.bmj.com/site/about/guidelines.xhtml

Statistical analysis

CRF was skewed (Kolmogorov-Smirnov Test), hence, descriptive data are presented as medians and 95% confidence intervals (CIs). Low CRF was defined as the lowest sex-specific tertile (cut-off 27.75 mL·min^{-1}·kg^{-1} for women and 33.65 mL·min^{-1}·kg^{-1} for men). Odds ratios (ORs) with 95% CIs for low CRF in relation to the sub-groups of the correlates were calculated using binary logistic regression modeling adjusting for sex and age. Interaction between sex and each correlate was assessed by adding an interaction term to the model, and significant interaction(s) were defined as p<0.05 for the interaction term. To study independent associations of correlates with low CRF, all correlates not displaying multicollinearity (Spearman's ρ>0.6) were entered in a backward logistic multivariable regression model, with low CRF as dependent variable. To study whether the relationship between age-group and estimated VO_2max was modified by VPA or prolonged sedentary, general linear modeling with post-hoc analyses was used including an interaction term for age-group*VPA or age-group*prolonged sedentary and adjusting for sex. The VPA variable was divided into four groups; 0 min/week, 0 to 37.5 min/week (half of the recommended VPA level), >37.5 to <75 min/week (the recommended VPA level), and ≥ 75 min/day (18). For sedentary behavior, the quartiles from Table 3 were used. Statistical analyses were performed using SPSS (26.0, 2019, SPSS Inc., Chicago IL) and R (R version 4.1.1) with R packages ggplot2 and ggeffects.

RESULTS

Of the total of 7,377 participants eligible for exercise testing in the study, 5,308 provided valid estimates for VO_2max. Participants included, compared with participants not included (n=2,069), were more often women (54% vs 51%, p=0.040), younger (age 57.2 vs 58.2 years, p<0.001), had lower BMI (26.5 vs 27.7 kg·m^{-2}, p<0.001), had more often university degree

For peer review only

For peer review only - http://bmjopen.bmj.com/site/about/guidelines.xhtml

(47% vs 35%, p<0.001), and more often reported to be weekly regular exercisers (53% vs 40%, p<0.001).

Associations between CRF and the different correlates are presented in Tables 1 to 3 and Appendix Figure 1 and 2, with sex-specific analyses presented in Appendix Table 1. The results can be summarized as:

- Sociodemographics and Lifestyle behaviors (Table 1): Women, older age, divorced/single/widowed, participants not born in Sweden, having lower education, employment<50% of full-time/retired/pension/unemployed, financial strain, or being regular and former smokers had lower estimated CRF and higher OR for having low CRF, compared with their counterparts. Older men (60-64 years) were more likely to have lower CRF than older women (p=0.045), with no other interactions seen between sex and other sociodemographic variables. For lifestyle factors there were significant interactions between sex and smoking variables, such that men being regular smokers/sometimes smokers, and with more pack-years in life, were more likely to have low CRF (p=0.004 and p=0.008) compared with smoking women.

For peer review only

Table 1. Estimated VO_2max (ml/min/kg) in association with sociodemographics and lifestyle behavior correlates.

		**Estimated VO_2max**	**Low estimated VO_2max**	
	n	Median (95% CI)	% within subgroup	OR^C (95% CI)
**Sex**				
Men	2590	36.2 (35.8-36.5)[a]	na[d]	na
Women	2718	30.4 (30.1-30.7)	na	na
**Age**				
50-54 years	1881	34.9 (34.5-35.1)[b]	24%	1 (ref)
55-59 years	1812	33.7 (33.2-34.0)	32%	1.46 (1.27-1.69)
60-64 years	1615	31.5 (31.0-31.8)	45%	2.57 (2.23-2.97)

For peer review only - http://bmjopen.bmj.com/site/about/guidelines.xhtml

**Marital status**				
Married/Cohabitation	3766	33.8 (33.6-34.1)[a]	31%	1 (ref)
Divorced/Single/Widower	1461	32.1 (31.7-32.6)	38%	1.37 (1.21-1.56)
**Born in Sweden**				
Yes	4341	33.7 (33.4-33.9)[a]	31%	1 (ref)
No	923	32.0 (31.3-32.5)	43%	1.75 (1.51-2.03)
**Educational level**				
University degree	2480	34.3 (33.9-34.6)[b]	26%	1 (ref)
High school/Vocational education	2215	32.8 (32.4-33.2)	38%	1.85 (1.63-2.10)
Elementary school	555	31.6 (30.8-32.4)	50%	2.82 (2.32-3.42)
**Employment**				
Working, ≥ 50% of full-time	4342	33.9 (33.6-34.1)[b]	30%	1 (ref)
Retired	231	30.3 (29.8-31.4)	53%	1.55 (1.17-2.05)
Disability pension/Sickness pension/Sick leave	299	29.3 (28.4-30.2)	54%	2.60 (2.05-3.31)
Unemployed/Student	365	31.3 (30.9-32.2)	45%	1.88 (1.51-2.35)
**Financial strain**				
No	4759	33.6 (33.4-33.9)[a]	32%	1 (ref)
Yes	406	30.1 (29.6-30.7)	51%	2.51 (2.04-3.10)
**Smoking habits**				
Never smoker	2463	34.6 (34.2-34.9)[b]	29%	1 (ref)
Former smokers	2015	32.3 (31.9-32.6)	38%	1.38 (1.21-1.57)
Regular smoker/Sometimes	716	32.2 (31.8-32.9)	39%	1.61 (1.35-1.92)
**Pack-years**				
Never smokers (0)	2463	34.6 (34.2-34.9)[b]	29%	1 (ref)
Former smokers, low (<15 pack-years)	1220	33.0 (32.6-33.7)	30%	1.00 (0.86-1.17)
Former smokers, heavy (≥15 pack-years)	795	31.1 (30.5-31.6)	49%	2.17 (1.83-2.56)
Current smokers, low (<15 pack-years)	226	33.6 (32.4-34.8)	30%	1.11 (0.82-1.50)
Current smokers, heavy (≥15 pack-years)	490	31.8 (31.1-32.3)	42%	1.88 (1.53-2.30)
**Alcohol abuse**				
No/Low	3948	33.7 (33.4-34.0)	30%	1 (ref)
Moderate	639	33.3 (33.0-33.9)	34%	1.26 (1.05-1.51)
High	33	32.5 (30.0-33.9)	52%	3.06 (1.51-6.19)

[a] Significantly different from other sub-group (Mann–Whitney U test, p<0.0001)

[b] Kruska-Wallis ANOVA p<0.0001

[c] Adjusted for sex and age, comparing the lowest sex-specific tertile of estimated VO_2max with the two higher tertiles

[d] % in low estimated VO_2max and OR not applicable for comparison between men and women, as tertiles were sex-specific.

For peer review only

- Perceived health, anthropometrics, and chronic conditions (Table 2 and Appendix Figure 1): Participants reporting poor sleep, high stress, low control at work, low perceived degree of control in life, with poorer general health, higher BMI, higher waist circumference, larger weight gain compared with weight at 20 years of age, prevalence of depression symptoms, and higher number of chronic conditions had lower estimated CRF. Women with low perceived degree of control in life were more likely to have low CRF than their male counterparts ($p=0.004$). Higher BMI, higher waist circumference, and greater weight gain since the age of 20, were more strongly associated with low CRF in women than in men ($p=0.001$, $p=0.041$ and $p<0.001$).

Table 2. Estimated VO_2max (ml/min/kg) in association with perceived health, anthropometrics and chronic condition correlates.

		**Estimated VO_2max**	**Low estimated VO_2max**	
	n	Median (95% CI)	% within subgroup	OR[C] (95% CI)
**Sleep**				
Very well/well	2401	34.2 (33.9-34.5)[a]	31%	1 (ref)
Rather well/Badly/Very badly	2835	32.6 (32.2-32.9)	35%	1.22 (1.09-1.38)
**Stress**				
No stress/Some stress during last 5 years	4089	33.7 (33.4-33.9)[a]	33%	1 (ref)
Constant stress last 1 to 5 years	1129	32.2 (31.8-32.6)	36%	1.25 (1.08-1.44)
**Control at work**				
Strongly agree/Agree	3846	33.8 (33.6-34.1)[a]	31%	1 (ref)
Neutral/Do not agree/Do not agree at all	1111	32.3 (31.9-32.8)	35%	1.23 (1.06-1.42)
**Control in life**				
Do not agree at all/Do not agree	2976	34.0 (33.7-34.3)[a]	30%	1 (ref)
Neutral/Agree/Strongly agree	2193	32.5 (32.1-32.8)	37%	1.46 (1.29-1.64)
**General health**				
Excellent/Very good	2698	35.0 (34.8-35.3)[b]	23%	1 (ref)
Good	1818	32.5 (32.2-33.0)	40%	2.41 (2.11-2.75)
Somewhat bad/Bad	729	29.4 (28.8-30.1)	56%	4.80 (4.02-5.73)
**BMI ($kg\cdot m^{-2}$) with estVO_2max in $ml\cdot min^{-1}\cdot kg^{-1}$**				

For peer review only

For peer review only - http://bmjopen.bmj.com/site/about/guidelines.xhtml

<18	11	42.1 (40.8-45.6)[b]	0%	-
18.0-24.9	2025	36.4 (36.1-36.7)	10%	1 (ref)
25.0-29.9	2341	32.9 (32.5-33.2)	36%	6.19 (5.17-7.41)
30.0-34.9	728	28.2 (27.8-28.9)	74%	38.3 (30.2-48.7)
≥35	203	23.0 (22.5-23.8)	98%	659.0 (241.0-1805.1)
**BMI with estVO_2max in L□min^{-1}**				
<18	11	2.09 (1.79-2.55)[b]	55%	0.65 (0.19-2.29)
18.0-24.9	2025	2.38 (2.35-2.40)	36%	1 (ref)[#]
25.0-29.9	2341	2.75 (2.70-2.80)	33%	2.32 (1.95-2.76)
30.0-34.9	728	2.63 (2.52-2.73)	29%	5.79 (4.30-7.81)
≥35	203	2.38 (2.29-2.49)	29%	16.6 (10.2-27.1)
**Waist circumference**				
Low waist (<88 cm women, <102 cm men)	3191	36.4 (36.2-36.7)[a]	15%	1 (ref)
High waist (≥88 cm women, ≥102cm men)	2114	28.9 (28.6-29.2)	61%	10.2 (8.8-11.7)
**Current weight compared to weight at 20 years of age**				
Less now compared to 20 years of age	269	39.0 (37.6-39.8)[b]	10%	1 (ref)
0 to 10 kg more now compared at 20 years of age	1739	36.8 (36.4-37.1)	11%	1.13 (0.74-1.73)
10 to 20 kg more now than at 20 years of age	1787	33.0 (32.7-33.3)	32%	4.28 (2.84-6.45)
>20 kg more now than at 20 years of age	1231	28.0 (27.6-28.4)	71%	24.2 (15.9-36.7)
**Depression symptoms**				
No	3687	33.9 (33.7-34.2)[a]	32%	1 (ref)
Yes	1495	32.0 (31.6-32.3)	37%	1.33 (1.17-1.51)
**Chronic conditions***				
0	3465	33.9 (33.7-34.3)[b]	30%	1 (ref)
1-2	1705	32.3 (31.9-32.8)	39%	1.38 (1.22-1.57)
=>3	138	31.0 (29.7-32.7)	51%	2.09 (1.48-2.96)
**Cardiovascular disease, Hypertension and/or Lung disease**				
No	4069	33.7 (33.5-34.0)	32%	1 (ref)
Yes	1239	32.0 (31.5-32.5)	37%	1.42 (1.24-1.62)

[a] Significantly different from other sub-group (Mann–Whitney U test, p<0.0001)

[b] Kruska-Wallis ANOVA p<0.0001

[c] Adjusted for sex and age, comparing the lowest sex-specific tertile of estimated VO_2max with the two higher tertiles
* Includes Cardiovascular disease, Hypertension, Lung disease, Dyslipidaemia, Diabetes, Rheumatic disease and Cancer

[#] Sex-specific tertiles based on L/min. Analyses additionally adjusted for weight in kg.

For peer review only

For peer review only - http://bmjopen.bmj.com/site/about/guidelines.xhtml

- Self-reported and accelerometer-derived PA pattern (Table 3 and Appendix Figure 2): Participants engaging in any active commuting had higher CRF compared with those who were not active commuters. This was mainly driven by higher CRF among partly- and all year commuters by bike. Participants with a more strenuous physical working situation, less weekly exercise, lower self-reported total PA level, and high self-reported sitting time had lower CRF. Women reporting higher total PA had lower odds for low CRF (p=0.021) compared with men. Higher accelerometer-derived time spent in sedentary and prolonged sedentary time were associated with lower CRF, whereas more time spent in low intensity PA, moderate PA, and VPA were associated with higher CRF. Women with higher sedentary time were more likely to have lower CRF compared with men (p=0.033).

Table 3. Estimated VO_2max (ml/min/kg) in association with self-reported and accelerometer-derived physical activity pattern correlates.

		**Estimated VO_2max**	**Low estimated VO_2max**	
	n	Median (95% CI)	% within subgroup	OR[C] (95% CI)
**Self-report**				
**Commuting habits***				
No active commuting	3057	33.1 (32.8-33.4)[a, b]	37%	1 (ref)
Any active commuting, further divided into:	1738	34.6 (34.2-35.0)	23%	0.47 (0.41-0.54)
Partly active commuter, bike	671	34.8 (34.1-35.5)	20%	0.41 (0.33-0.50)
Partly active commuter, walking	196	30.4 (29.3-31.6)	41%	1.12 (0.83-1.52)
Partly active commuter, mix	19	30.2 (28.8-33.5)	21%	0.45 (0.15-1.38)
All year active commuter, bike	407	38.1 (36.9-39.2)	10%	0.18 (0.13-0.25)
All year active commuter, walking	415	33.0 (32.2-33.4)	31%	0.71 (0.57-0.89)
All year active commuter, mix	30	34.7 (30.6-37.3)	23%	0.51 (0.22-1.22)
**Physical working situation**				
Sedentary to light	3921	33.7 (33.5-34.1)[a]	30%	1 (ref)
Sometimes to frequently heavy	945	32.5 (32.0-33.1)	38%	1.44 (1.24-1.67)
**Exercise habits**				
Never	1384	31.0 (30.7-31.4)[b]	51%	1 (ref)
Irregular	1067	31.3 (30.8-31.7)	43%	0.75 (0.64-0.88)
1-2 times per week	1032	33.5 (33.0-34.1)	27%	0.35 (0.29-0.41)
2-3 times per week	1089	35.2 (34.8-35.7)	20%	0.25 (0.21-0.30)
>3 times per week	649	38.3 (37.8-38.9)	12%	0.14 (0.11-0.19)

For peer review only - http://bmjopen.bmj.com/site/about/guidelines.xhtml

**Total physical activity**				
Sedentary	540	30.0 (29.1-30.5)[b]	63%	1 (ref)
Light	2366	31.5 (31.2-31.9)	42%	0.39 (0.32-0.48)
Moderate	1613	34.9 (34.6-35.4)	21%	0.14 (0.11-0.18)
Regular exercise/training	669	38.6 (38.0-39.1)	9%	0.06 (0.05-0.09)
**Leisure time sitting**				
Q1; <4 h per day	1121	34.1 (33.5-34.7)	28%	1 (ref)
Q2; 4-6 h per day	1039	33.6 (33.0-34.2)	32%	1.24 (1.02-1.49)
Q3; 6-8.5 h per day	925	34.0 (33.5-34.5)	29%	1.11 (0.91-1.36)
Q4; >8.5 h per day	958	33.7 (33.3-34.2)	32%	1.31 (1.08-1.59)
**% of time spent in Sedentary**				
Q1; <47%	1267	34.1 (33.5-34.5)[b]	26%	1 (ref)
Q2; 47%-54%	1265	33.6 (33.1-34.1)	28%	1.19 (0.99-1.42)
Q3; 54%-61%	1266	33.4 (33.0-33.7)	33%	1.52 (1.27-1.81)
Q4; >61%	1267	33.0 (32.3-33.3)	43%	2.44 (2.05-2.91)
**% of time spent in prolonged Sedentary (of total wear time)**				
Q1; <13%	1266	34.1 (33.6-34.5)[b]	25%	1 (ref)
Q2; 13%-19%	1267	33.5 (33.0-34.0)	28%	1.14 (0.96-1.37)
Q3; 19%-26%	1265	33.5 (33.1-34.0)	35%	1.60 (1.35-1.91)
Q4; >26%	1267	32.7 (32.2-33.1)	42%	2.18 (1.83-2.60)
**% of time spent in Light physical activity**				
Q1; <33%	1267	33.7 (33.2-34.0)	39%	1 (ref)
Q2; 33%-40%	1266	33.3 (32.9-33.7)	34%	0.78 (0.66-0.92)
Q3; 40%-46%	1265	33.5 (33.1-34.0)	29%	0.60 (0.51-0.72)
Q4; >46%	1267	33.2 (32.8-33.8)	28%	0.56 (0.47-0.66)
**% of time spent in Moderate physical activity**				
Q1; <3.8%	1267	31.6 (31.6-32.5)[b]	44%	1 (ref)
Q2; 3.8%-5.3%	1265	33.3 (32.7-33.7)	32%	0.62 (0.52-0.73)
Q3; 5.3%-7.2%	1266	33.8 (33.3-34.1)	29%	0.52 (0.44-0.62)
Q4; >7.2%	1267	34.9 (34.3-35.3)	26%	0.44 (0.37-0.52)
**% of time spent in Vigorous physical activity**				
Q1;<0.02%	1266	30.5 (30.1-31.0)[b]	49%	1 (ref)
Q2; 0.02%-0.1%	1266	31.7 (31.3-32.1)	43%	0.80 (0.69-0.94)
Q3; 0.1%-0.6%	1267	34.3 (34.0-34.7)	23%	0.34 (0.28-0.40)
Q4; >0.6%	1266	36.7 (36.3-37.1)	16%	0.21 (0.18-0.26)

* Analysed both as comparing No active commuting and Any active commuting, as well as further dividing any active commuting into; Partly commuting, defined as going by bike or walking at least one season out of four; All year commuting, defined as going by bike or walking all year around. Partly and all year commuting "walking" or "bike" were defined as the

For peer review only - http://bmjopen.bmj.com/site/about/guidelines.xhtml

For peer review only

majority of seasons commuted with that mode of active commuting. Mix was defined when the seasons of active commuting were spent equally between the two modes.

In the multivariable regression model (Figure 1), older age-group, being born abroad, having a lower educational attainment, high waist circumference, poorer general health, and more time spent sedentary, were associated with a higher OR for low CRF in both men and women. More time spent in VPA and commuting by bike (partly and all-year) were associated with a lower OR. Specifically in men, financial strain and being an ex-smoker were associated with higher OR, and constant stress with a lower OR, for a low CRF. Specifically in women, being a regular smoker was associated with lower OR for low CRF, with no significant associations with financial strain or stress.

Insert **Figure 1** here.

In left part of Figure 2, higher levels of VPA were more common in younger age-groups. However, higher levels of VPA associated with significantly higher CRF in all age-groups ($p<0.001$). There was no overall interaction between VPA and age-groups ($p=0.372$). Stratified analyses showed that there was no significant difference in CRF between participants with 37.5-75 minutes and ≥75 minutes of VPA per week in the oldest age-group ($p=1.00$). Also, participants aged 50-54 years and 55-59 years with 0 min/week of VPA had similar CRF ($p=0.305$ for differences between groups), and so did participants aged 55-59 and 60-64 years with 37.5 to 75 min/week ($p=1.000$ for differences between groups). In right part of Figure 2, higher levels of time spent in sedentary was associated with lower CRF in all age-groups. There was no overall interaction effect between sedentary and age-group ($p=0.596$). Stratified analyses showed that there was some overlapping between age-groups and time sedentary for CRF.

For peer review only - http://bmjopen.bmj.com/site/about/guidelines.xhtml

Insert **Figure 2** here.

DISCUSSION

The present study identified multiple correlates of CRF in a large, randomly selected population of middle-aged urban Swedish adults from the SCAPIS study. The results confirm some previously documented correlates, but also adds important novel aspects, including correlates of depression symptoms, commuting habits, and accelerometer-derived PA. Some of the most interesting correlates are discussed, as follows:

Firstly, the strong, positive association between CRF and length of education confirms previous results (7-9), while similar associations with being born in Sweden, being employed and having an economic buffer, adds novel information regarding sociodemographic aspects. These associations may largely be mediated by level of VPA, as we previously have shown that individuals in these sub-groups engage in higher levels of daily VPA compared with their counterparts (19), and a strong agreement between socioeconomic factors and both PA and CRF (7). The association between lower CRF in groups with lower socioeconomic status is also in line with higher observed incidence of cardiovascular, mood-related disorders and premature mortality in lower socioeconomic strata compared with those of higher socioeconomic status (20). This is further strengthen in a recent paper on risk of severe COVID-19, where the associations between socioeconomic factors as educational level, income, and occupational group and severe COVID-19 risk, were shown to be mediated to a large extent by the CRF level of the participants (21).

Secondly, participants reporting more physically demanding work situations had in general a lower CRF, compared with more sedentary workers. While this is in line with previous

For peer review only - http://bmjopen.bmj.com/site/about/guidelines.xhtml

reports from the Swedish working population (22), this result may at first be counterintuitive, as higher occupational PA could be considered as a "work-out" contributing to health benefits and higher CRF. However, although more physically demanding occupations induce a higher mean intensity compared with more sedentary occupations, the relative intensity of physically demanding occupations have been shown to be maybe too low to improve VO_2max (23). Rather, the higher mean intensity may be tiring enough for the individual, which could lead to lower levels of fitness-enhancing leisure time VPA (19). This, together with a more pronounced increase in body weight in blue-collar (more active) occupational groups (24), may contribute to the general lower CRF levels in these groups. Also, the mean intensity during work has been associated with lower heart rate variability and a higher heart rate the following night (23). This suggests an induced imbalance in the autonomic cardiac modulation, which may be one potential underlying mechanism for the detrimental effects on health proposed for occupational PA, also called the "physical activity paradox" (25). Thus, differences in CRF may partly contribute to the observed paradox.

Thirdly, we could confirm the established relation between self-reported total and leisure-time PA and CRF (8), however, expanding on knowledge regarding CRF in active commuters. Any active commuting was associated with higher CRF, however, the higher CRF was mainly driven by partly and all-year commuters by bike. This is line with higher levels of VPA shown in active commuters (19). Although the present study cannot clarify whether it was the active commuting per se that contributed to higher CRF levels, mean intensity during normal commuting in habitual cycle commuters has been shown to be mainly constituted of the lower end of vigorous intensity (26) for longer activity bouts (>10 minutes), often adding up to recommended levels of 150 minutes per week (27). Thus, regular active commuting by bike requires an at least moderate or even high CRF.

For peer review only - http://bmjopen.bmj.com/site/about/guidelines.xhtml

Fourthly, previous data on correlates between CRF and accelerometer-assessed PA patterns are scarce and inconclusive (8). We found that higher accelerometer-derived estimates of the different components of the PA pattern provided a strong association between CRF level and time in sedentary (negative association), and MPA and VPA (positive associations). Interestingly, both time in sedentary and in VPA remained significantly associated with CRF in the multivariable regression model, which implies separate importance of these intensities for CRF level.

Finally, previous research is unclear about whether higher PA level would ameliorate lower values of CRF among older individuals (8). The cross-sectional analyses in our study showed that older individuals had lower CRF than younger, with strong associations between higher VPA and less time spent sedentary, and CRF in all age-groups. More time in VPA may be required for older individuals in order to maintain a certain CRF, as the mean estimated VO_2max in individuals 60-64 years old engaging in 37 minutes or more in VPA per week was overlapping with the VO_2max in individuals 50-54 years old doing less than 37 minutes of VPA per week. It is also possible that relations are reversed or at least bi-directional, i.e., that a higher CRF will enable an individual to be physically active, including higher intensity activities. Indeed, previous studies have shown that those being active at higher age, have superior CRF compared with less active peers (11). Age-related medico-physiologic changes, possibly affecting the possibility to be active, include loss of muscle mass or increasing concomitant disease and medications, may affect the intensity of the VPA performed (6).

Strengths and limitations

For peer review only - http://bmjopen.bmj.com/site/about/guidelines.xhtml

The cross-sectional design of the study prevents any causal inference and we cannot rule out reverse causality. We have no information on any genetic contribution to variation between sub-groups of correlates. Although we have a large sample from the general population, those who participated in the fitness testing did differ from the source population. Therefore, we need to cautious in generalizing to populations that are less healthy and active, and of other socioeconomic status. Replication studies should be performed to confirm our results. Further, the large sample size with estimated CRF from a submaximal test and accelerometer-measured PA provided the power required to analyse multiple correlates relevant for CRF. It is unknown if regular bicycling affects validity of a cycle ergometer test. Local adaptations to the thigh and gluteal muscles from regular cycling may alter systemic circulatory response to standard cycling exercise. If so, the finding that regular bike commuters had higher CRF may in part be ascribed to varying validity.

CONCLUSIONS

This study in a large sample of middle-aged men and women delineates multiple modifiable as well as non-modifiable correlates of CRF, identifying specific sub-groups of the correlates as potential high-risk groups for CRF-related disorders which constitute possible targets for behavioral change to increase CRF, if relations are causal. These groups include individuals with higher age, born abroad, having a short education, high waist circumference, being a daily smoker, having poor perceived health, high accelerometer-derived time in sedentary and low in VPA, as well as passive commuters. Across all age-groups, higher VPA and less time spent sedentary, was associated with a higher CRF. Although having limited possibilities for causal inference, the present study provides important reference material of a general middle-aged population for future research, clinical practice, and public health work. Specifically, increased knowledge about specific sub-groups will aid in the personalized prescription of exercise in health care. In the light of a higher prevalence of low CRF in later years (3), a

For peer review only - http://bmjopen.bmj.com/site/about/guidelines.xhtml

great challenge remains in implementing effective interventions to support health in identified parts of the population.

DECLARATIONS

Funding

The main funding body of The Swedish CArdioPulmonary bioImage Study (SCAPIS) is the Swedish Heart-Lung Foundation (N/A). The study is also funded by the Knut and Alice Wallenberg Foundation (N/A), the Swedish Research Council (N/A) and VINNOVA (N/A) the University of Gothenburg and Sahlgrenska University Hospital (N/A), Karolinska Institutet and Stockholm county council (N/A), Linköping University and University Hospital (N/A), Lund University and Skåne University Hospital (N/A), Umeå University and University Hospital (N/A), Uppsala University and University Hospital (N/A). Author MB is supported by ALFgrants from Västra Götalandsregionen (ALFGBG-965249) and a grant from the Heart and Lung foundation (no 20210270). Author ÖE, EH and EEB were funded by grants from Skandia Risk & Hälsa (N/A). Author GB was supported by Heart and Lung foundation (20180324), the Swedish Research Council (2019-01140) and LUA/ALF (ALFGBG-718851).

Competing interests

The authors declare that they have no competing interests.

Authors' contributions

All authors contributed to the conception or design of the work. Mats Börjesson, Örjan Ekblom, Daniel Arvidsson, Daniel Väisänen, Elin Ekblom-Bak contributed to the acquisition, analysis, or interpretation of data for the work. Mats Börjesson, Örjan Ekblom, Emerald G. Heiland, Göran Bergström, Elin Ekblom-Bak drafted the manuscript or substantively revised

For peer review only - http://bmjopen.bmj.com/site/about/guidelines.xhtml

it. All authors revised it critically for important intellectual content. All authors critically revised the manuscript, gave final approval, and agreed to be accountable for all aspects of the work in ensuring that questions related to the accuracy or integrity of any part of the work are appropriately investigated and resolved. The corresponding author, Elin Ekblom-Bak, is the manuscripts guarantor and attests that all listed authors meet authorship criteria, that no others meeting the criteria have been omitted and take the full responsibility for the overall content.

Availability of data and material

Due to the nature of the sensitive personal data and study materials, they cannot be made freely available. However, by contacting the corresponding author or study organization (www.scapis.org), procedures for sharing data, analytic methods, and study materials for reproducing the results or replicating the procedure can be arranged following Swedish legislation.

Ethics approval

The study was approved by the ethics board (Dnr 2010-228-31M, Dnr 2011-449-32M and Dnr 638-16) and adheres to the Declaration of Helsinki. All participants provided written informed consent.

For peer review only - http://bmjopen.bmj.com/site/about/guidelines.xhtml

REFERENCES

1. Kodama S, Saito K, Tanaka S, Maki M, Yachi Y, Asumi M, et al. Cardiorespiratory fitness as a quantitative predictor of all-cause mortality and cardiovascular events in healthy men and women: a meta-analysis. Jama. 2009;301(19):2024-35.

2. Kandola A, Ashdown-Franks G, Stubbs B, Osborn DPJ, Hayes JF. The association between cardiorespiratory fitness and the incidence of common mental health disorders: A systematic review and meta-analysis. J Affect Disord. 2019;257:748-57.

3. Ekblom-Bak E, Ekblom O, Andersson G, Wallin P, Soderling J, Hemmingsson E, et al. Decline in cardiorespiratory fitness in the Swedish working force between 1995 and 2017. Scandinavian journal of medicine & science in sports. 2019;29(2):232-9.

4. Lamoureux NR, Fitzgerald JS, Norton KI, Sabato T, Tremblay MS, Tomkinson GR. Temporal Trends in the Cardiorespiratory Fitness of 2,525,827 Adults Between 1967 and 2016: A Systematic Review. Sports Med. 2019;49(1):41-55.

5. Bouchard C, An P, Rice T, Skinner JS, Wilmore JH, Gagnon J, et al. Familial aggregation of VO(2max) response to exercise training: results from the HERITAGE Family Study. Journal of applied physiology. 1999;87(3):1003-8.

6. Katzel LI, Sorkin JD, Fleg JL. A comparison of longitudinal changes in aerobic fitness in older endurance athletes and sedentary men. J Am Geriatr Soc. 2001;49(12):1657-64.

7. Lindgren M, Borjesson M, Ekblom O, Bergstrom G, Lappas G, Rosengren A. Physical activity pattern, cardiorespiratory fitness, and socioeconomic status in the SCAPIS pilot trial - A cross-sectional study. Prev Med Rep. 2016;4:44-9.

For peer review only - http://bmjopen.bmj.com/site/about/guidelines.xhtml

8. Zeiher J, Ombrellaro KJ, Perumal N, Keil T, Mensink GBM, Finger JD. Correlates and Determinants of Cardiorespiratory Fitness in Adults: a Systematic Review. Sports Med Open. 2019;5(1):39.

9. Ombrellaro KJ, Perumal N, Zeiher J, Hoebel J, Ittermann T, Ewert R, et al. Socioeconomic Correlates and Determinants of Cardiorespiratory Fitness in the General Adult Population: a Systematic Review and Meta-Analysis. Sports Med Open. 2018;4(1):25.

10. Ekblom O, Ekblom-Bak E, Bolam KA, Ekblom B, Schmidt C, Soderberg S, et al. Concurrent and predictive validity of physical activity measurement items commonly used in clinical settings--data from SCAPIS pilot study. BMC Public Health. 2015;15:978.

11. Hawkins S, Wiswell R. Rate and mechanism of maximal oxygen consumption decline with aging: implications for exercise training. Sports Med. 2003;33(12):877-88.

12. Bergstrom G, Berglund G, Blomberg A, Brandberg J, Engstrom G, Engvall J, et al. The Swedish CArdioPulmonary BioImage Study: objectives and design. J Intern Med. 2015;278(6):645-59.

13. Bjorkman F, Ekblom-Bak E, Ekblom O, Ekblom B. Validity of the revised Ekblom Bak cycle ergometer test in adults. Eur J Appl Physiol. 2016;116(9):1627-38.

14. Aguilar-Farias N, Brown WJ, Peeters GM. ActiGraph GT3X+ cut-points for identifying sedentary behaviour in older adults in free-living environments. J Sci Med Sport. 2014;17(3):293-9.

15. Sasaki JE, John D, Freedson PS. Validation and comparison of ActiGraph activity monitors. J Sci Med Sport. 2011;14(5):411-6.

16. Migueles JH, Cadenas-Sanchez C, Ekelund U, Delisle Nystrom C, Mora-Gonzalez J, Lof M, et al. Accelerometer Data Collection and Processing Criteria to Assess Physical Activity and Other Outcomes: A Systematic Review and Practical Considerations. Sports Med. 2017;47(9):1821-45.

For peer review only - http://bmjopen.bmj.com/site/about/guidelines.xhtml

17. Babor TF, de la Fuente JR, Saunders J, Grant M. AUDIT. The Alcohol Use Disorders Identification Test. Guidelines for use in primary health care. World Health Organization. 2001.

18. Bull FC, Al-Ansari SS, Biddle S, Borodulin K, Buman MP, Cardon G, et al. World Health Organization 2020 guidelines on physical activity and sedentary behaviour. British journal of sports medicine. 2020;54(24):1451-62.

19. Ekblom-Bak E, Borjesson M, Bergman F, Bergstrom G, Dahlin-Almevall A, Drake I, et al. Accelerometer derived physical activity patterns in 27.890 middle-aged adults: The SCAPIS cohort study. Scandinavian journal of medicine & science in sports. 2022;32(5):866-80.

20. Lewer D, Jayatunga W, Aldridge RW, Edge C, Marmot M, Story A, et al. Premature mortality attributable to socioeconomic inequality in England between 2003 and 2018: an observational study. Lancet Public Health. 2020;5(1):e33-e41.

21. Ekblom-Bak E, Vaisanen D, Ekblom B, Blom V, Kallings LV, Hemmingsson E, et al. Cardiorespiratory fitness and lifestyle on severe COVID-19 risk in 279,455 adults: a case control study. The international journal of behavioral nutrition and physical activity. 2021;18(1):135.

22. Vaisanen D, Kallings LV, Andersson G, Wallin P, Hemmingsson E, Ekblom-Bak E. Lifestyle-associated health risk indicators across a wide range of occupational groups: a cross-sectional analysis in 72,855 workers. BMC public health. 2020;20(1):1656.

23. Korshoj M, Lund Rasmussen C, de Oliveira Sato T, Holtermann A, Hallman D. Heart rate during work and heart rate variability during the following night: a day-by-day investigation on the physical activity paradox among blue-collar workers. Scand J Work Environ Health. 2021;47(5):387-94.

For peer review only - http://bmjopen.bmj.com/site/about/guidelines.xhtml

24. Vaisanen D, Kallings LV, Andersson G, Wallin P, Hemmingsson E, Ekblom-Bak E. Cardiorespiratory Fitness in Occupational Groups-Trends over 20 Years and Future Forecasts. Int J Environ Res Public Health. 2021;18(16).

25. Holtermann A, Krause N, van der Beek AJ, Straker L. The physical activity paradox: six reasons why occupational physical activity (OPA) does not confer the cardiovascular health benefits that leisure time physical activity does. Br J Sports Med. 2018;52(3):149-50.

26. Schantz P, Salier Eriksson J, Rosdahl H. Perspectives on Exercise Intensity, Volume and Energy Expenditure in Habitual Cycle Commuting. Front Sports Act Living. 2020;2:65.

27. Stigell E, Schantz P. Active Commuting Behaviors in a Nordic Metropolitan Setting in Relation to Modality, Gender, and Health Recommendations. Int J Environ Res Public Health. 2015;12(12):15626-48.

For peer review only

FIGURE LEGENDS

Figure 1. OR (95% CI) for low CRF in association with correlates in the backward multiregression model. OR are presented on log-scale. SED; sedentary, VPA; vigorous physical activity, perc; percent, Q; quartile.

Figure 2. Mean estimated VO_2max (95% CI) in association with age-group (x-axis) and VPA level in min/week (left) and % of time in sedentary (right).

For peer review only

For peer review only - http://bmjopen.bmj.com/site/about/guidelines.xhtml

APPENDICE SUBMITTED

Additional File 1 – Questions used from SCAPIS study

Appendix Table 1 - Sex-specific estimated VO_2max (ml/min/kg) in association with all correlates presented in Table 1 to 3.

Appendix Figure 1 – Association between continuous levels of estimated VO_2max, BMI and waist circumference.

Appendix Figure 2 - Association between estimated VO_2max and continuous levels of accelerometer assessed sedentary time and physical activity of different intensities.

For peer review only - http://bmjopen.bmj.com/site/about/guidelines.xhtml

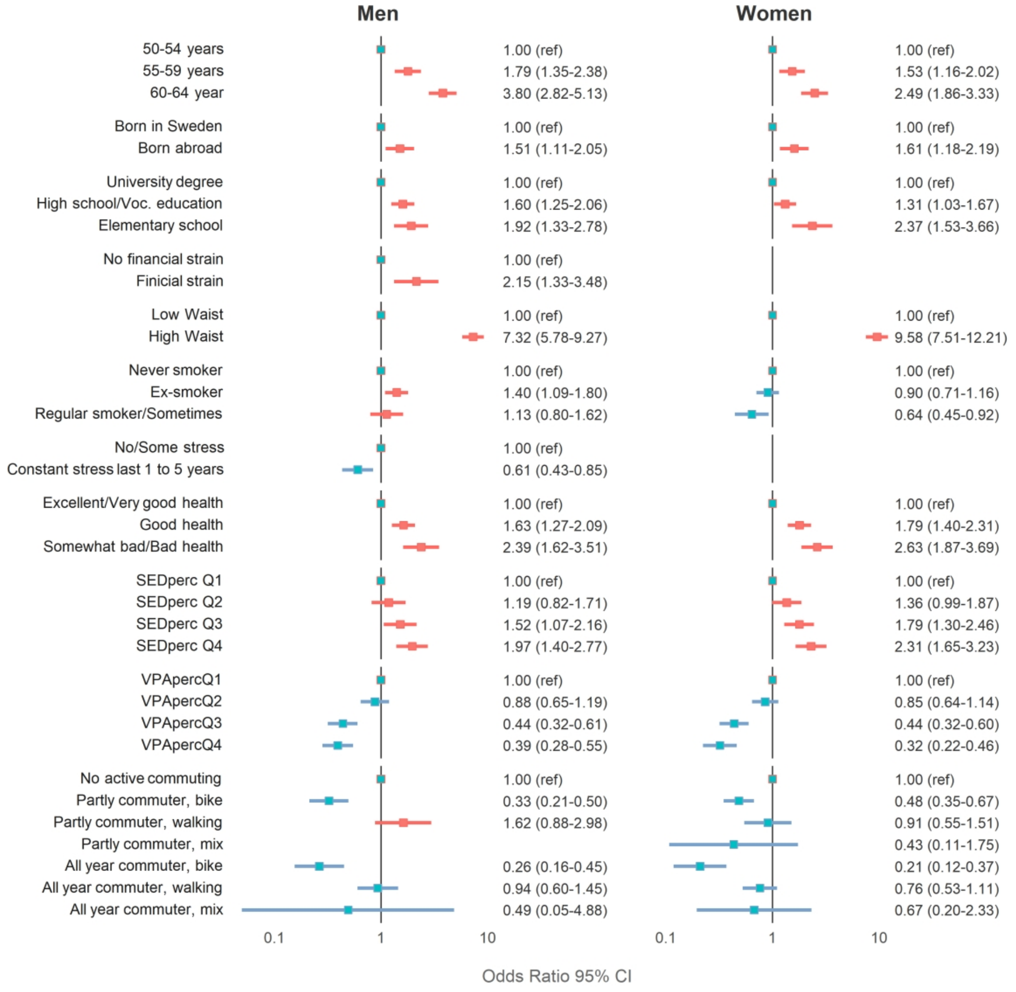

OR (95% CI) for low CRF in association with correlates in the backward multiregression model. OR are presented on log-scale. SED; sedentary, VPA; vigorous physical activity, perc; percent, Q; quartile.

517x517mm (157 x 157 DPI)

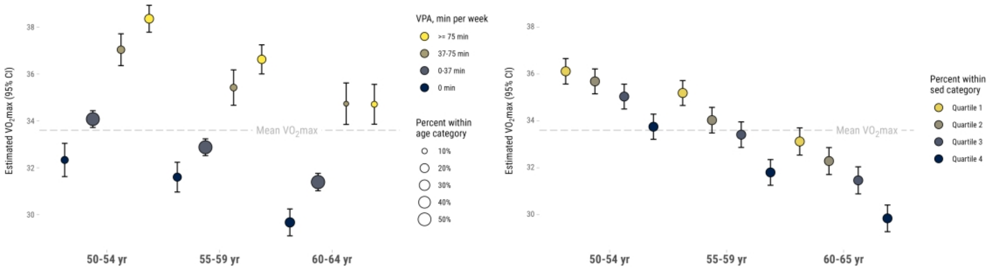

Mean estimated VO2max (95% CI) in association with age-group (x-axis) and VPA level in min/week (left) and % of time in sedentary (right).

1975x564mm (72 x 72 DPI)

Additional file 1. Questions from the SCAPIS questionnaire used in the present analyses. Reference to the original question is given if available.

Educational level

What is your highest completed level of education?

No formal education degree
Elementary school
High school / Vocational education
University degree

Employment status

What is your current occupation?

Employed, _____% of fulltime
On leave / parental leave
Studying
Labour market measure
Unemployed
Retired
Early or sick retiree

Financial strain

If you were to suddenly end up in a situation where you had to raise SEK 20,000 (≈EURO 2,000) in one week, would you be able to do it?

Yes
No

Strandhagen, E., et al. (2010). "Selection bias in a population survey with registry linkage: potential effect on socioeconomic gradient in cardiovascular risk." Eur J Epidemiol 25(3): 163-172.

Marital status

What is your current marital status?

Alone
Divorced
Married/Cohabitat
Widow

Born in Sweden

Are you born in Sweden?

Yes
No

Smoking habits

Do you smoke?

No, have never smoked
Yes, smokes regularly
No, stopped smoking
Yes, smokes occasionally

For peer review only

Commuting habits

Mode of travel to work:

Spring

Car
Bus/tram/train
Bicycling
Walking

Summer

Car
Bus/tram/train
Bicycling
Walking

Autumn

Car
Bus/tram/train
Bicycling
Walking

Winter

Car
Bus/tram/train
Bicycling
Walking

Wennberg, P., et al (2006). The effects of commuting activity and occupational and leisure time physical activity on risk of myocardial infarction. European Journal of Cardiovascular Prevention & Rehabilitation December 2006 13: 924-930

Sleep

How well are you sleeping usually?

Very well
Well
Rather well
Badly
Very badly

Adapted from the Basic Nordics Sleep Questionnaire Partinen, M. and T. Gislason (1995). "Basic Nordic Sleep Questionnaire (BNSQ): a quantitated measure of subjective sleep complaints." J Sleep Res 4(S1): 150-155.

Stress

By stress we mean feeling tense, irritable, anxious or having sleeping difficulties as a result of conditions at work or at home. Did you experience this?

Never experienced stress
≥1 period of stress
≥1 period of stress during the last 5 years
several periods of stress during the last 5 years
permanent stress during the last year or the last 5 years

For peer review only

Adapted from: Rosengren, A., et al. (2004). "Association of psychosocial risk factors with risk of acute myocardial infarction in 11119 cases and 13648 controls from 52 countries (the INTERHEART study): case-control study." Lancet 364(9438): 953-962.

Rosengren, A., et al. (1991). "Self-perceived psychological stress and incidence of coronary artery disease in middle-aged men." Am J Cardiol 68(11): 1171-1175.

Control at work

At work I feel that I have control over what happens in most situations

Strongly disagree
Disagree
Neutral
Agree
Strongly agree

Adapted from: Rosengren, A., et al. (2004). "Association of psychosocial risk factors with risk of acute myocardial infarction in 11119 cases and 13648 controls from 52 countries (the INTERHEART study): case-control study." Lancet 364(9438): 953-962.

Bobak, M., et al. (2000). "Socioeconomic factors, material inequalities, and perceived control in self-rated health: cross-sectional data from seven post-communist countries." Soc Sci Med 51(9): 1343-1350. + references therein.

Control in life

I feel that what happens in my life is often determined by factors beyond my control

Strongly disagree
Disagree
Neutral
Agree
Strongly agree

Adapted from: Rosengren, A., et al. (2004). "Association of psychosocial risk factors with risk of acute myocardial infarction in 11119 cases and 13648 controls from 52 countries (the INTERHEART study): case-control study." Lancet 364(9438): 953-962.

Bobak, M., et al. (2000). "Socioeconomic factors, material inequalities, and perceived control in self-rated health: cross-sectional data from seven post-communist countries." Soc Sci Med 51(9): 1343-1350. + references therein.

General health

In general, would you say your health is:

Excellent
Very good
Good
Fair
Poor

Adapted from the Swedish version of the SF-36 questionnaire.
Sullivan, M., et al. (1995). "The Swedish SF-36 Health Survey--I. Evaluation of data quality, scaling assumptions, reliability and construct validity across general populations in Sweden." Soc Sci Med 41(10): 1349-1358.

Sullivan, M. and J. Karlsson (1998). "The Swedish SF-36 Health Survey III. Evaluation of criterion-based validity: results from normative population." J Clin Epidemiol 51(11): 1105-1113.

For peer review only

Persson, L. O., et al. (1998). "The Swedish SF-36 Health Survey II. Evaluation of clinical validity: results from population studies of elderly and women in Gothenborg." J Clin Epidemiol 51(11): 1095-1103

Weight at 20 years of age

What was your approximate weight when you were age 20?

____kg

Depression symptoms

During the past 4 weeks, have you had any of the following problems with your work or other regular daily activities as a result of any emotional problems (such as feeling depressed or anxious)?

Yes
No

Adapted from the Swedish version of the SF-36 questionnaire.

Sullivan, M., et al. (1995). "The Swedish SF-36 Health Survey--I. Evaluation of data quality, scaling assumptions, reliability and construct validity across general populations in Sweden." Soc Sci Med 41(10): 1349-1358.

Sullivan, M. and J. Karlsson (1998). "The Swedish SF-36 Health Survey III. Evaluation of criterion-based validity: results from normative population." J Clin Epidemiol 51(11): 1105-1113.

Persson, L. O., et al. (1998). "The Swedish SF-36 Health Survey II. Evaluation of clinical validity: results from population studies of elderly and women in Gothenborg." J Clin Epidemiol 51(11): 1095-1103

Prevalent chronic conditions

Which of these diseases has a doctor diagnosed you or have you been surgically treated for?

Myocardial infarction
Angina pectoris
Atrial fibrillation
Heart failure
Heart valve disease
CABG or PCI
Intervention of arterial stenosis other than coronary artery stenosis
Aortic intervention
Stroke
Hypertension
Dyslipidemia
Diabetes
Doctor-diagnosed COPD, chronic bronchitis or emphysema
Other lung disease
Rheumatic disease
Cancer

Physical working situation

Degree of physical activity at work

Physically heavy most of the time
Sometimes physically heavy
Light and mobile

For peer review only

Light but partly mobile
Sedentary or standing

Wareham, N. J., et al. (2003). "Validity and repeatability of a simple index derived from the short physical activity questionnaire used in the European Prospective Investigation into Cancer and Nutrition (EPIC) study." Public Health Nutr 6(4): 407-413.

InterAct, C., et al. (2012). "Validity of a short questionnaire to assess physical activity in 10 European countries." Eur J Epidemiol 27(1): 15-25.

Exercise habits

How often have you exercised or exercised in workout clothes in the last three months, in order to improve your fitness and/or to feel good?
Never
Occasionally - not regularly
1-2 times a week
2-3 times a week
More than 3 times per week

Wareham, N. J., et al. (2003). "Validity and repeatability of a simple index derived from the short physical activity questionnaire used in the European Prospective Investigation into Cancer and Nutrition (EPIC) study." Public Health Nutr 6(4): 407-413.

InterAct, C., et al. (2012). "Validity of a short questionnaire to assess physical activity in 10 European countries." Eur J Epidemiol 27(1): 15-25.

Total physical activity

How much physical movement and exertion have you had in the last 12 months?
Sedentary
Light exercise
Moderate exercise
Regular, vigorous exercise

Sweden PHao. The Swedish national public health survey. http://www.folkhalsomyndigheten.se/documents/statistik-uppfoljning/enkater-undersokningar/nationella-folkhalsoenkaten/frageformular/formular-nationella-folkhalsoenkaten-2012.pdf. 2012.

Leisure time sitting

The last question is about the time you spent sitting on weekdays during the last 7 days. Include time spent at work, at home, while doing course work and during leisure time. This may include time spent sitting at a desk, visiting friends, reading, or sitting or lying down to watch television. How much time did you spend sitting on a day?
____minutes

Adapted from the IPAQ questionnaire: Craig, C. L., et al. (2003). "International physical activity questionnaire: 12-country reliability and validity." Med Sci Sports Exerc 35(8): 1381-1395.

For peer review only

Appendix Table 1. Sex-specific estimated VO_2max (ml/min/kg) in association with all correlates presented in Table 1 to 3.

	Estimated VO_2max				Low estimated VO_2max		
	Men		Women		Men	Women	p-value for interaction
**Age**	n	Median (95% CI)	n	Median (95% CI)	OR[a] (95% CI)	OR (95% CI)	p=0.045
50-54 years	906	37.8 (37.3-38.3)	975	31.9 (31.4-32.3)	1 (ref)	1 (ref)	
55-59 years	885	36.3 (35.7-36.8)	927	30.5 (30.0-31.0)	1.49 (1.21-1.84)	1.44 (1.18-1.75)	
60-64 years	799	33.9 (33.6-34.7)	816	28.6 (28.1-29.1)	2.98 (2.42-3.66)	2.23 (1.83-2.73)	
**Civil status**							
Married/Cohabitation	1935	36.5 (36.0-36.8)	1831	30.7 (30.4-31.2)	1 (ref)	1 (ref)	p=0.655
Divorced/Single/Widower	610	35.3 (34.7-36.1)	851	29.7 (29.1-30.2)	1.43 (1.17-1.73)	1.34 (1.12-1.59)	
**Born in Sweden**							
Yes	2090	36.5 (36.1-36.8)	2251	30.7 (30.4-31.1)	1 (ref)	1 (ref)	p=0.633
No	472	34.9 (34.1-35.5)	451	28.9 (28.3-29.3)	1.85 (1.49-2.28)	1.68 (1.36-2.07)	
**Educational level**							
University degree	1087	37.5 (37.0-38.0)	1393	31.5 (31.2-32.0)	1 (ref)	1 (ref)	p=0.294
High school/Vocational education	1147	35.5 (35.1-35.9)	1068	29.5 (29.1-29.9)	1.86 (1.54-2.24)	1.83 (1.54-2.18)	
Elementary school	321	34.0 (33.3-34.8)	234	27.4 (26.5-28.6)	2.54 (1.95-3.32)	3.21 (2.41-4.28)	
**Employment**							
Working, ≥ 50% of full-time	2117	36.7 (36.5-37.0)	2225	31.0 (30.6-31.4)	1 (ref)	1 (ref)	p=0.574
Retired	123	33.3 (32.1-34.7)	108	27.6 (26.6-29.0)	1.48 (1.01-2.19)	1.60 (1.07-2.41)	
Disability pension/Sickness pension/Sick leave	115	33.7 (32.9-34.8)	184	26.7 (26.0-28.0)	2.16 (1.47-3.18)	2.91 (2.14-3.97)	
Unemployed/Student	191	33.6 (32.6-34.5)	174	29.3 (28.4-30.1)	2.27 (1.67-3.09)	1.53 (1.11-2.11)	
**Financial strain**							
No	2328	36.5 (36.1-36.8)	2431	30.7 (30.1-31.1)	1 (ref)	1 (ref)	p=0.293
Yes	177	33.0 (31.9-34.3)	229	28.0 (26.9-28.7)	2.91 (2.11-4.00)	2.24 (1.69-2.96)	

For peer review only

**Smoking habits**							
Never smoker	1287	37.3 (36.8-37.9)	1176	31.4 (31.0-31.8)	1 (ref)	1 (ref)	p=0.004
Ex-smoker	897	35.0 (34.6-35.5)	1118	29.9 (29.3-30.3)	1.65 (1.37-1.99)	1.17 (0.98-1.39)	
Regular smoker/Sometimes	341	35.1 (34.0-36.1)	375	29.9 (29.3-30.3)	2.02 (1.57-2.60	1.28 (0.99-1.64)	
**Pack-years**							
Never smokers (0)	1287	37.3 (36.8-37.9)	1176	31.4 (31.0-31.8)	1 (ref)	1 (ref)	p=0.008
Former smokers, low (<15 pack-years)	500	36.1 (35.4-36.7)	720	30.5 (30.1-31.1)	1.13 (0.90-1.43)	0.89 (0.73-1.09)	
Former smokers, heavy (≥15 pack-years)	397	33.4 (33.1-34.3)	398	28.1 (27.6-29.1)	2.57 (2.03-3.27)	1.82 (1.43-2.30)	
Current smokers, low (<15 pack-years)	106	36.5 (34.8-37.6)	120	31.6 (30.0-32.9)	1.57 (1.02-2.40)	0.79 (0.51-1.22)	
Current smokers, heavy (≥15 pack-years)	235	34.8 (33.6-35.6)	255	29.3 (28.3-29.9)	2.26 (1.69-3.02)	1.56 (1.18-2.07)	
**Alcohol abuse**							
No/Low	1881	36.6 (36.3-36.9)	2067	31.0 (30.5-31.3)	1 (ref)	1 (ref)	p=0.226
Moderate	355	35.4 (34.8-36.4)	284	30.8 (30.1-31.3)	1.43 (1.12-1.82)	1.08 (0.82-1.42)	
High	22	33.7 (31.1-35.0)	11	27.3 (24.6-33.3)	2.72 (1.13-6.50)	3.92 (1.18-13.04)	
**Sleep**							
Very well/well	1348	36.7 (36.2-37.0)	1053	30.8 (30.3-31.4)	1 (ref)	1 (ref)	p=0.253
Rather well/Badly/Very badly	1198	35.7 (35.4-36.1)	1637	30.2 (29.9-30.5)	1.31 (1.11-1.56)	1.14 (0.97-1.35)	
**Stress**							
No stress/Some stress during last 5 years	2140	36.3 (35.9-36.7)	1949	30.5 (30.3-31.0)	1 (ref)	1 (ref)	p=0.267
Constant stress last 1 to 5 years	400	35.6 (35.1-36.6)	729	29.9 (29.4-30.6)	1.14 (0.91-1.44)	1.32 (1.10-1.58)	
**At work I feel that that I have control in most situations**							
Strongly agree/Agree	1993	36.5 (36.1-36.8)	1853	30.8 (30.4-31.2)	1 (ref)	1 (ref)	p=0.730
Neutral/Do not agree/Do not agree at all	444	35.9 (35.1-36.6)	667	30.1 (29.5-30.9)	1.20 (0.96-1.50)	1.25 (1.04-1.51)	
**I feel that what happens in my life is often determined by factors beyond my control**							
Do not agree at all/Do not agree	1489	36.5 (36.1-36.9)	1487	31.3 (30.9-31.7)	1 (ref)	1 (ref)	p=0.004
Neutral/Agree/Strongly agree	1022	35.9 (35.4-36.4)	1171	29.3 (28.9-29.7)	1.22 (1.03-1.45)	1.72 (1.45-2.02)	

For peer review only

**General health**							
Excellent/Very good	1281	38.0 (37.5-38.3)	1417	32.1 (31.8-32.6)	1 (ref)	1 (ref)	p=0.116
Good	966	34.9 (34.5-35.4)	852	29.1 (28.6-29.7)	2.31 (1.91-2.79)	2.52 (2.08-3.04)	
Somewhat bad/Bad	300	33.3 (32.3-34.0)	429	26.4 (25.7-27.3)	4.13 (3.15-5.41)	5.40 (4.27-6.83)	
**BMI (kg·m^{-2}) and estVO$_2$max in ml·min^{-1}·kg^{-1}**							
<24.9	1287	37.3 (36.8-37.9)	1176	31.4 (31.0-31.8)	1 (ref)	1 (ref)	
25.0-29.9	1391	35.8 (35.5-36.3)	950	28.7 (28.4-29.0)	4.35 (3.34-5.68)	7.94 (6.25-10.10)	
30.0-34.9	379	31.4 (30.9-32.1)	349	24.7 (24.3-25.1)	27.7 (19.7-38.9)	47.7 (34.1-66.9)	
≥35	72	27.1 (26.5-27.9)	131	21.9 (21.4-22.2)	-	-	
**BMI and estVO$_2$max in L·min^{-1}**							
<24.9	748	3.07 (3.03-3.12)	1288	2.16 (2.14-2.18)	1 (ref)	1 (ref)	p=0.001
25.0-29.9	1391	3.16 (3.13-3.19)	950	2.14 (2.12-2.17)	1.87 (1.46-2.40)	2.70 (2.11-3.45)	
30.0-34.9	379	3.21 (3.16-3.27)	349	2.18 (2.13-2.23)	4.15 (2.70-6.37)	7.36 (4.85-11.17)	
≥35	72	3.18 (3.06-3.30)	131	2.20 (2.10-2.25)	12.7 (5.7-28.3)	18.2 (9.63-34.3)	
**Waist circumference**							
Low waist (<88 cm women, <102 cm men)	1725	38.5 (38.2-38.9)	1466	34.0 (33.6-34.4)	1 (ref)	1 (ref)	p=0.041
High waist (≥88 cm women, ≥102cm men)	863	32.0 (31.6-32.5)	1251	26.7 (26.4-27.0)	9.15 (7.52-11.1)	11.5 (9.44-14.1)	
**Current weight compared to weight at 20 years of age**							
Less now compared to 20 years of age	102	41.3 (40.1-42.3)	167	36.9 (36.5-38.2)	1 (ref)	1 (ref)	p<0.001
0 to 10 kg more now compared at 20 years of age	813	39.8 (39.4-40.3)	926	34.2 (33.8-34.7)	0.96 (0.52-1.77)	1.22 (0.67-2.20)	
10 to 20 kg more now than at 20 years of age	920	35.8 (35.5-36.3)	867	29.8 (29.4-30.1)	3.16 (1.75-5.72)	5.28 (2.98-9.36)	
>20 kg more now than at 20 years of age	585	31.7 (31.2-32.3)	646	24.8 (24.5-25.2)	14.5 (7.9-26.6)	36.7 (20.5-65.7)	
**Depression symptoms**							
No	2003	36.3 (35.9-36.7)	1684	30.8 (30.4-31.3)	1 (ref)	1 (ref)	p=0.230
Yes	519	36.0 (35.4-36.6)	976	29.9 (29.3-30.2)	1.21 (0.99-1.50)	1.41 (1.19-1.67)	

For peer review only

**Chronic conditions***							
0	1693	36.8 (36.4-37.1)	1772	31.1 (30.7-31.4)	1 (ref)	1 (ref)	p=0.186
1-2	816	35.1 (34.7-35.7)	889	29.3 (28.8-29.9)	1.30 (1.09-1.56)	1.46 (1.23-1.74)	
=>3	81	34.0 (32.7-35.3)	57	23.8 (22.6-29.1)	1.62 (1.02-2.56)	2.92 (1.70-5.03)	
**CVD, Hypertension and/or Lung disease**							
No	1980	36.6 (36.2-36.9)	2089	31.0 (30.5-31.3)	1 (ref)	1 (ref)	p=0.142
Yes	610	35.0 (34.6-35.6)	629	28.8 (28.3-29.4)	1.26 (1.04-1.53)	1.58 (1.31-1.90)	
**Commuting***							
No active commuting	1638	35.4 (35.1-35.8)	1419	29.9 (29.5-30.3)	1 (ref)	1 (ref)	p=0.831
Partly commuter, bike	270	38.6 (37.7-39.1)	401	32.1 (31.6-33.0)	0.35 (0.25-0.48)	0.45 (0.34-0.58)	
Partly commuter, walking	68	34.5 (32.7-35.8)	128	28.9 (28.1-29.8)	1.42 (0.86-2.33)	0.99 (0.68-1.44)	
Partly commuter, mix	3	44.2 (38.1-50.9)	16	29.8 (28.2-33.2)	-	0.57 (0.18-1.79)	
All year commuter, bike	203	41.3 (40.5-42.4)	204	35.0 (34.2-36.2)	0.23 (0.15-0.35)	0.14 (0.08-0.23)	
All year commuter, walking	162	36.9 (35.6-38.1)	253	30.3 (29.7-31.3)	0.79 (0.56-1.12)	0.66 (0.49-0.89)	
All year commuter, mix	8	40.2 (36.8-41.1)	22	32.3 (29.0-36.1)	0.26 (0.03-2.18)	0.61 (0.24-1.59)	
**Physical working situation**							
Sedentary to light	1894	36.7 (36.3-37.0)	2027	31.0 (30.5-31.4)	1 (ref)	1 (ref)	p=0.775
Sometimes to frequently heavy	498	35.3 (34.8-36.1)	447	29.8 (29.1-30.4)	1.48 (1.20-1.83)	1.40 (1.12-1.74)	
**Exercise habits**							
Never	722	33.7 (33.4-34.1)	662	27.4 (26.7-28.0)	1 (ref)	1 (ref)	p=0.333
Irregular	496	34.8 (34.0-35.3)	571	28.7 (28.2-29.3)	0.78 (0.62-0.99)	0.71 (0.57-0.90)	
1-2 times per week	456	36.7 (36.1-37.3)	576	30.7 (30.3-31.4)	0.35 (0.27-0.46)	0.34 (0.26-0.43)	
2-3 times per week	507	37.9 (37.2-38.5)	582	33.0 (32.3-33.6)	0.29 (0.22-0.38)	0.22 (0.17-0.28)	
>3 times per week	356	40.5 (39.7-41.0)	293	35.4 (34.6-36.2)	0.14 (0.10-0.20)	0.14 (0.10-0.21)	

For peer review only

**Total physical activity**							
Sedentary	315	32.4 (31.5-33.2)	225	25.5 (24.5-26.5)	1 (ref)	1 (ref)	p=0.021
Light	1098	34.9 (34.6-35.3)	1268	28.8 (28.4-29.1)	0.42 (0.32-0.55)	0.34 (0.25-0.46)	
Moderate	735	37.4 (37.0-38.2)	878	32.7 (32.2-33.2)	0.18 (0.14-0.25)	0.10 (0.07-0.14)	
Regular exercise/training	367	40.9 (40.1-41.5)	302	35.6 (35.0-36.2)	0.06 (0.04-0.10)	0.06 (0.04-0.09)	
**Leisure time sitting**							
Q1; <4 h per day	525	37.5 (37.0-38.1)	596	30.9 (30.1-31.7)	1 (ref)	1 (ref)	p=0.159
Q2; 4-6 h per day	499	36.2 (35.7-37.0)	540	30.8 (30.1-31.4)	1.29 (0.98-1.69)	1.19 (0.92-1.54)	
Q3; 6-8.5 h per day	438	36.9 (36.1-37.9)	487	31.3 (30.5-32.0)	1.06 (0.79-1.41)	1.16 (0.88-1.52)	
Q4; >8.5 h per day	527	36.3 (35.6-37.0)	431	30.3 (29.9-31.2)	1.18 (0.89-1.55)	1.46 (1.11-1.93)	
**% of time spent in Sedentary**							
Q1; <47%	462	37.5 (36.8-38.1)	805	31.9 (31.1-32.3)	1 (ref)	1 (ref)	p=0.033
Q2; 47%-54%	539	37.1 (36.6-37.7)	726	30.8 (30.2-31.4)	1.11 (0.83-1.48)	1.23 (0.97-1.54)	
Q3; 54%-61%	650	36.4 (35.8-37.0)	616	30.1 (29.5-30.7)	1.30 (0.99-1.71)	1.66 (1.31-2.09)	
Q4; >61%	803	34.8 (34.3-35.5)	464	27.9 (27.3-28.4)	1.97 (1.52-2.55)	3.01 (2.35-3.85)	
**% of time spent in prolonged Sedentary (of total wear time)**							
Q1; <13%	474	37.6 (37.0-38.2)	792	31.7 (30.8-32.2)	1 (ref)	1 (ref)	p=0.210
Q2; 13%-19%	536	37.2 (36.6-37.6)	731	31.1 (30.5-31.7)	1.24 (0.93-1.66)	1.08 (0.86-1.35)	
Q3; 19%-26%	673	36.1 (35.5-36.7)	592	30.1 (29.5-30.5)	1.55 (1.18-2.03)	1.64 (1.30-2.07)	
Q4; >26%	771	34.9 (34.4-35.5)	496	28.1 (27.7-28.6)	2.00 (1.54-2.60)	2.42 (1.90-3.08)	
**% of time spent in Light physical activity**							
Q1; <33%	840	35.4 (34.8-35.8)	427	28.6 (27.9-29.7)	1 (ref)	1 (ref)	p=0.124
Q2; 33%-40%	629	36.1 (35.5-36.7)	637	30.3 (29.8-31.0)	0.87 (0.70-1.09)	0.66 (0.51-0.85)	
Q3; 40%-46%	542	37.1 (36.7-37.8)	723	30.6 (30.0-31.2)	0.64 (0.50-0.81)	0.53 (0.41-0.69)	
Q4; >46%	443	37.0 (36.4-37.6)	824	31.0 (30.5-31.8)	0.67 (0.52-0.86)	0.46 (0.36-0.59)	

For peer review only

**% of time spent in Moderate physical activity**							
Q1; <3.8%	655	34.7 (34.1-35.3)	612	28.3 (27.7-28.9)	1 (ref)	1 (ref)	p=0.062
Q2; 3.8%-5.3%	581	36.4 (35.7-37.0)	684	30.1 (29.7-30.7)	0.61 (0.48-0.78)	0.61 (0.49-0.77)	
Q3; 5.3%-7.2%	594	37.0 (36.2-37.7)	672	31.0 (30.3-31.5)	0.56 (0.44-0.71)	0.48 (0.38-0.61)	
Q4; >7.2%	624	37.2 (36.7-37.9)	643	32.1 (31.3-32.5)	0.51 (0.40-0.65)	0.38 (0.30-0.48)	
**% of time spent in Vigorous physical activity**							
Q1;<0.02%	557	33.7 (33.3-34.2)	709	28.0 (27.4-28.5)	1 (ref)	1 (ref)	p=0.060
Q2; 0.02%-0.1%	566	34.9 (34.1-35.6)	700	28.7 (28.2-29.4)	0.79 (0.62-1.00)	0.82 (0.67-1.02)	
Q3; 0.1%-0.6%	640	37.1 (36.6-37.6)	627	31.4 (31.0-31.9)	0.37 (0.29-0.47)	0.31 (0.25-0.40)	
Q4; >0.6%	691	38.7 (38.1-39.4)	575	34.7 (33.9-35.3)	0.26 (0.20-0.33)	0.17 (0.13-0.23)	

[a] Adjusted for sex and age, comparing the lowest sex-specific tertile of estimated VO_2max with the two higher tertiles

[#] Sex-specific tertiles based on L/min. Analyses additionally adjusted for weight in kg.

For peer review only

Appendix Figure 1. Association between continous levels of estimated VO2max, BMI and waist circumference.

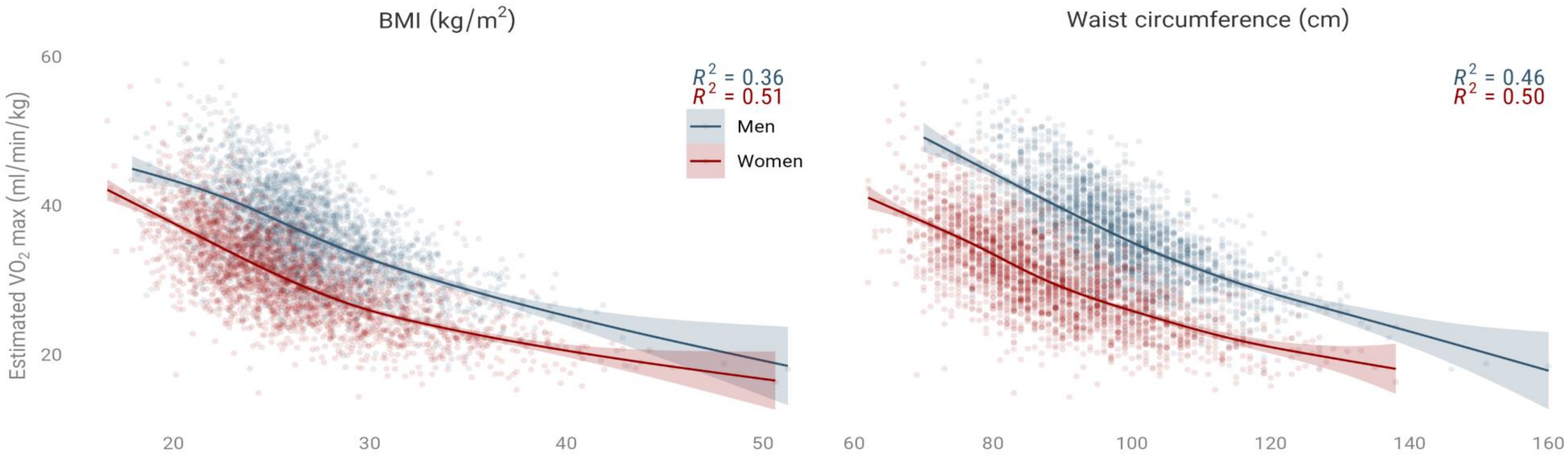

Appendix Figure 2. Association between estimated VO2max and continuous levels of accelerometer assessed sedentary time and physical activity of different intensities.

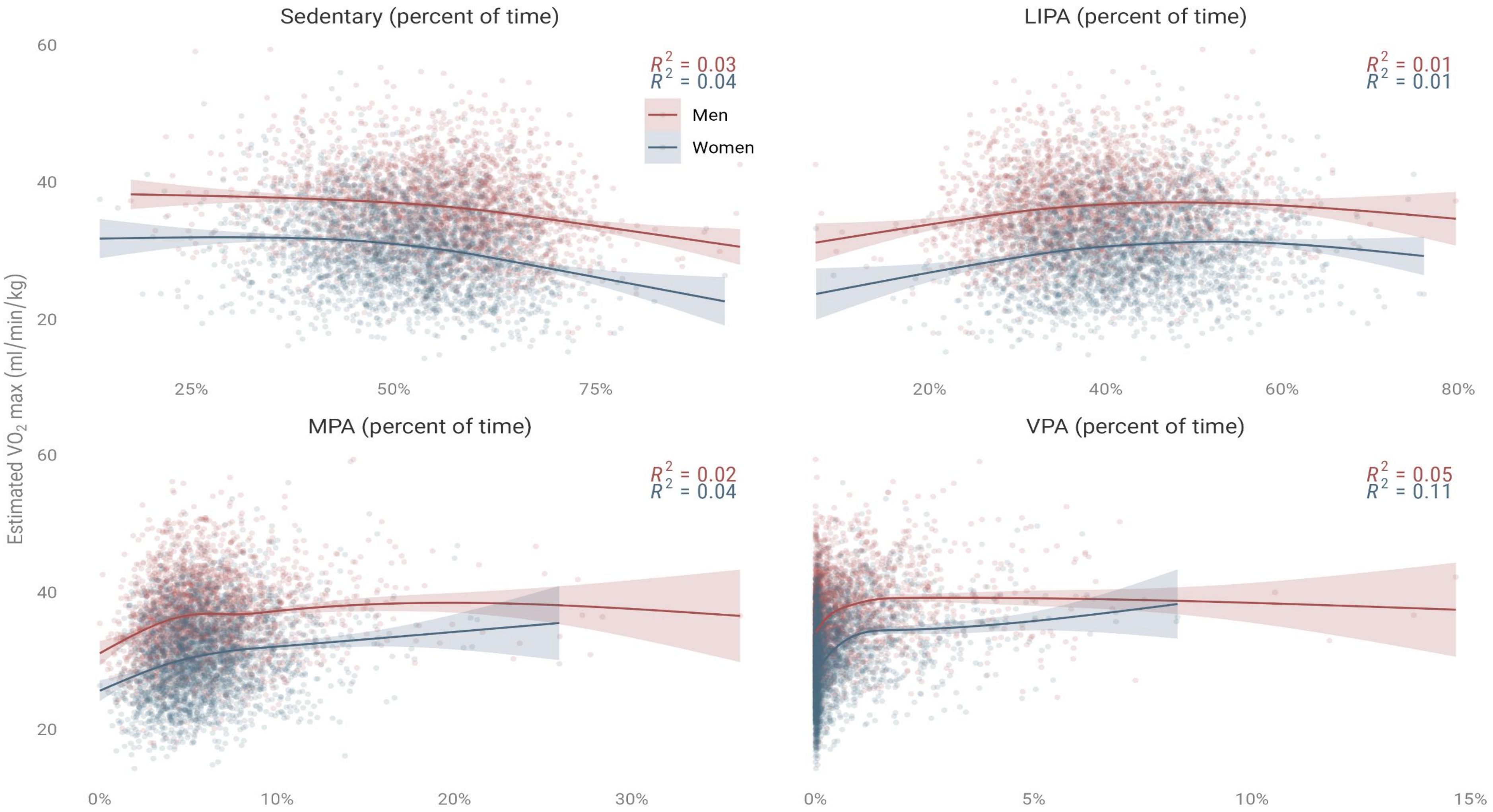

STROBE Statement—checklist of items that should be included in reports of observational studies

	Item No.	Recommendation	Page No.	Relevant text from manuscript
**Title and abstract**	1	(*a*) Indicate the study's design with a commonly used term in the title or the abstract	2	
		(*b*) Provide in the abstract an informative and balanced summary of what was done and what was found	2	
**Introduction**				
Background/rationale	2	Explain the scientific background and rationale for the investigation being reported	4	
Objectives	3	State specific objectives, including any prespecified hypotheses	4-5	
**Methods**				
Study design	4	Present key elements of study design early in the paper	5	
Setting	5	Describe the setting, locations, and relevant dates, including periods of recruitment, exposure, follow-up, and data collection	5	
Participants	6	*Cohort study*—Give the eligibility criteria, and the sources and methods of selection of participants. Describe methods of follow-up<br>***Case-control study*—Give the eligibility criteria, and the sources and methods of case ascertainment and control selection. Give the rationale for the choice of cases and controls**<br>*Cross-sectional study*—Give the eligibility criteria, and the sources and methods of selection of participants	5	
		(*b*) *Cohort study*—For matched studies, give matching criteria and number of exposed and unexposed<br>*Case-control study*—For matched studies, give matching criteria and the number of controls per case		
Variables	7	Clearly define all outcomes, exposures, predictors, potential confounders, and effect modifiers. Give diagnostic criteria, if applicable	5-7	
Data sources/ measurement	8*	For each variable of interest, give sources of data and details of methods of assessment (measurement). Describe comparability of assessment methods if there is more than one group	5-7	
Bias	9	Describe any efforts to address potential sources of bias	7-8	
Study size	10	Explain how the study size was arrived at	8	

Continued on next page

For peer review only

Quantitative variables	11	Explain how quantitative variables were handled in the analyses. If applicable, describe which groupings were chosen and why	7-8
Statistical methods	12	(*a*) Describe all statistical methods, including those used to control for confounding	7-8
		(*b*) Describe any methods used to examine subgroups and interactions	7-8
		€ Explain how missing data were addressed	
		(*d*) *Cohort study*—If applicable, explain how loss to follow-up was addressed<br>***Case-control study*—If applicable, explain how matching of cases and controls was addressed**<br>*Cross-sectional study*—If applicable, describe analytical methods taking account of sampling strategy	7-8
		€ Describe any sensitivity analyses	
**Results**			
Participants	13*	(a) Report numbers of individuals at each stage of study—eg numbers potentially eligible, examined for eligibility, confirmed eligible, included in the study, completing follow-up, and analysed	8
		(b) Give reasons for non-participation at each stage	8
		© Consider use of a flow diagram	
Descriptive data	14*	(a) Give characteristics of study participants (eg demographic, clinical, social) and information on exposures and potential confounders	8-9
		(b) Indicate number of participants with missing data for each variable of interest	8-9
		(c) *Cohort study*—Summarise follow-up time (eg, average and total amount)	
Outcome data	15*	*Cohort study*—Report numbers of outcome events or summary measures over time	
		Case-control study—Report numbers in each exposure category, or summary measures of exposure	
		Cross-sectional study—Report numbers of outcome events or summary measures	
Main results	16	(*a*) Give unadjusted estimates and, if applicable, confounder-adjusted estimates and their precision (eg, 95% confidence interval). Make clear which confounders were adjusted for and why they were included	8-11
		(*b*) Report category boundaries when continuous variables were categorized	8-11
		(*c*) If relevant, consider translating estimates of relative risk into absolute risk for a meaningful time period	

Continued on next page

For peer review only

Other analyses	17	Report other analyses done—eg analyses of subgroups and interactions, and sensitivity analyses	8-11
**Discussion**			
Key results	18	Summarise key results with reference to study objectives	11-14
Limitations	19	Discuss limitations of the study, taking into account sources of potential bias or imprecision. Discuss both direction and magnitude of any potential bias	14
Interpretation	20	Give a cautious overall interpretation of results considering objectives, limitations, multiplicity of analyses, results from similar studies, and other relevant evidence	11-14
Generalisability	21	Discuss the generalizability (external validity) of the study results	15
**Other information**			
Funding	22	Give the source of funding and the role of the funders for the present study and, if applicable, for the original study on which the present article is based	16

*Give information separately for cases and controls in case-control studies and, if applicable, for exposed and unexposed groups in cohort and cross-sectional studies.

Note: An Explanation and Elaboration article discusses each checklist item and gives methodological background and published examples of transparent reporting. The STROBE checklist is best used in conjunction with this article (freely available on the Web sites of PLoS Medicine at http://www.plosmedicine.org/, Annals of Internal Medicine at http://www.annals.org/, and Epidemiology at http://www.epidem.com/). Information on the STROBE Initiative is available at www.strobe-statement.org.

For peer review only

BMJ Open

Correlates of cardiorespiratory fitness in a population-based sample of middle-aged adults – cross-sectional analyses in the SCAPIS study

Journal:	*BMJ Open*
Manuscript ID	bmjopen-2022-066336.R1
Article Type:	Original research
Date Submitted by the Author:	05-Nov-2022
Complete List of Authors:	Börjesson, Mats; University of Gothenburg, Center for Health and Performance, Department of Molecular and Clinical Medicine,; Sahlgrenska University Hospital<br>Ekblom, Örjan; Swedish School of Sport and Health Sciences GIH, Department of Physical Activity and Health<br>Arvidsson, D; University of Gothenburg, Center for Health and Performance, Department of Food and Nutrition, and Sport Science<br>Heiland, Emerald G; Swedish School of Sport and Health Sciences GIH, Department of Physical Activity and Health<br>Väisänen, Daniel; Swedish School of Sport and Health Sciences GIH, Department of Physical Activity and Health<br>Bergström, Göran; Sahlgrenska University Hospital, Sahlgrenska Center for Cardiovascular and Metabolic Research, Wallenberg Laboratory; University of Gothenburg, Department of Molecular and Clinical Medicine<br>Ekblom-Bak, Elin; Swedish School of Sport and Health Sciences GIH, Department of Physical activity and Health
**Primary Subject Heading**:	Sports and exercise medicine
Secondary Subject Heading:	Epidemiology, Public health
Keywords:	EPIDEMIOLOGY, PREVENTIVE MEDICINE, PUBLIC HEALTH

SCHOLARONE™
Manuscripts

I, the Submitting Author has the right to grant and does grant on behalf of all authors of the Work (as defined in the below author licence), an exclusive licence and/or a non-exclusive licence for contributions from authors who are: i) UK Crown employees; ii) where BMJ has agreed a CC-BY licence shall apply, and/or iii) in accordance with the terms applicable for US Federal Government officers or employees acting as part of their official duties; on a worldwide, perpetual, irrevocable, royalty-free basis to BMJ Publishing Group Ltd ("BMJ") its licensees and where the relevant Journal is co-owned by BMJ to the co-owners of the Journal, to publish the Work in this journal and any other BMJ products and to exploit all rights, as set out in our licence.

The Submitting Author accepts and understands that any supply made under these terms is made by BMJ to the Submitting Author unless you are acting as an employee on behalf of your employer or a postgraduate student of an affiliated institution which is paying any applicable article publishing charge ("APC") for Open Access articles. Where the Submitting Author wishes to make the Work available on an Open Access basis (and intends to pay the relevant APC), the terms of reuse of such Open Access shall be governed by a Creative Commons licence – details of these licences and which Creative Commons licence will apply to this Work are set out in our licence referred to above.

Other than as permitted in any relevant BMJ Author's Self Archiving Policies, I confirm this Work has not been accepted for publication elsewhere, is not being considered for publication elsewhere and does not duplicate material already published. I confirm all authors consent to publication of this Work and authorise the granting of this licence.

For peer review only

TITLE PAGE

Title of the paper: Correlates of cardiorespiratory fitness in a population-based sample of middle-aged adults – cross-sectional analyses in the SCAPIS study

Authors: Mats Börjesson[1, 2], Örjan Ekblom[3], Daniel Arvidsson[4], Emerald G. Heiland[3, 5], Daniel Väisänen[3], Göran Bergström[6, 7], Elin Ekblom-Bak[3]

[1]Center for Health and Performance, Department of Molecular and Clinical Medicine, Sahlgrenska Academy, Gothenburg University, Sweden.

[2]Dept MGA, Sahlgrenska University Hospital, Region Västra Götaland, Gothenburg, Sweden

[3]Department of Physical Activity and Health, The Swedish School of Sport and Health Sciences, Stockholm, Sweden.

[4]Center for Health and Performance, Department of Food and Nutrition and Sport Science, Faculty of Education, University of Gothenburg.

[5]Department of Surgical Sciences, Medical Epidemiology, Uppsala University, Uppsala, Sweden.

[6]Department of Molecular and Clinical Medicine, Institute of Medicine, Sahlgrenska Academy, University of Gothenburg, Gothenburg, Sweden.

[7]Department of Clinical Physiology, Sahlgrenska University Hospital, Region Västra Götaland, Gothenburg, Sweden.

Corresponding author: Elin Ekblom-Bak, The Swedish School of Sport and Health Sciences, Department of Physical Activity and Health, Stockholm, Sweden. Box 5626, S-114 56 Stockholm, Sweden. Phone: +46812053861. Email: eline@gih.se

For peer review only - http://bmjopen.bmj.com/site/about/guidelines.xhtml

ABSTRACT

Objectives: This study aimed to identify main sex-specific correlates of cardiorespiratory fitness (CRF) in a population-based, urban sample of Swedish adults.

Design: Cross-sectional.

Setting: Multi-site study at university hospitals, data from the Gothenburg site.

Participants: A total of 5,308 participants (51% women, aged 50–64 years) with a valid estimated VO_2max, from submaximal cycle test, in the Swedish CArdioPulmonary bioImage study, SCAPIS, were included.

Primary and secondary outcomes: A wide range of correlates were examined including (a) sociodemographic and lifestyle behaviours, (b) perceived health, anthropometrics, and chronic conditions, and (c) self-reported as well as accelerometer-derived physical activity and sedentary behaviours. Both continuous levels of estimated VO_2max as well as odds ratios (OR) and confidence intervals (CI) of low VO_2max (lowest sex-specific tertile) were reported.

Results: In multivariable regression analyses, higher age, being born abroad, short education, high waist circumference, poor perceived health, high accelerometer-derived time in sedentary, and low in vigorous physical activity, as well as being passive commuter, correlated independently and significantly with low VO_2max in both men and women (OR range 1.31 to 9.58). Additionally in men, financial strain and being an ex-smoker associated with higher odds for low VO_2max (OR 2.15; 95% CI 1.33-3.48 and 1.40; 1.09-1.80), while constant stress with lower odds (0.61; 0.43-0.85). Additionally in women, being a regular smoker associated with lower odds for low VO_2max (0.64; 0.45-0.92). **Conclusions:** The present study provides important reference material on CRF and correlates of CRF in a general middle-aged population, which can be valuable for future research, clinical practice, and public health work. If relations are causal, increased knowledge about specific sub-groups will aid in the development of appropriate, targeted interventions.

For peer review only - http://bmjopen.bmj.com/site/about/guidelines.xhtml

Strengths and limitations of this study

- The large population-based sample with estimated VO_2max from a submaximal test and accelerometer-measured physical activity provides the power required to analyse multiple relevant correlates of CRF.
- Several high-risk sub-groups with significantly lower estimated VO_2max were identified.
- However, the cross-sectional design of the study prevents any causal inference, and we have no information on any genetic contribution to variation between sub-groups of correlates.
- Although based on a large sample from the general population, participants included were younger, leaner, more physically active, and had more often a university degree compared to excluded participants.

Keywords Cardiorespiratory fitness, VO_2max, population-based, correlates, SCAPIS, accelerometer-derived physical activity, reference material

WORD COUNT: 3405 words

For peer review only

BACKGROUND

Levels of cardiorespiratory fitness (CRF), mainly determined by recent levels of moderate-to-vigorous physical activity (MVPA) and genetics (1), is important for aerobic performance and engagement in daily exercise activities. It is also a strong predictor of several non-communicable diseases and all-cause mortality (2-4). Unfortunately, data indicate a decline in CRF on population levels over the last decades (5, 6). CRF is typically lower in older ages (7), in individuals with shorter education (8), daily smokers, and obese persons, yet higher in those engaging regularly in leisure time physical activity (PA) or exercise (9). However, a broader knowledge of key determinants for CRF level is scarce, especially from population-based samples. For example, whether and how CRF levels vary by psychosocial factors and types of domain-specific PA behaviour are not fully elucidated or show conflicting results (9, 10). Moreover, previous studies of PA correlates on CRF are often limited by the use of self-reported PA (11). Also, it is still not fully explored whether and how the age-related lower levels of CRF are associated with less exercise performed, or instead is due to age-related changes in key physiological capacities itself (12). Previous studies have mainly been based on selected samples, consisting of smaller cohorts (n<2000), which limits the power for comparisons between different sub-groups (9). More detailed knowledge of current correlates in unselected samples is needed to understand variations of CRF in the population, not only for the development of targeted assessments of CRF in the population but also the generation of appropriate interventions.

The aim of the present study is to identify main sex-specific correlates of CRF in a population-based, urban sample of 50–64-year-old men and women, including a wide range of correlates: (a) sociodemographic factors and lifestyle behaviours, (b) perceived health, anthropometrics, and chronic conditions, and (c) self-reported as well as accelerometer-derived PA and sedentary behaviours. A secondary aim is to study whether the commonly

For peer review only - http://bmjopen.bmj.com/site/about/guidelines.xhtml

reported lower CRF with older age might be influenced by level of accelerometer-derived sedentary time and/or vigorous PA.

METHODS

Data were retrieved from The Swedish CArdioPulmonary bioImage Study (SCAPIS) (13). In 2012, a comprehensive pilot study was conducted and in 2013-2018 the full SCAPIS study was carried out in collaboration with six Swedish university hospitals (Gothenburg, Linköping, Malmö/Lund, Stockholm, Umeå, and Uppsala), aiming to recruit 5000 individuals (men and women, 50-64 years) from their respective municipality. Data collection took place over two to three occasions within a 2-week period. The participants underwent extensive imaging and functional studies of the heart, lungs, and metabolism, filled in an extensive questionnaire, and wore an accelerometer during seven days. At each test-site, there was a possibility to include site-specific measurements. At the Gothenburg site, a submaximal cycle test was performed to estimate VO_2max. In the pilot study, a total of 2,243 participants were recruited from the census register, and 1,111 (49.5%) agreed to participate. In the full study in Gothenburg, 12,109 were recruited and 6,266 (52%) participated. Thus, a total of 7,377 were eligible and 5,308 participants (72%) provided valid estimated VO_2max. The study was approved by the ethics board (Dnr 2010-228-31M, Dnr 2011-449-32M, and Dnr 638-16) and adheres to the Declaration of Helsinki. All participants provided written informed consent.

Assessment of cardiorespiratory fitness

CRF was assessed as estimated VO_2max using the Ekblom-Bak submaximal cycle ergometer test (14). The test uses the difference in heart rate response between 4 minutes cycling on a lower, standardised work rate (30 watts) and 4 minutes cycling on a higher, individually chosen work rate (aiming at 13-14 at the Borg RPE scale). Pedal frequency is held constant at

For peer review only - http://bmjopen.bmj.com/site/about/guidelines.xhtml

both work rates (60 revolution per minute) and heart rate is measured as the mean during the last minute at each work rate. The heart rate difference is then related to the increase in work rate between the two work rates, and introduced in sex-specific validated algorithms for estimation of VO_2max. The test has shown high validity in reference to direct measurement of VO_2max in this age-group (r^2=0.84, standard error of estimate 0.33 L$\square$min^{-1}) (14). Submaximal testing of CRF has been shown to predict all-cause mortality and cardiovascular disease (CVD) event in large, contemporary populations (15, 16), which highlights the possibility to use submaximal testing in larger population samples. To minimise well-known errors with submaximal testing, participants were requested to refrain from consuming a heavy meal or drinking coffee during the hours before the test. A priori exclusion criteria included symptoms of on-going infections, known unstable CVD, electrocardiography patterns indicative of cardiac disease, use of beta-blockers, weight>125 kg, or resting heart rate of >100 bpm (n=466). Moreover, participants did not take part in the testing if refrained from participating in testing (n=1209), did not receive a valid fitness test due to lack of test criteria fulfilment, did not fulfil the test, or malfunction of heart rate monitors or the ergometer (n=394).

Assessment of anthropometrics and chronic conditions

Measurements of weight, height, and waist circumference were assessed during the first visit to the test centre. Body mass index (BMI) was calculated (kg$\square$m^{-2}), and waist circumference was measured at the midpoint between the top of the iliac crest and the lower margin of the lowest palpable rib. Prevalent depressive symptoms lasting 2 weeks or more during the last 12 months were self-reported. Prevalent chronic conditions were defined as reporting diagnosed disease or surgical treatment for CVD (i.e., myocardial infarction, angina pectoris, stroke,

For peer review only - http://bmjopen.bmj.com/site/about/guidelines.xhtml

congestive heart failure, atrial fibrillation), hypertension, lung disease, dyslipidaemia, diabetes, rheumatic disease, and/or cancer.

Assessment of physical activity pattern

Self-reported PA included commuting habits, physical working situation, exercise, leisure time sitting, and total PA (see Additional File 1). Sedentary behaviour and PA were also derived from tri-axial accelerometers (ActiGraph model GT3X and GT3X+, ActiGraph LCC, Pensacola, FL, USA). Participants were instructed to wear the accelerometer on an elastic belt over the right hip during all waking hours for at least 7 consecutive days, except during water-based activities. ActiLife v.6.10.1 software was used to initialise the accelerometers and to download and process the collected data. The accelerometer recorded raw data (30 Hz) from all three axes, which were combined into a resulting vector, and extracted as 60 second epochs using a low frequency extension filter. Sedentary was defined as <200 counts per minute (cpm) (17), low intensity PA as 200-2689 cpm, moderate intensity PA as 2690-6166 cpm, and vigorous PA (VPA) as ≥6167 cpm (18). Non-wear time was defined as ≥60 consecutive minutes with no movement (0 cpm), with allowance for maximum 2 minutes of counts between 0 and 200 cpm. Wear time was calculated as 24 hours minus non-wear time. A minimum of 600 minutes of valid daily wear time for at least 4 days was required for inclusion (19). Prolonged sedentary time was defined as ≥20 minutes of cpm below 200, with no allowance for interruption above threshold.

Assessment of sociodemographic, lifestyle, and perceived symptoms and health

Marital status, country of birth, educational level, employment status, financial strain, and smoking status were self-reported and grouped according to definitions presented in Table 1. Pack-years of smoking was derived by multiplying the average number of cigarettes smoked

For peer review only

For peer review only - http://bmjopen.bmj.com/site/about/guidelines.xhtml

per day by the number of years smoking, divided by the number of cigarettes per package. The 10-item screening tool AUDIT was used to define alcohol abuse (20), and categorised into three groups, “No/Low”, “Moderate”, and “High”, according to the AUDIT score 0-7, 8-15, and >15 for men, and 0-5, 6-13, and >13 for women. Sleep, stress, perceived control at work and in leisure-time, as well as general health, were also self-reported and grouped as presented in Table 2.

Patient and public involvement

No patient involved.

Statistical analysis

CRF was skewed (Kolmogorov-Smirnov Test), hence, descriptive data are presented as medians and 95% confidence intervals (CIs). Low CRF was defined as the lowest sex-specific tertile (cut-off 27.75 mL□min^{-1}□kg^{-1} for women and 33.65 mL□min^{-1}□kg^{-1} for men). Odds ratios (ORs) with 95% confidence intervals (CIs) for low CRF in relation to the sub-groups of the correlates were calculated using binary logistic regression modeling adjusting for sex and age. Interaction between sex and each correlate was assessed by adding an interaction term to the model, and significant interactions were defined as $p<0.05$ for the interaction term. To study independent associations of correlates with low CRF, all correlates not displaying multicollinearity (Spearman's $\rho>0.6$) were entered in a backward logistic multivariable regression model, with low CRF as the dependent variable. To study whether the relationship between age-group and estimated VO_2max was modified by vigorous PA (VPA) or prolonged sedentary, general linear modeling with post-hoc analyses was used including an interaction term for age-group*VPA or age-group*prolonged sedentary, and adjusting for sex. The VPA variable was divided into four groups: 0 min/week, 0 to 37.5 min/week (half of the

For peer review only

For peer review only - http://bmjopen.bmj.com/site/about/guidelines.xhtml

recommended VPA level), >37.5 to <75 min/week (the recommended VPA level), and ≥ 75 min/week (21). For sedentary behaviour, the quartiles from Table 3 were used. Statistical analyses were performed using SPSS (26.0, 2019, SPSS Inc., Chicago IL) and R (R version 4.1.1) with R packages ggplot2 and ggeffects.

RESULTS

Of the total 7,377 participants eligible for exercise testing in the study, 5,308 provided valid estimates for VO_2max. Participants included, compared with participants not included (n=2,069), were more often women (54% vs 51%, p=0.040), younger (age 57.2 vs 58.2 years, p<0.001), had lower BMI (26.5 vs 27.7 kg□m^{-2}, p<0.001), had more often university degree (47% vs 35%, p<0.001), and more often reported to be weekly regular exercisers (53% vs 40%, p<0.001). Additionally, among included participants, 14% were regular smokers, 2.3% had established CVD, 17% had hypertension, 11% had dyslipidaemia, and 2.5% had diabetes (all self-reported).

Associations between CRF and the different correlates are presented in Tables 1 to 3 and Appendix Figure 1 and 2, with sex-specific analyses presented in Appendix Table 1. The results can be summarized as follows:

- Sociodemographic factors and Lifestyle behaviours (Table 1): Women, older age, divorced/single/widowed, participants not born in Sweden, lower education, employment<50% of full-time/retired/pension/unemployed, with financial strain, or being regular and former smokers had lower estimated CRF and higher OR for having low CRF, compared with their counterparts. Older men (60-64 years) were more likely to have lower CRF than older women (p=0.045), with no other interactions seen between sex and the other sociodemographic variables. For lifestyle factors, there were significant interactions between

For peer review only

For peer review only - http://bmjopen.bmj.com/site/about/guidelines.xhtml

sex and smoking, such that men being regular smokers/sometimes smokers, and with more pack-years in life, were more likely to have low CRF (p=0.004 and p=0.008) compared with smoking women.

Table 1. Estimated VO_2max (mL□min^{-1}□kg^{-1}) in association with sociodemographic factors and lifestyle behaviour correlates.

		**Estimated VO_2max**	**Low estimated VO_2max**	
	n	Median (95% CI)	% within subgroup	OR[C] (95% CI)
**Sex**				
Men	2590	36.2 (35.8-36.5)[a]	na[d]	na
Women	2718	30.4 (30.1-30.7)	na	na
**Age**				
50-54 years	1881	34.9 (34.5-35.1)[b]	24%	1 (ref)
55-59 years	1812	33.7 (33.2-34.0)	32%	1.46 (1.27-1.69)
60-64 years	1615	31.5 (31.0-31.8)	45%	2.57 (2.23-2.97)
**Marital status**				
Married/Cohabitation	3766	33.8 (33.6-34.1)[a]	31%	1 (ref)
Divorced/Single/Widower	1461	32.1 (31.7-32.6)	38%	1.37 (1.21-1.56)
**Born in Sweden**				
Yes	4341	33.7 (33.4-33.9)[a]	31%	1 (ref)
No	923	32.0 (31.3-32.5)	43%	1.75 (1.51-2.03)
**Educational level**				
University degree	2480	34.3 (33.9-34.6)[b]	26%	1 (ref)
High school/Vocational education	2215	32.8 (32.4-33.2)	38%	1.85 (1.63-2.10)
Elementary school	555	31.6 (30.8-32.4)	50%	2.82 (2.32-3.42)
**Employment**				
Working, ≥ 50% of full-time	4342	33.9 (33.6-34.1)[b]	30%	1 (ref)
Retired	231	30.3 (29.8-31.4)	53%	1.55 (1.17-2.05)
Disability pension/Sickness pension/Sick leave	299	29.3 (28.4-30.2)	54%	2.60 (2.05-3.31)
Unemployed/Student	365	31.3 (30.9-32.2)	45%	1.88 (1.51-2.35)
**Financial strain**				
No	4759	33.6 (33.4-33.9)[a]	32%	1 (ref)
Yes	406	30.1 (29.6-30.7)	51%	2.51 (2.04-3.10)

For peer review only

**Smoking habits**				
Never smoker	2463	34.6 (34.2-34.9)[b]	29%	1 (ref)
Former smokers	2015	32.3 (31.9-32.6)	38%	1.38 (1.21-1.57)
Regular smoker/Sometimes	716	32.2 (31.8-32.9)	39%	1.61 (1.35-1.92)
**Pack-years**				
Never smokers (0)	2463	34.6 (34.2-34.9)[b]	29%	1 (ref)
Former smokers, low (<15 pack-years)	1220	33.0 (32.6-33.7)	30%	1.00 (0.86-1.17)
Former smokers, heavy (≥15 pack-years)	795	31.1 (30.5-31.6)	49%	2.17 (1.83-2.56)
Current smokers, low (<15 pack-years)	226	33.6 (32.4-34.8)	30%	1.11 (0.82-1.50)
Current smokers, heavy (≥15 pack-years)	490	31.8 (31.1-32.3)	42%	1.88 (1.53-2.30)
**Alcohol use**				
No/Low	3948	33.7 (33.4-34.0)	30%	1 (ref)
Moderate	639	33.3 (33.0-33.9)	34%	1.26 (1.05-1.51)
High	33	32.5 (30.0-33.9)	52%	3.06 (1.51-6.19)

[a] Significantly different from other sub-group (Mann–Whitney U test, p<0.0001)

[b] Kruska-Wallis ANOVA p<0.0001

[c] Adjusted for sex and age, comparing the lowest sex-specific tertile of estimated VO_2max with the two higher tertiles

[d] % in low estimated VO_2max and OR not applicable for comparison between men and women, as tertiles were sex-specific.

For peer review only

- Perceived health, anthropometrics, and chronic conditions (Table 2 and Appendix Figure 1):

Participants reporting poor sleep, high stress, low control at work, low perceived degree of control in life, poorer general health, having higher BMI, higher waist circumference, prevalence of depression symptoms, and higher number of chronic conditions had lower estimated CRF. Women with low perceived degree of control in life were more likely to have low CRF than their male counterparts (p=0.004). Higher BMI and higher waist circumference were more strongly associated with low CRF in women than in men (p=0.001 and p=0.041).

Table 2. Estimated VO_2max (mL□min^{-1}□kg^{-1}) in association with perceived health, anthropometrics, and chronic condition correlates.

		**Estimated VO_2max**	**Low estimated VO_2max**	
	n	Median (95% CI)	% within subgroup	OR[C] (95% CI)
**Sleep**				
Very well/well	2401	34.2 (33.9-34.5)[a]	31%	1 (ref)
Rather well/Badly/Very badly	2835	32.6 (32.2-32.9)	35%	1.22 (1.09-1.38)
**Stress**				

For peer review only - http://bmjopen.bmj.com/site/about/guidelines.xhtml

No stress/Some stress during last 5 years	4089	33.7 (33.4-33.9)[a]	33%	1 (ref)
Constant stress last 1 to 5 years	1129	32.2 (31.8-32.6)	36%	1.25 (1.08-1.44)
**Control at work**				
Strongly agree/Agree	3846	33.8 (33.6-34.1)[a]	31%	1 (ref)
Neutral/Do not agree/Do not agree at all	1111	32.3 (31.9-32.8)	35%	1.23 (1.06-1.42)
**Control in life**				
Do not agree at all/Do not agree	2976	34.0 (33.7-34.3)[a]	30%	1 (ref)
Neutral/Agree/Strongly agree	2193	32.5 (32.1-32.8)	37%	1.46 (1.29-1.64)
**General health**				
Excellent/Very good	2698	35.0 (34.8-35.3)[b]	23%	1 (ref)
Good	1818	32.5 (32.2-33.0)	40%	2.41 (2.11-2.75)
Somewhat bad/Bad	729	29.4 (28.8-30.1)	56%	4.80 (4.02-5.73)
**BMI with estVO$_2$max in L□min^{-1}**				
<18	11	2.09 (1.79-2.55)[b]	55%	0.65 (0.19-2.29)
18.0-24.9	2025	2.38 (2.35-2.40)	36%	1 (ref)[#]
25.0-29.9	2341	2.75 (2.70-2.80)	33%	2.32 (1.95-2.76)
30.0-34.9	728	2.63 (2.52-2.73)	29%	5.79 (4.30-7.81)
≥35	203	2.38 (2.29-2.49)	29%	16.6 (10.2-27.1)
**Waist circumference**				
Low waist (<88 cm women, <102 cm men)	3191	36.4 (36.2-36.7)[a]	15%	1 (ref)
High waist (≥88 cm women, ≥102cm men)	2114	28.9 (28.6-29.2)	61%	10.2 (8.8-11.7)
**Depression symptoms**				
No	3687	33.9 (33.7-34.2)[a]	32%	1 (ref)
Yes	1495	32.0 (31.6-32.3)	37%	1.33 (1.17-1.51)
**Chronic conditions***				
0	3465	33.9 (33.7-34.3)[b]	30%	1 (ref)
1-2	1705	32.3 (31.9-32.8)	39%	1.38 (1.22-1.57)
=>3	138	31.0 (29.7-32.7)	51%	2.09 (1.48-2.96)
**Cardiovascular disease, Hypertension and/or Lung disease**				
No	4069	33.7 (33.5-34.0)	32%	1 (ref)
Yes	1239	32.0 (31.5-32.5)	37%	1.42 (1.24-1.62)

[a] Significantly different from other sub-group (Mann–Whitney U test, p<0.0001)

[b] Kruska-Wallis ANOVA p<0.0001

[c] Adjusted for sex and age, comparing the lowest sex-specific tertile of estimated VO$_2$max with the two higher tertiles
* Includes Cardiovascular disease, Hypertension, Lung disease, Dyslipidaemia, Diabetes, Rheumatic disease and Cancer

For peer review only

For peer review only - http://bmjopen.bmj.com/site/about/guidelines.xhtml

[#] Sex-specific tertiles based on L/min. Analyses additionally adjusted for weight in kg.

- Self-reported and accelerometer-derived PA pattern (Table 3 and Appendix Figure 2):

Participants engaging in any active commuting had higher CRF compared with those with no active commuting. This was mainly seen among partly- and all-year commuters by bike. Participants with a more strenuous physical working situation, less weekly exercise, lower self-reported total PA level, and high self-reported sitting time had lower CRF. Women reporting higher total PA had lower OR for low CRF (p=0.021) compared with men. Higher accelerometer-derived time spent in sedentary and prolonged sedentary time were associated with lower CRF, whereas more time spent in low intensity PA, moderate PA, and VPA were associated with higher CRF. Women with higher sedentary time were more likely to have lower CRF compared with men (p=0.033).

For peer review only

Table 3. Estimated VO_2max (mL□min^{-1}□kg^{-1}) in association with self-reported and accelerometer-derived physical activity pattern correlates.

		**Estimated VO_2max**	**Low estimated VO_2max**	
	n	Median (95% CI)	% within subgroup	OR[C] (95% CI)
**Self-report**				
**Commuting habits***				
No active commuting	3057	33.1 (32.8-33.4)[b]	37%	1 (ref)
Any active commuting, further divided into:	1738	34.6 (34.2-35.0)	23%	0.47 (0.41-0.54)
Partly active commuter, bike	671	34.8 (34.1-35.5)	20%	0.41 (0.33-0.50)
Partly active commuter, walking	196	30.4 (29.3-31.6)	41%	1.12 (0.83-1.52)
Partly active commuter, mix	19	30.2 (28.8-33.5)	21%	0.45 (0.15-1.38)
All year active commuter, bike	407	38.1 (36.9-39.2)	10%	0.18 (0.13-0.25)
All year active commuter, walking	415	33.0 (32.2-33.4)	31%	0.71 (0.57-0.89)
All year active commuter, mix	30	34.7 (30.6-37.3)	23%	0.51 (0.22-1.22)
**Physical working situation**				
Sedentary to light	3921	33.7 (33.5-34.1)[a]	30%	1 (ref)
Sometimes to frequently heavy	945	32.5 (32.0-33.1)	38%	1.44 (1.24-1.67)
**Exercise habits**				
Never	1384	31.0 (30.7-31.4)[b]	51%	1 (ref)
Irregular	1067	31.3 (30.8-31.7)	43%	0.75 (0.64-0.88)
1-2 times per week	1032	33.5 (33.0-34.1)	27%	0.35 (0.29-0.41)
2-3 times per week	1089	35.2 (34.8-35.7)	20%	0.25 (0.21-0.30)

For peer review only - http://bmjopen.bmj.com/site/about/guidelines.xhtml

>3 times per week	649	38.3 (37.8-38.9)	12%	0.14 (0.11-0.19)
**Total physical activity**				
Sedentary	540	30.0 (29.1-30.5)[b]	63%	1 (ref)
Light	2366	31.5 (31.2-31.9)	42%	0.39 (0.32-0.48)
Moderate	1613	34.9 (34.6-35.4)	21%	0.14 (0.11-0.18)
Regular exercise/training	669	38.6 (38.0-39.1)	9%	0.06 (0.05-0.09)
**Leisure time sitting**				
Q1; <4 h per day	1121	34.1 (33.5-34.7)	28%	1 (ref)
Q2; 4-6 h per day	1039	33.6 (33.0-34.2)	32%	1.24 (1.02-1.49)
Q3; 6-8.5 h per day	925	34.0 (33.5-34.5)	29%	1.11 (0.91-1.36)
Q4; >8.5 h per day	958	33.7 (33.3-34.2)	32%	1.31 (1.08-1.59)
**% of time spent in Sedentary**				
Q1; <47%	1267	34.1 (33.5-34.5)[b]	26%	1 (ref)
Q2; 47%-54%	1265	33.6 (33.1-34.1)	28%	1.19 (0.99-1.42)
Q3; 54%-61%	1266	33.4 (33.0-33.7)	33%	1.52 (1.27-1.81)
Q4; >61%	1267	33.0 (32.3-33.3)	43%	2.44 (2.05-2.91)
**% of time spent in prolonged Sedentary (of total wear time)**				
Q1; <13%	1266	34.1 (33.6-34.5)[b]	25%	1 (ref)
Q2; 13%-19%	1267	33.5 (33.0-34.0)	28%	1.14 (0.96-1.37)
Q3; 19%-26%	1265	33.5 (33.1-34.0)	35%	1.60 (1.35-1.91)
Q4; >26%	1267	32.7 (32.2-33.1)	42%	2.18 (1.83-2.60)
**% of time spent in Light physical activity**				
Q1; <33%	1267	33.7 (33.2-34.0)	39%	1 (ref)
Q2; 33%-40%	1266	33.3 (32.9-33.7)	34%	0.78 (0.66-0.92)
Q3; 40%-46%	1265	33.5 (33.1-34.0)	29%	0.60 (0.51-0.72)
Q4; >46%	1267	33.2 (32.8-33.8)	28%	0.56 (0.47-0.66)
**% of time spent in Moderate physical activity**				
Q1; <3.8%	1267	31.6 (31.6-32.5)[b]	44%	1 (ref)
Q2; 3.8%-5.3%	1265	33.3 (32.7-33.7)	32%	0.62 (0.52-0.73)
Q3; 5.3%-7.2%	1266	33.8 (33.3-34.1)	29%	0.52 (0.44-0.62)
Q4; >7.2%	1267	34.9 (34.3-35.3)	26%	0.44 (0.37-0.52)
**% of time spent in Vigorous physical activity**				
Q1;<0.02%	1266	30.5 (30.1-31.0)[b]	49%	1 (ref)
Q2; 0.02%-0.1%	1266	31.7 (31.3-32.1)	43%	0.80 (0.69-0.94)
Q3; 0.1%-0.6%	1267	34.3 (34.0-34.7)	23%	0.34 (0.28-0.40)
Q4; >0.6%	1266	36.7 (36.3-37.1)	16%	0.21 (0.18-0.26)

[a] Significantly different from other sub-group (Mann–Whitney U test, p<0.0001)

[b] Kruska-Wallis ANOVA p<0.0001

For peer review only

For peer review only - http://bmjopen.bmj.com/site/about/guidelines.xhtml

* Analysed both as comparing No active commuting and Any active commuting, as well as further dividing any active commuting into; Partly commuting, defined as going by bike or walking at least one season out of four; All year commuting, defined as going by bike or walking all year around. Partly and all year commuting "walking" or "bike" were defined as the majority of seasons commuted with that mode of active commuting. Mix was defined when the seasons of active commuting were spent equally between the two modes.

In the multivariable regression model (Figure 1), older age-group, being born abroad, having lower education, high waist circumference, poorer general health, and more time spent sedentary, were associated with a higher OR for low CRF in both men and women. More time spent in VPA and commuting by bike (partly and all-year) were associated with a lower OR. Specifically in men, financial strain and being an ex-smoker were associated with higher OR, and constant stress with a lower OR, for a low CRF. Specifically in women, being a regular smoker was associated with lower OR for low CRF, with no significant associations with financial strain or stress.

Insert **Figure 1** here.

In Figure 2, higher levels of VPA were more common in younger age-groups (left part of figure). However, higher levels of VPA associated with significantly higher CRF in all age-groups (p<0.001). There was no overall interaction between VPA and age-group (p=0.372). Stratified analyses showed that there was no significant difference in CRF between participants with 37.5-75 minutes and ≥75 minutes of VPA per week in the oldest age-group (p=1.00). Also, participants aged 50-54 years and 55-59 years with 0 min/week of VPA had similar CRF (p=0.305 for differences between groups), and so did participants aged 55-59 and 60-64 years with 37.5 to 75 min/week (p=1.000 for differences between groups). In the right part of Figure 2, higher levels of time spent sedentary were associated with lower CRF in all age-groups. There was no overall interaction effect between sedentary time and age-group

For peer review only

For peer review only - http://bmjopen.bmj.com/site/about/guidelines.xhtml

(p=0.596). Stratified analyses showed that there was some overlapping between age-groups and time sedentary for CRF.

Insert **Figure 2** here.

DISCUSSION

The present study identified multiple correlates of CRF in a large, population-based sample of middle-aged, urban Swedish adults from the SCAPIS study. The results confirm some previously documented correlates, but also adds important novel aspects, including correlates of depression symptoms, commuting habits, and accelerometer-derived PA. Some of the most interesting correlates are discussed, as follows:

Firstly, the strong, positive association between CRF and length of education confirms previous results (8-10), while similar associations with being born in Sweden, being employed, and having an economic buffer, adds novel information regarding sociodemographic aspects. These associations may largely be mediated by level of VPA. Individuals in these sub-groups have previously been shown to engage in higher levels of daily VPA compared with their counterparts (22), and a strong agreement between socioeconomic factors and both PA and CRF has been reported (8). The association between lower CRF in groups with lower socioeconomic status is also in line with higher observed incidence of cardiovascular, mood-related disorders, and premature mortality in lower socioeconomic strata compared with those of higher socioeconomic status (23). Also, in a recent paper on risk of severe COVID-19, the associations between socioeconomic factors such as educational level, income, and occupational group and severe COVID-19 risk, were largely mediated by the CRF level of the participants (24).

For peer review only

For peer review only - http://bmjopen.bmj.com/site/about/guidelines.xhtml

294 Secondly, participants reporting more physically demanding work had in general a lower
295 CRF, compared with more sedentary workers. While this is in line with previous reports from
296 the Swedish working population (25), this may at first be counterintuitive as higher
297 occupational PA could be considered as a "work-out" contributing to health benefits and
298 higher CRF. However, although more physically demanding occupations induce a higher
299 mean intensity compared with more sedentary occupations, the relative intensity of "active
300 jobs" have been shown to be maybe too low to improve VO_2max (26). Rather, the higher
301 mean intensity may be tiring enough for the individual, which could lead to lower levels of
302 fitness-enhancing leisure time VPA (22). This, together with a more pronounced increase in
303 body weight in blue-collar (more active) occupational groups (27), may contribute to the
304 general lower CRF levels in these groups. Also, the mean intensity during work has been
305 associated with lower heart rate variability and a higher heart rate the following night (26).
306 This suggests an induced imbalance in the autonomic cardiac modulation, which may be one
307 potential underlying mechanism for the detrimental effects on health proposed for
308 occupational PA, also called the "physical activity paradox" (28). Thus, differences in CRF
309 may partly contribute to the observed paradox.

310

311 Thirdly, we could confirm the established relation between self-reported total and leisure-time
312 PA and CRF (9), however, expanding on knowledge regarding CRF in active commuters.
313 Any active commuting was associated with higher CRF, however, the higher CRF was mainly
314 driven by partly and all-year commuters by bike. This is line with higher levels of VPA
315 shown in active commuters (22). Although the present study cannot clarify whether it was the
316 active commuting per se that contributed to higher CRF levels, mean intensity during normal
317 commuting in habitual cycle commuters has been shown to be mainly constituted of the lower
318 end of vigorous intensity (29) for longer activity bouts (>10 minutes). This then often adds up

For peer review only

to recommended levels of 150 minutes per week (30). Thus, regular active commuting by bike requires an at least moderate or even high CRF.

Fourthly, previous data on correlates between CRF and accelerometer-assessed PA patterns are scarce and inconclusive (9). We found that higher accelerometer-derived estimates of the different components of the PA pattern provided a strong association between CRF level and time in sedentary (negative association), and MPA and VPA (positive associations). Interestingly, both time in sedentary and in VPA remained significantly associated with CRF in the multivariable regression model, which implies separate importance of these intensities for CRF level.

Finally, previous research is unclear about whether higher PA level would ameliorate lower CRF levels among older individuals (9). The cross-sectional analyses in our study showed that older individuals had lower CRF than younger, with strong associations between higher VPA and less time spent sedentary, and CRF in all age-groups. More time in VPA may be required for older individuals in order to maintain a certain CRF, as the mean estimated VO_2max in individuals 60-64 years old engaging in 37 minutes or more in VPA per week was overlapping with the VO_2max in individuals 50-54 years old doing less than 37 minutes of VPA per week. It is also possible that relations are reversed or at least bi-directional, i.e., that a higher CRF will enable an individual to be physically active, including higher intensity activities. Indeed, previous studies have shown that those being active at higher age, have superior CRF compared with less active peers (12). Age-related medico-physiologic changes, possibly affecting the possibility to be active, such as loss of muscle mass or increasing concomitant disease and medications, may affect the intensity of the VPA performed (7).

For peer review only - http://bmjopen.bmj.com/site/about/guidelines.xhtml

For peer review only

Strengths and limitations

The cross-sectional design of the study prevents any causal inference and we cannot rule out reverse causality. We have no information on any genetic contribution to variation between sub-groups of correlates. Although we have a large sample from the general population, those who participated in the fitness testing did differ from the source population. Therefore, translation of the results to populations that are less healthy and active and of other socioeconomic status (e.g., lower education), should be done with caution. Further, the large sample size with estimated CRF from a submaximal test and accelerometer-measured PA provided the power required to analyse multiple correlates relevant for CRF. It is unknown if regular bicycling affects validity of a cycle ergometer test. Local adaptations to the thigh and gluteal muscles from regular cycling may alter systemic circulatory response to standard cycling exercise. If so, the finding that regular bike commuters had higher CRF may in part be ascribed to varying validity.

CONCLUSIONS

This study in a large sample of middle-aged men and women delineates multiple modifiable as well as non-modifiable correlates of CRF. In addition, we identified specific sub-groups as potential high-risk groups for CRF-related disorders, which constitute targets for behavioural change to increase CRF, if relations are causal. These groups include individuals with higher age, being born abroad, lower education, high waist circumference, poor perceived health, high accelerometer-derived time in sedentary and low in VPA, as well as passive commuters. Across all age-groups, higher VPA and less time spent sedentary, were associated with a higher CRF. Although having limited possibilities for causal inference, the present study provides important reference material of CRF and correlates in a general middle-aged population, which can be valuable for future research, clinical practice, and public health

For peer review only - http://bmjopen.bmj.com/site/about/guidelines.xhtml

work. Specifically, increased knowledge about specific sub-groups will aid in the personalised prescription of exercise in health care. In light of the higher prevalence of low CRF in later years (5), a great challenge remains in implementing effective interventions to support health in identified parts of the population.

DECLARATIONS

Funding

The main funding body of The Swedish CArdioPulmonary bioImage Study (SCAPIS) is the Swedish Heart and Lung Foundation (N/A). The study is also funded by the Knut and Alice Wallenberg Foundation (N/A), the Swedish Research Council (N/A) and VINNOVA (N/A) the University of Gothenburg and Sahlgrenska University Hospital (N/A), Karolinska Institutet and Stockholm county council (N/A), Linköping University and University Hospital (N/A), Lund University and Skåne University Hospital (N/A), Umeå University and University Hospital (N/A), Uppsala University and University Hospital (N/A). Author MB is supported by ALF grants from Västra Götalandsregionen (ALFGBG-965249) and a grant from the Heart and Lung Foundation (no 20210270). Author ÖE, EH, and EEB were funded by grants from Skandia Risk & Hälsa (N/A). Author GB was supported by Heart and Lung Foundation (20180324), the Swedish Research Council (2019-01140), and LUA/ALF (ALFGBG-718851).

Competing interests

The authors declare that they have no competing interests.

For peer review only

Authors' contributions

All authors contributed to the conception or design of the work. Mats Börjesson, Örjan Ekblom, Daniel Arvidsson, Daniel Väisänen, Elin Ekblom-Bak contributed to the acquisition, analysis, or interpretation of data for the work. Mats Börjesson, Örjan Ekblom, Emerald G. Heiland, Göran Bergström, Elin Ekblom-Bak drafted the manuscript or substantively revised it. All authors revised it critically for important intellectual content. All authors critically revised the manuscript, gave final approval, and agreed to be accountable for all aspects of the work in ensuring that questions related to the accuracy or integrity of any part of the work are appropriately investigated and resolved. The corresponding author, Elin Ekblom-Bak, is the manuscripts guarantor and attests that all listed authors meet authorship criteria, that no others meeting the criteria have been omitted and take the full responsibility for the overall content.

Availability of data and material

Due to the nature of the sensitive personal data and study materials, they cannot be made freely available. However, by contacting the corresponding author or study organisation (www.scapis.org), procedures for sharing data, analytic methods, and study materials for reproducing the results or replicating the procedure can be arranged following Swedish legislation.

Ethics approval

The study was approved by the ethics board (Dnr 2010-228-31M, Dnr 2011-449-32M, and Dnr 638-16) and adheres to the Declaration of Helsinki. All participants provided written informed consent.

For peer review only - http://bmjopen.bmj.com/site/about/guidelines.xhtml

REFERENCES

1. Bouchard C, An P, Rice T, Skinner JS, Wilmore JH, Gagnon J, et al. Familial aggregation of VO(2max) response to exercise training: results from the HERITAGE Family Study. Journal of applied physiology. 1999;87(3):1003-8.

2. Kodama S, Saito K, Tanaka S, Maki M, Yachi Y, Asumi M, et al. Cardiorespiratory fitness as a quantitative predictor of all-cause mortality and cardiovascular events in healthy men and women: a meta-analysis. Jama. 2009;301(19):2024-35.

3. Kandola A, Ashdown-Franks G, Stubbs B, Osborn DPJ, Hayes JF. The association between cardiorespiratory fitness and the incidence of common mental health disorders: A systematic review and meta-analysis. J Affect Disord. 2019;257:748-57.

4. Laukkanen JA, Isiozor NM, Kunutsor SK. Objectively Assessed Cardiorespiratory Fitness and All-Cause Mortality Risk: An Updated Meta-analysis of 37 Cohort Studies Involving 2,258,029 Participants. Mayo Clinic proceedings. 2022;97(6):1054-73.

5. Ekblom-Bak E, Ekblom O, Andersson G, Wallin P, Soderling J, Hemmingsson E, et al. Decline in cardiorespiratory fitness in the Swedish working force between 1995 and 2017. Scandinavian journal of medicine & science in sports. 2019;29(2):232-9.

6. Lamoureux NR, Fitzgerald JS, Norton KI, Sabato T, Tremblay MS, Tomkinson GR. Temporal Trends in the Cardiorespiratory Fitness of 2,525,827 Adults Between 1967 and 2016: A Systematic Review. Sports Med. 2019;49(1):41-55.

7. Katzel LI, Sorkin JD, Fleg JL. A comparison of longitudinal changes in aerobic fitness in older endurance athletes and sedentary men. J Am Geriatr Soc. 2001;49(12):1657-64.

8. Lindgren M, Borjesson M, Ekblom O, Bergstrom G, Lappas G, Rosengren A. Physical activity pattern, cardiorespiratory fitness, and socioeconomic status in the SCAPIS pilot trial - A cross-sectional study. Prev Med Rep. 2016;4:44-9.

For peer review only - http://bmjopen.bmj.com/site/about/guidelines.xhtml

9. Zeiher J, Ombrellaro KJ, Perumal N, Keil T, Mensink GBM, Finger JD. Correlates and Determinants of Cardiorespiratory Fitness in Adults: a Systematic Review. Sports Med Open. 2019;5(1):39.

10. Ombrellaro KJ, Perumal N, Zeiher J, Hoebel J, Ittermann T, Ewert R, et al. Socioeconomic Correlates and Determinants of Cardiorespiratory Fitness in the General Adult Population: a Systematic Review and Meta-Analysis. Sports Med Open. 2018;4(1):25.

11. Ekblom O, Ekblom-Bak E, Bolam KA, Ekblom B, Schmidt C, Soderberg S, et al. Concurrent and predictive validity of physical activity measurement items commonly used in clinical settings--data from SCAPIS pilot study. BMC Public Health. 2015;15:978.

12. Hawkins S, Wiswell R. Rate and mechanism of maximal oxygen consumption decline with aging: implications for exercise training. Sports Med. 2003;33(12):877-88.

13. Bergstrom G, Berglund G, Blomberg A, Brandberg J, Engstrom G, Engvall J, et al. The Swedish CArdioPulmonary BioImage Study: objectives and design. J Intern Med. 2015;278(6):645-59.

14. Bjorkman F, Ekblom-Bak E, Ekblom O, Ekblom B. Validity of the revised Ekblom Bak cycle ergometer test in adults. Eur J Appl Physiol. 2016;116(9):1627-38.

15. Laukkanen JA, Kunutsor SK, Yates T, Willeit P, Kujala UM, Khan H, et al. Prognostic Relevance of Cardiorespiratory Fitness as Assessed by Submaximal Exercise Testing for All-Cause Mortality: A UK Biobank Prospective Study. Mayo Clinic proceedings. 2020;95(5):867-78.

16. Ekblom-Bak E, Ekblom B, Soderling J, Borjesson M, Blom V, Kallings LV, et al. Sex- and age-specific associations between cardiorespiratory fitness, CVD morbidity and all-cause mortality in 266.109 adults. Prev Med. 2019;127:105799.

For peer review only

For peer review only - http://bmjopen.bmj.com/site/about/guidelines.xhtml

17. Aguilar-Farias N, Brown WJ, Peeters GM. ActiGraph GT3X+ cut-points for identifying sedentary behaviour in older adults in free-living environments. J Sci Med Sport. 2014;17(3):293-9.

18. Sasaki JE, John D, Freedson PS. Validation and comparison of ActiGraph activity monitors. J Sci Med Sport. 2011;14(5):411-6.

19. Migueles JH, Cadenas-Sanchez C, Ekelund U, Delisle Nystrom C, Mora-Gonzalez J, Lof M, et al. Accelerometer Data Collection and Processing Criteria to Assess Physical Activity and Other Outcomes: A Systematic Review and Practical Considerations. Sports Med. 2017;47(9):1821-45.

20. Babor TF, de la Fuente JR, Saunders J, Grant M. AUDIT. The Alcohol Use Disorders Identification Test. Guidelines for use in primary health care. World Health Organization. 2001.

21. Bull FC, Al-Ansari SS, Biddle S, Borodulin K, Buman MP, Cardon G, et al. World Health Organization 2020 guidelines on physical activity and sedentary behaviour. British journal of sports medicine. 2020;54(24):1451-62.

22. Ekblom-Bak E, Borjesson M, Bergman F, Bergstrom G, Dahlin-Almevall A, Drake I, et al. Accelerometer derived physical activity patterns in 27.890 middle-aged adults: The SCAPIS cohort study. Scandinavian journal of medicine & science in sports. 2022;32(5):866-80.

23. Lewer D, Jayatunga W, Aldridge RW, Edge C, Marmot M, Story A, et al. Premature mortality attributable to socioeconomic inequality in England between 2003 and 2018: an observational study. Lancet Public Health. 2020;5(1):e33-e41.

24. Ekblom-Bak E, Vaisanen D, Ekblom B, Blom V, Kallings LV, Hemmingsson E, et al. Cardiorespiratory fitness and lifestyle on severe COVID-19 risk in 279,455 adults: a case

For peer review only

For peer review only - http://bmjopen.bmj.com/site/about/guidelines.xhtml

control study. The international journal of behavioral nutrition and physical activity. 2021;18(1):135.

25. Vaisanen D, Kallings LV, Andersson G, Wallin P, Hemmingsson E, Ekblom-Bak E. Lifestyle-associated health risk indicators across a wide range of occupational groups: a cross-sectional analysis in 72,855 workers. BMC public health. 2020;20(1):1656.

26. Korshoj M, Lund Rasmussen C, de Oliveira Sato T, Holtermann A, Hallman D. Heart rate during work and heart rate variability during the following night: a day-by-day investigation on the physical activity paradox among blue-collar workers. Scand J Work Environ Health. 2021;47(5):387-94.

27. Vaisanen D, Kallings LV, Andersson G, Wallin P, Hemmingsson E, Ekblom-Bak E. Cardiorespiratory Fitness in Occupational Groups-Trends over 20 Years and Future Forecasts. Int J Environ Res Public Health. 2021;18(16).

28. Holtermann A, Krause N, van der Beek AJ, Straker L. The physical activity paradox: six reasons why occupational physical activity (OPA) does not confer the cardiovascular health benefits that leisure time physical activity does. Br J Sports Med. 2018;52(3):149-50.

29. Schantz P, Salier Eriksson J, Rosdahl H. Perspectives on Exercise Intensity, Volume and Energy Expenditure in Habitual Cycle Commuting. Front Sports Act Living. 2020;2:65.

30. Stigell E, Schantz P. Active Commuting Behaviors in a Nordic Metropolitan Setting in Relation to Modality, Gender, and Health Recommendations. Int J Environ Res Public Health. 2015;12(12):15626-48.

For peer review only

For peer review only - http://bmjopen.bmj.com/site/about/guidelines.xhtml

FIGURE LEGENDS

Figure 1. Odds Ratios (95% Confidence intervals [CI]) for low CRF in association with correlates in the backward multiregression model. Odds Ratios are presented on log-scale. SED, sedentary; VPA, vigorous physical activity; perc, percent; Q, quartile.

Figure 2. Mean estimated VO_2max (95% Confidence Interval [CI]) in association with age-group (x-axis) and vigorous physical activity (VPA) level in min/week (left) and % of time in sedentary (right).

For peer review only

For peer review only - http://bmjopen.bmj.com/site/about/guidelines.xhtml

APPENDICE SUBMITTED

Additional File 1 – Questions used from SCAPIS study

Appendix Table 1 - Sex-specific estimated VO_2max (mL□min^{-1}□kg^{-1}) in association with all correlates presented in Table 1 to 3.

Appendix Figure 1 – Association between continuous levels of estimated VO_2max, BMI, and waist circumference.

Appendix Figure 2 - Association between estimated VO_2max and continuous levels of accelerometer assessed sedentary time and physical activity of different intensities.

For peer review only

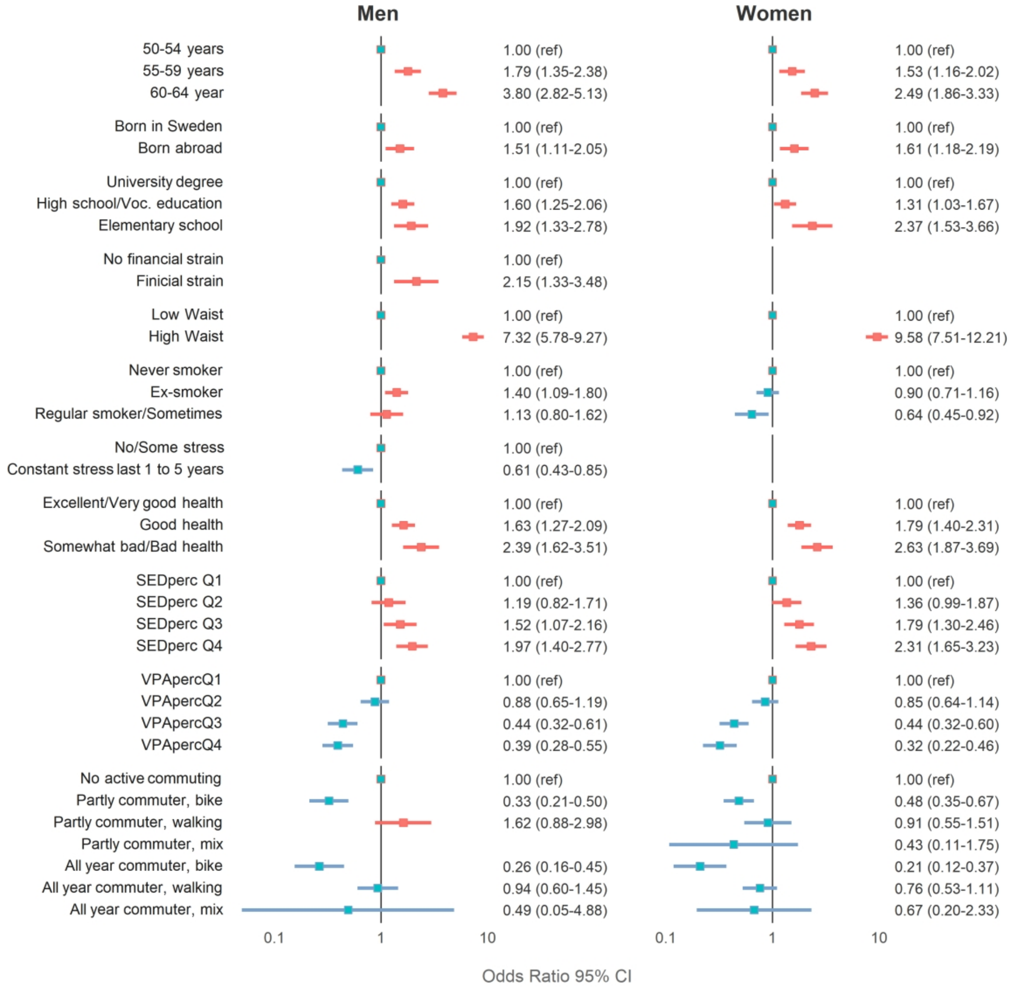

OR (95% CI) for low CRF in association with correlates in the backward multiregression model. OR are presented on log-scale. SED; sedentary, VPA; vigorous physical activity, perc; percent, Q; quartile.

517x517mm (157 x 157 DPI)

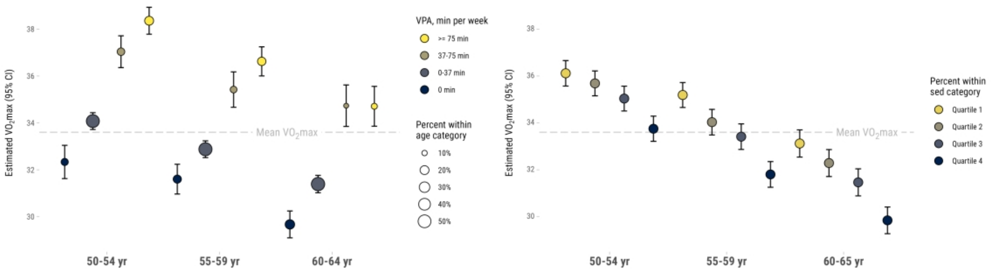

Mean estimated VO2max (95% CI) in association with age-group (x-axis) and VPA level in min/week (left) and % of time in sedentary (right).

1975x564mm (72 x 72 DPI)

Additional file 1. Questions from the SCAPIS questionnaire used in the present analyses. Reference to the original question is given if available.

Educational level

What is your highest completed level of education?

No formal education degree
Elementary school
High school / Vocational education
University degree

Employment status

What is your current occupation?

Employed, ______% of fulltime
On leave / parental leave
Studying
Labour market measure
Unemployed
Retired
Early or sick retiree

Financial strain

If you were to suddenly end up in a situation where you had to raise SEK 20,000 (≈EURO 2,000) in one week, would you be able to do it?

Yes
No

Strandhagen, E., et al. (2010). "Selection bias in a population survey with registry linkage: potential effect on socioeconomic gradient in cardiovascular risk." Eur J Epidemiol 25(3): 163-172.

Marital status

What is your current marital status?

Alone
Divorced
Married/Cohabitat
Widow

Born in Sweden

Are you born in Sweden?

Yes
No

Smoking habits

Do you smoke?

No, have never smoked
Yes, smokes regularly
No, stopped smoking
Yes, smokes occasionally

For peer review only

Commuting habits

Mode of travel to work:

Spring

Car
Bus/tram/train
Bicycling
Walking

Summer

Car
Bus/tram/train
Bicycling
Walking

Autumn

Car
Bus/tram/train
Bicycling
Walking

Winter

Car
Bus/tram/train
Bicycling
Walking

Wennberg, P., et al (2006). The effects of commuting activity and occupational and leisure time physical activity on risk of myocardial infarction. European Journal of Cardiovascular Prevention & Rehabilitation December 2006 13: 924-930

Sleep

How well are you sleeping usually?

Very well
Well
Rather well
Badly
Very badly

Adapted from the Basic Nordics Sleep Questionnaire Partinen, M. and T. Gislason (1995). "Basic Nordic Sleep Questionnaire (BNSQ): a quantitated measure of subjective sleep complaints." J Sleep Res 4(S1): 150-155.

Stress

By stress we mean feeling tense, irritable, anxious or having sleeping difficulties as a result of conditions at work or at home. Did you experience this?

Never experienced stress
$\geq$1 period of stress
$\geq$1 period of stress during the last 5 years
several periods of stress during the last 5 years
permanent stress during the last year or the last 5 years

For peer review only

Adapted from: Rosengren, A., et al. (2004). "Association of psychosocial risk factors with risk of acute myocardial infarction in 11119 cases and 13648 controls from 52 countries (the INTERHEART study): case-control study." Lancet 364(9438): 953-962.

Rosengren, A., et al. (1991). "Self-perceived psychological stress and incidence of coronary artery disease in middle-aged men." Am J Cardiol 68(11): 1171-1175.

Control at work

At work I feel that I have control over what happens in most situations

Strongly disagree
Disagree
Neutral
Agree
Strongly agree

Adapted from: Rosengren, A., et al. (2004). "Association of psychosocial risk factors with risk of acute myocardial infarction in 11119 cases and 13648 controls from 52 countries (the INTERHEART study): case-control study." Lancet 364(9438): 953-962.

Bobak, M., et al. (2000). "Socioeconomic factors, material inequalities, and perceived control in self-rated health: cross-sectional data from seven post-communist countries." Soc Sci Med 51(9): 1343-1350. + references therein.

Control in life

I feel that what happens in my life is often determined by factors beyond my control

Strongly disagree
Disagree
Neutral
Agree
Strongly agree

Adapted from: Rosengren, A., et al. (2004). "Association of psychosocial risk factors with risk of acute myocardial infarction in 11119 cases and 13648 controls from 52 countries (the INTERHEART study): case-control study." Lancet 364(9438): 953-962.

Bobak, M., et al. (2000). "Socioeconomic factors, material inequalities, and perceived control in self-rated health: cross-sectional data from seven post-communist countries." Soc Sci Med 51(9): 1343-1350. + references therein.

General health

In general, would you say your health is:

Excellent
Very good
Good
Fair
Poor

Adapted from the Swedish version of the SF-36 questionnaire.
Sullivan, M., et al. (1995). "The Swedish SF-36 Health Survey--I. Evaluation of data quality, scaling assumptions, reliability and construct validity across general populations in Sweden." Soc Sci Med 41(10): 1349-1358.

Sullivan, M. and J. Karlsson (1998). "The Swedish SF-36 Health Survey III. Evaluation of criterion-based validity: results from normative population." J Clin Epidemiol 51(11): 1105-1113.

For peer review only

Persson, L. O., et al. (1998). "The Swedish SF-36 Health Survey II. Evaluation of clinical validity: results from population studies of elderly and women in Gothenborg." J Clin Epidemiol 51(11): 1095-1103

Depression symptoms

During the past 4 weeks, have you had any of the following problems with your work or other regular daily activities as a result of any emotional problems (such as feeling depressed or anxious)?

Yes
No

Adapted from the Swedish version of the SF-36 questionnaire.

Sullivan, M., et al. (1995). "The Swedish SF-36 Health Survey--I. Evaluation of data quality, scaling assumptions, reliability and construct validity across general populations in Sweden." Soc Sci Med 41(10): 1349-1358.

Sullivan, M. and J. Karlsson (1998). "The Swedish SF-36 Health Survey III. Evaluation of criterion-based validity: results from normative population." J Clin Epidemiol 51(11): 1105-1113.

Persson, L. O., et al. (1998). "The Swedish SF-36 Health Survey II. Evaluation of clinical validity: results from population studies of elderly and women in Gothenborg." J Clin Epidemiol 51(11): 1095-1103

Prevalent chronic conditions

Which of these diseases has a doctor diagnosed you or have you been surgically treated for?

Myocardial infarction
Angina pectoris
Atrial fibrillation
Heart failure
Heart valve disease
CABG or PCI
Intervention of arterial stenosis other than coronary artery stenosis
Aortic intervention
Stroke
Hypertension
Dyslipidemia
Diabetes
Doctor-diagnosed COPD, chronic bronchitis or emphysema
Other lung disease
Rheumatic disease
Cancer

Physical working situation

Degree of physical activity at work

Physically heavy most of the time
Sometimes physically heavy
Light and mobile

For peer review only

Light but partly mobile
Sedentary or standing

Wareham, N. J., et al. (2003). "Validity and repeatability of a simple index derived from the short physical activity questionnaire used in the European Prospective Investigation into Cancer and Nutrition (EPIC) study." Public Health Nutr 6(4): 407-413.

InterAct, C., et al. (2012). "Validity of a short questionnaire to assess physical activity in 10 European countries." Eur J Epidemiol 27(1): 15-25.

Exercise habits

How often have you exercised or exercised in workout clothes in the last three months, in order to improve your fitness and/or to feel good?
Never
Occasionally - not regularly
1-2 times a week
2-3 times a week
More than 3 times per week

Wareham, N. J., et al. (2003). "Validity and repeatability of a simple index derived from the short physical activity questionnaire used in the European Prospective Investigation into Cancer and Nutrition (EPIC) study." Public Health Nutr 6(4): 407-413.

InterAct, C., et al. (2012). "Validity of a short questionnaire to assess physical activity in 10 European countries." Eur J Epidemiol 27(1): 15-25.

Total physical activity

How much physical movement and exertion have you had in the last 12 months?
Sedentary
Light exercise
Moderate exercise
Regular, vigorous exercise

Sweden PHao. The Swedish national public health survey. http://www.folkhalsomyndigheten.se/documents/statistik-uppfoljning/enkater-undersokningar/nationella-folkhalsoenkaten/frageformular/formular-nationella-folkhalsoenkaten-2012.pdf. 2012.

Leisure time sitting

The last question is about the time you spent sitting on weekdays during the last 7 days. Include time spent at work, at home, while doing course work and during leisure time. This may include time spent sitting at a desk, visiting friends, reading, or sitting or lying down to watch television. How much time did you spend sitting on a day?
____minutes

Adapted from the IPAQ questionnaire: Craig, C. L., et al. (2003). "International physical activity questionnaire: 12-country reliability and validity." Med Sci Sports Exerc 35(8): 1381-1395.

For peer review only

Appendix Table 1. Sex-specific estimated VO_2max (ml/min/kg) in association with all correlates presented in Table 1 to 3.

	Estimated VO_2max				Low estimated VO_2max		
	Men		Women		Men	Women	p-value for interaction
**Age**	n	Median (95% CI)	n	Median (95% CI)	OR[a] (95% CI)	OR (95% CI)	p=0.045
50-54 years	906	37.8 (37.3-38.3)	975	31.9 (31.4-32.3)	1 (ref)	1 (ref)	
55-59 years	885	36.3 (35.7-36.8)	927	30.5 (30.0-31.0)	1.49 (1.21-1.84)	1.44 (1.18-1.75)	
60-64 years	799	33.9 (33.6-34.7)	816	28.6 (28.1-29.1)	2.98 (2.42-3.66)	2.23 (1.83-2.73)	
**Civil status**							
Married/Cohabitation	1935	36.5 (36.0-36.8)	1831	30.7 (30.4-31.2)	1 (ref)	1 (ref)	p=0.655
Divorced/Single/Widower	610	35.3 (34.7-36.1)	851	29.7 (29.1-30.2)	1.43 (1.17-1.73)	1.34 (1.12-1.59)	
**Born in Sweden**							
Yes	2090	36.5 (36.1-36.8)	2251	30.7 (30.4-31.1)	1 (ref)	1 (ref)	p=0.633
No	472	34.9 (34.1-35.5)	451	28.9 (28.3-29.3)	1.85 (1.49-2.28)	1.68 (1.36-2.07)	
**Educational level**							
University degree	1087	37.5 (37.0-38.0)	1393	31.5 (31.2-32.0)	1 (ref)	1 (ref)	p=0.294
High school/Vocational education	1147	35.5 (35.1-35.9)	1068	29.5 (29.1-29.9)	1.86 (1.54-2.24)	1.83 (1.54-2.18)	
Elementary school	321	34.0 (33.3-34.8)	234	27.4 (26.5-28.6)	2.54 (1.95-3.32)	3.21 (2.41-4.28)	
**Employment**							
Working, ≥ 50% of full-time	2117	36.7 (36.5-37.0)	2225	31.0 (30.6-31.4)	1 (ref)	1 (ref)	p=0.574
Retired	123	33.3 (32.1-34.7)	108	27.6 (26.6-29.0)	1.48 (1.01-2.19)	1.60 (1.07-2.41)	
Disability pension/Sickness pension/Sick leave	115	33.7 (32.9-34.8)	184	26.7 (26.0-28.0)	2.16 (1.47-3.18)	2.91 (2.14-3.97)	
Unemployed/Student	191	33.6 (32.6-34.5)	174	29.3 (28.4-30.1)	2.27 (1.67-3.09)	1.53 (1.11-2.11)	

For peer review only

**Financial strain**							
No	2328	36.5 (36.1-36.8)	2431	30.7 (30.1-31.1)	1 (ref)	1 (ref)	p=0.293
Yes	177	33.0 (31.9-34.3)	229	28.0 (26.9-28.7)	2.91 (2.11-4.00)	2.24 (1.69-2.96)	
**Smoking habits**							
Never smoker	1287	37.3 (36.8-37.9)	1176	31.4 (31.0-31.8)	1 (ref)	1 (ref)	p=0.004
Ex-smoker	897	35.0 (34.6-35.5)	1118	29.9 (29.3-30.3)	1.65 (1.37-1.99)	1.17 (0.98-1.39)	
Regular smoker/Sometimes	341	35.1 (34.0-36.1)	375	29.9 (29.3-30.3)	2.02 (1.57-2.60	1.28 (0.99-1.64)	
**Pack-years**							
Never smokers (0)	1287	37.3 (36.8-37.9)	1176	31.4 (31.0-31.8)	1 (ref)	1 (ref)	p=0.008
Former smokers, low (<15 pack-years)	500	36.1 (35.4-36.7)	720	30.5 (30.1-31.1)	1.13 (0.90-1.43)	0.89 (0.73-1.09)	
Former smokers, heavy (≥15 pack-years)	397	33.4 (33.1-34.3)	398	28.1 (27.6-29.1)	2.57 (2.03-3.27)	1.82 (1.43-2.30)	
Current smokers, low (<15 pack-years)	106	36.5 (34.8-37.6)	120	31.6 (30.0-32.9)	1.57 (1.02-2.40)	0.79 (0.51-1.22)	
Current smokers, heavy (≥15 pack-years)	235	34.8 (33.6-35.6)	255	29.3 (28.3-29.9)	2.26 (1.69-3.02)	1.56 (1.18-2.07)	
**Alcohol abuse**							
No/Low	1881	36.6 (36.3-36.9)	2067	31.0 (30.5-31.3)	1 (ref)	1 (ref)	p=0.226
Moderate	355	35.4 (34.8-36.4)	284	30.8 (30.1-31.3)	1.43 (1.12-1.82)	1.08 (0.82-1.42)	
High	22	33.7 (31.1-35.0)	11	27.3 (24.6-33.3)	2.72 (1.13-6.50)	3.92 (1.18-13.04)	
**Sleep**							
Very well/well	1348	36.7 (36.2-37.0)	1053	30.8 (30.3-31.4)	1 (ref)	1 (ref)	p=0.253
Rather well/Badly/Very badly	1198	35.7 (35.4-36.1)	1637	30.2 (29.9-30.5)	1.31 (1.11-1.56)	1.14 (0.97-1.35)	
**Stress**							
No stress/Some stress during last 5 years	2140	36.3 (35.9-36.7)	1949	30.5 (30.3-31.0)	1 (ref)	1 (ref)	p=0.267
Constant stress last 1 to 5 years	400	35.6 (35.1-36.6)	729	29.9 (29.4-30.6)	1.14 (0.91-1.44)	1.32 (1.10-1.58)	

For peer review only

**At work I feel that that I have control in most situations**							
Strongly agree/Agree	1993	36.5 (36.1-36.8)	1853	30.8 (30.4-31.2)	1 (ref)	1 (ref)	p=0.730
Neutral/Do not agree/Do not agree at all	444	35.9 (35.1-36.6)	667	30.1 (29.5-30.9)	1.20 (0.96-1.50)	1.25 (1.04-1.51)	
**I feel that what happens in my life is often determined by factors beyond my control**							
Do not agree at all/Do not agree	1489	36.5 (36.1-36.9)	1487	31.3 (30.9-31.7)	1 (ref)	1 (ref)	p=0.004
Neutral/Agree/Strongly agree	1022	35.9 (35.4-36.4)	1171	29.3 (28.9-29.7)	1.22 (1.03-1.45)	1.72 (1.45-2.02)	
**General health**							
Excellent/Very good	1281	38.0 (37.5-38.3)	1417	32.1 (31.8-32.6)	1 (ref)	1 (ref)	p=0.116
Good	966	34.9 (34.5-35.4)	852	29.1 (28.6-29.7)	2.31 (1.91-2.79)	2.52 (2.08-3.04)	
Somewhat bad/Bad	300	33.3 (32.3-34.0)	429	26.4 (25.7-27.3)	4.13 (3.15-5.41)	5.40 (4.27-6.83)	
**BMI and estVO$_2$max in L·min^{-1}**							
<24.9	748	3.07 (3.03-3.12)	1288	2.16 (2.14-2.18)	1 (ref)	1 (ref)	p=0.001
25.0-29.9	1391	3.16 (3.13-3.19)	950	2.14 (2.12-2.17)	1.87 (1.46-2.40)	2.70 (2.11-3.45)	
30.0-34.9	379	3.21 (3.16-3.27)	349	2.18 (2.13-2.23)	4.15 (2.70-6.37)	7.36 (4.85-11.17)	
≥35	72	3.18 (3.06-3.30)	131	2.20 (2.10-2.25)	12.7 (5.7-28.3)	18.2 (9.63-34.3)	
**Waist circumference**							
Low waist (<88 cm women, <102 cm men)	1725	38.5 (38.2-38.9)	1466	34.0 (33.6-34.4)	1 (ref)	1 (ref)	p=0.041
High waist (≥88 cm women, ≥102cm men)	863	32.0 (31.6-32.5)	1251	26.7 (26.4-27.0)	9.15 (7.52-11.1)	11.5 (9.44-14.1)	
**Depression symptoms**							
No	2003	36.3 (35.9-36.7)	1684	30.8 (30.4-31.3)	1 (ref)	1 (ref)	p=0.230
Yes	519	36.0 (35.4-36.6)	976	29.9 (29.3-30.2)	1.21 (0.99-1.50)	1.41 (1.19-1.67)	
**Chronic conditions***							
0	1693	36.8 (36.4-37.1)	1772	31.1 (30.7-31.4)	1 (ref)	1 (ref)	p=0.186
1-2	816	35.1 (34.7-35.7)	889	29.3 (28.8-29.9)	1.30 (1.09-1.56)	1.46 (1.23-1.74)	

For peer review only

=>3	81	34.0 (32.7-35.3)	57	23.8 (22.6-29.1)	1.62 (1.02-2.56)	2.92 (1.70-5.03)	
**CVD, Hypertension and/or Lung disease**							
No	1980	36.6 (36.2-36.9)	2089	31.0 (30.5-31.3)	1 (ref)	1 (ref)	p=0.142
Yes	610	35.0 (34.6-35.6)	629	28.8 (28.3-29.4)	1.26 (1.04-1.53)	1.58 (1.31-1.90)	
**Commuting***							
No active commuting	1638	35.4 (35.1-35.8)	1419	29.9 (29.5-30.3)	1 (ref)	1 (ref)	p=0.831
Partly commuter, bike	270	38.6 (37.7-39.1)	401	32.1 (31.6-33.0)	0.35 (0.25-0.48)	0.45 (0.34-0.58)	
Partly commuter, walking	68	34.5 (32.7-35.8)	128	28.9 (28.1-29.8)	1.42 (0.86-2.33)	0.99 (0.68-1.44)	
Partly commuter, mix	3	44.2 (38.1-50.9)	16	29.8 (28.2-33.2)	-	0.57 (0.18-1.79)	
All year commuter, bike	203	41.3 (40.5-42.4)	204	35.0 (34.2-36.2)	0.23 (0.15-0.35)	0.14 (0.08-0.23)	
All year commuter, walking	162	36.9 (35.6-38.1)	253	30.3 (29.7-31.3)	0.79 (0.56-1.12)	0.66 (0.49-0.89)	
All year commuter, mix	8	40.2 (36.8-41.1)	22	32.3 (29.0-36.1)	0.26 (0.03-2.18)	0.61 (0.24-1.59)	
**Physical working situation**							
Sedentary to light	1894	36.7 (36.3-37.0)	2027	31.0 (30.5-31.4)	1 (ref)	1 (ref)	p=0.775
Sometimes to frequently heavy	498	35.3 (34.8-36.1)	447	29.8 (29.1-30.4)	1.48 (1.20-1.83)	1.40 (1.12-1.74)	
**Exercise habits**							
Never	722	33.7 (33.4-34.1)	662	27.4 (26.7-28.0)	1 (ref)	1 (ref)	p=0.333
Irregular	496	34.8 (34.0-35.3)	571	28.7 (28.2-29.3)	0.78 (0.62-0.99)	0.71 (0.57-0.90)	
1-2 times per week	456	36.7 (36.1-37.3)	576	30.7 (30.3-31.4)	0.35 (0.27-0.46)	0.34 (0.26-0.43)	
2-3 times per week	507	37.9 (37.2-38.5)	582	33.0 (32.3-33.6)	0.29 (0.22-0.38)	0.22 (0.17-0.28)	
>3 times per week	356	40.5 (39.7-41.0)	293	35.4 (34.6-36.2)	0.14 (0.10-0.20)	0.14 (0.10-0.21)	
**Total physical activity**							
Sedentary	315	32.4 (31.5-33.2)	225	25.5 (24.5-26.5)	1 (ref)	1 (ref)	p=0.021
Light	1098	34.9 (34.6-35.3)	1268	28.8 (28.4-29.1)	0.42 (0.32-0.55)	0.34 (0.25-0.46)	

For peer review only

Moderate	735	37.4 (37.0-38.2)	878	32.7 (32.2-33.2)	0.18 (0.14-0.25)	0.10 (0.07-0.14)	
Regular exercise/training	367	40.9 (40.1-41.5)	302	35.6 (35.0-36.2)	0.06 (0.04-0.10)	0.06 (0.04-0.09)	
**Leisure time sitting**							
Q1; <4 h per day	525	37.5 (37.0-38.1)	596	30.9 (30.1-31.7)	1 (ref)	1 (ref)	p=0.159
Q2; 4-6 h per day	499	36.2 (35.7-37.0)	540	30.8 (30.1-31.4)	1.29 (0.98-1.69)	1.19 (0.92-1.54)	
Q3; 6-8.5 h per day	438	36.9 (36.1-37.9)	487	31.3 (30.5-32.0)	1.06 (0.79-1.41)	1.16 (0.88-1.52)	
Q4; >8.5 h per day	527	36.3 (35.6-37.0)	431	30.3 (29.9-31.2)	1.18 (0.89-1.55)	1.46 (1.11-1.93)	
**% of time spent in Sedentary**							
Q1; <47%	462	37.5 (36.8-38.1)	805	31.9 (31.1-32.3)	1 (ref)	1 (ref)	p=0.033
Q2; 47%-54%	539	37.1 (36.6-37.7)	726	30.8 (30.2-31.4)	1.11 (0.83-1.48)	1.23 (0.97-1.54)	
Q3; 54%-61%	650	36.4 (35.8-37.0)	616	30.1 (29.5-30.7)	1.30 (0.99-1.71)	1.66 (1.31-2.09)	
Q4; >61%	803	34.8 (34.3-35.5)	464	27.9 (27.3-28.4)	1.97 (1.52-2.55)	3.01 (2.35-3.85)	
**% of time spent in prolonged Sedentary (of total wear time)**							
Q1; <13%	474	37.6 (37.0-38.2)	792	31.7 (30.8-32.2)	1 (ref)	1 (ref)	p=0.210
Q2; 13%-19%	536	37.2 (36.6-37.6)	731	31.1 (30.5-31.7)	1.24 (0.93-1.66)	1.08 (0.86-1.35)	
Q3; 19%-26%	673	36.1 (35.5-36.7)	592	30.1 (29.5-30.5)	1.55 (1.18-2.03)	1.64 (1.30-2.07)	
Q4; >26%	771	34.9 (34.4-35.5)	496	28.1 (27.7-28.6)	2.00 (1.54-2.60)	2.42 (1.90-3.08)	
**% of time spent in Light physical activity**							
Q1; <33%	840	35.4 (34.8-35.8)	427	28.6 (27.9-29.7)	1 (ref)	1 (ref)	p=0.124
Q2; 33%-40%	629	36.1 (35.5-36.7)	637	30.3 (29.8-31.0)	0.87 (0.70-1.09)	0.66 (0.51-0.85)	
Q3; 40%-46%	542	37.1 (36.7-37.8)	723	30.6 (30.0-31.2)	0.64 (0.50-0.81)	0.53 (0.41-0.69)	
Q4; >46%	443	37.0 (36.4-37.6)	824	31.0 (30.5-31.8)	0.67 (0.52-0.86)	0.46 (0.36-0.59)	
**% of time spent in Moderate physical activity**							
Q1; <3.8%	655	34.7 (34.1-35.3)	612	28.3 (27.7-28.9)	1 (ref)	1 (ref)	p=0.062

For peer review only

Q2; 3.8%-5.3%	581	36.4 (35.7-37.0)	684	30.1 (29.7-30.7)	0.61 (0.48-0.78)	0.61 (0.49-0.77)	
Q3; 5.3%-7.2%	594	37.0 (36.2-37.7)	672	31.0 (30.3-31.5)	0.56 (0.44-0.71)	0.48 (0.38-0.61)	
Q4; >7.2%	624	37.2 (36.7-37.9)	643	32.1 (31.3-32.5)	0.51 (0.40-0.65)	0.38 (0.30-0.48)	
**% of time spent in Vigorous physical activity**							
Q1;<0.02%	557	33.7 (33.3-34.2)	709	28.0 (27.4-28.5)	1 (ref)	1 (ref)	p=0.060
Q2; 0.02%-0.1%	566	34.9 (34.1-35.6)	700	28.7 (28.2-29.4)	0.79 (0.62-1.00)	0.82 (0.67-1.02)	
Q3; 0.1%-0.6%	640	37.1 (36.6-37.6)	627	31.4 (31.0-31.9)	0.37 (0.29-0.47)	0.31 (0.25-0.40)	
Q4; >0.6%	691	38.7 (38.1-39.4)	575	34.7 (33.9-35.3)	0.26 (0.20-0.33)	0.17 (0.13-0.23)	

[a] Adjusted for sex and age, comparing the lowest sex-specific tertile of estimated VO_2max with the two higher tertiles

[#] Sex-specific tertiles based on L/min. Analyses additionally adjusted for weight in kg.

For peer review only

Appendix Figure 1. Association between continous levels of estimated VO2max, BMI and waist circumference.

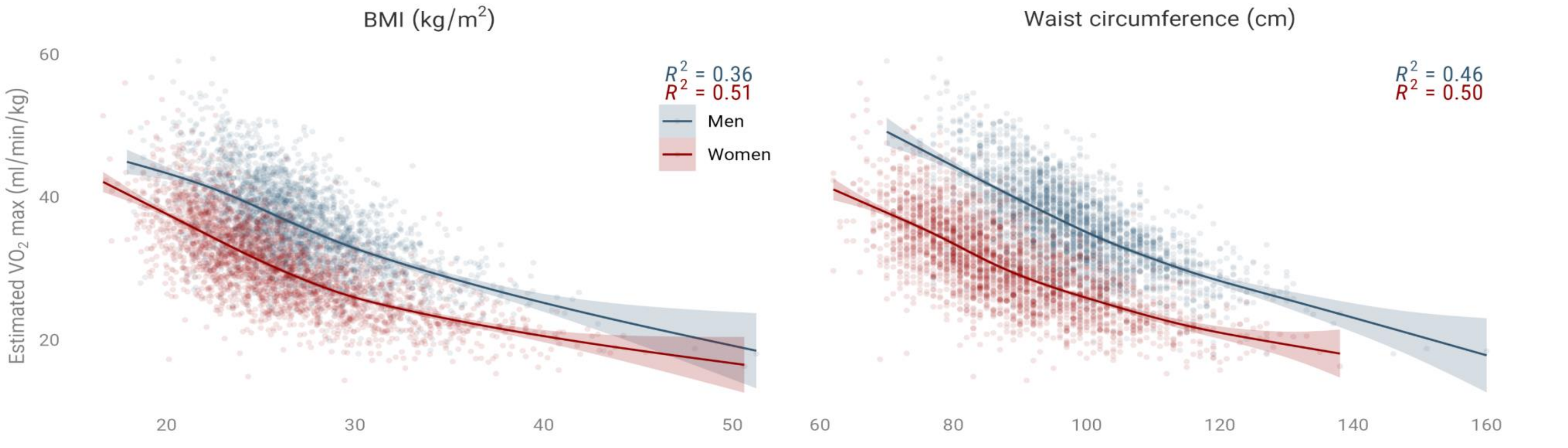

Appendix Figure 2. Association between estimated VO2max and continuous levels of accelerometer assessed sedentary time and physical activity of different intensities.

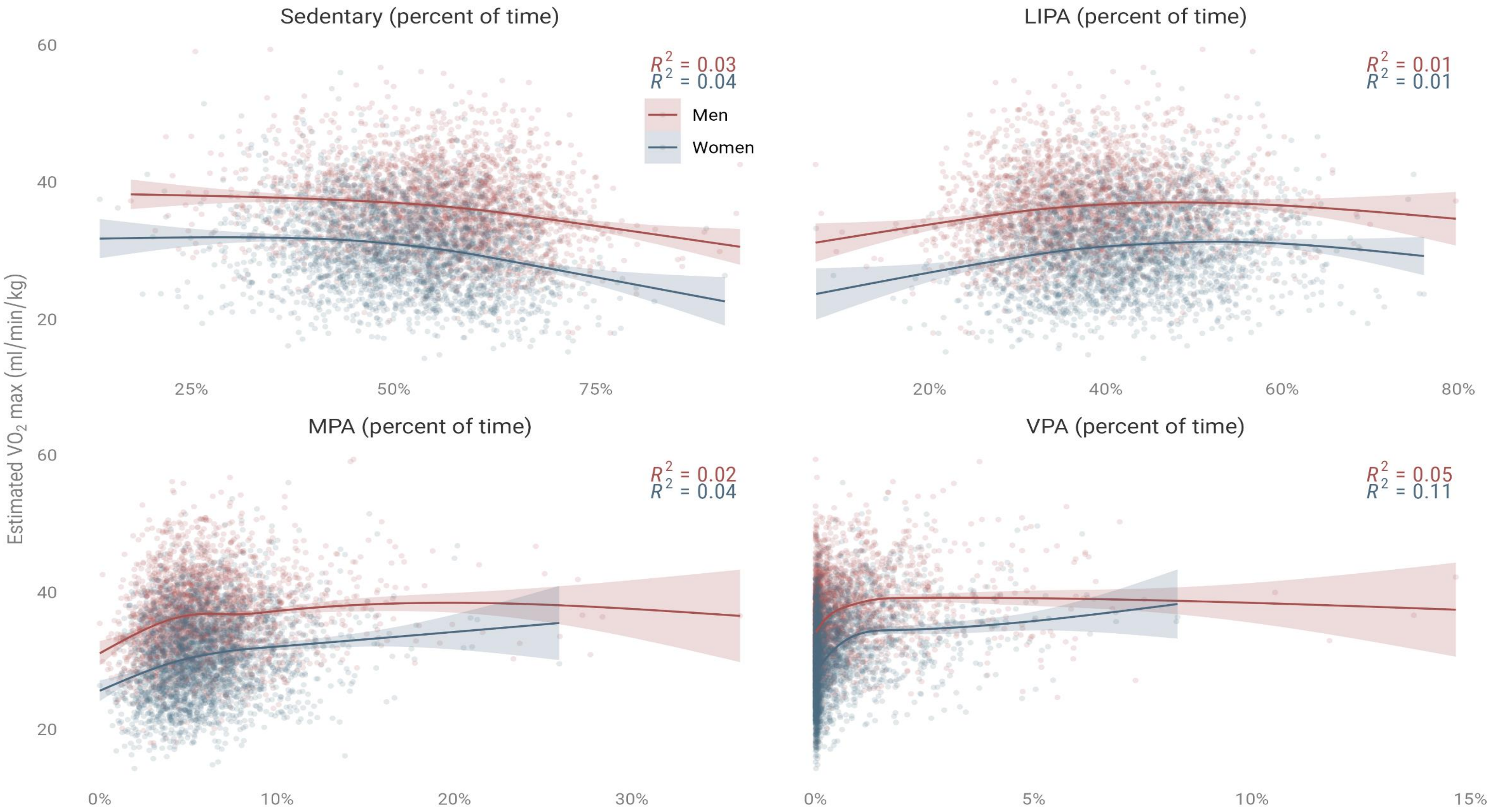

STROBE Statement—checklist of items that should be included in reports of observational studies

	Item No.	Recommendation	Page No.	Relevant text from manuscript
**Title and abstract**	1	(*a*) Indicate the study's design with a commonly used term in the title or the abstract	2	
		(*b*) Provide in the abstract an informative and balanced summary of what was done and what was found	2	
**Introduction**				
Background/rationale	2	Explain the scientific background and rationale for the investigation being reported	4	
Objectives	3	State specific objectives, including any prespecified hypotheses	4-5	
**Methods**				
Study design	4	Present key elements of study design early in the paper	5	
Setting	5	Describe the setting, locations, and relevant dates, including periods of recruitment, exposure, follow-up, and data collection	5	
Participants	6	*Cohort study*—Give the eligibility criteria, and the sources and methods of selection of participants. Describe methods of follow-up<br>***Case-control study*—Give the eligibility criteria, and the sources and methods of case ascertainment and control selection. Give the rationale for the choice of cases and controls**<br>*Cross-sectional study*—Give the eligibility criteria, and the sources and methods of selection of participants	5	
		(*b*) *Cohort study*—For matched studies, give matching criteria and number of exposed and unexposed<br>*Case-control study*—For matched studies, give matching criteria and the number of controls per case		
Variables	7	Clearly define all outcomes, exposures, predictors, potential confounders, and effect modifiers. Give diagnostic criteria, if applicable	5-7	
Data sources/ measurement	8*	For each variable of interest, give sources of data and details of methods of assessment (measurement). Describe comparability of assessment methods if there is more than one group	5-7	
Bias	9	Describe any efforts to address potential sources of bias	7-8	
Study size	10	Explain how the study size was arrived at	8	

Continued on next page

For peer review only - http://bmjopen.bmj.com/site/about/guidelines.xhtml
For peer review only

Quantitative variables	11	Explain how quantitative variables were handled in the analyses. If applicable, describe which groupings were chosen and why	7-8
Statistical methods	12	(*a*) Describe all statistical methods, including those used to control for confounding	7-8
		(*b*) Describe any methods used to examine subgroups and interactions	7-8
		€ Explain how missing data were addressed	
		(*d*) *Cohort study*—If applicable, explain how loss to follow-up was addressed<br>***Case-control study*—If applicable, explain how matching of cases and controls was addressed**<br>*Cross-sectional study*—If applicable, describe analytical methods taking account of sampling strategy	7-8
		€ Describe any sensitivity analyses	
**Results**			
Participants	13*	(a) Report numbers of individuals at each stage of study—eg numbers potentially eligible, examined for eligibility, confirmed eligible, included in the study, completing follow-up, and analysed	8
		(b) Give reasons for non-participation at each stage	8
		© Consider use of a flow diagram	
Descriptive data	14*	(a) Give characteristics of study participants (eg demographic, clinical, social) and information on exposures and potential confounders	8-9
		(b) Indicate number of participants with missing data for each variable of interest	8-9
		(c) *Cohort study*—Summarise follow-up time (eg, average and total amount)	
Outcome data	15*	*Cohort study*—Report numbers of outcome events or summary measures over time	
		Case-control study—Report numbers in each exposure category, or summary measures of exposure	
		Cross-sectional study—Report numbers of outcome events or summary measures	
Main results	16	(*a*) Give unadjusted estimates and, if applicable, confounder-adjusted estimates and their precision (eg, 95% confidence interval). Make clear which confounders were adjusted for and why they were included	8-11
		(*b*) Report category boundaries when continuous variables were categorized	8-11
		(*c*) If relevant, consider translating estimates of relative risk into absolute risk for a meaningful time period	

Continued on next page

For peer review only - http://bmjopen.bmj.com/site/about/guidelines.xhtml

Other analyses	17	Report other analyses done—eg analyses of subgroups and interactions, and sensitivity analyses	8-11
**Discussion**			
Key results	18	Summarise key results with reference to study objectives	11-14
Limitations	19	Discuss limitations of the study, taking into account sources of potential bias or imprecision. Discuss both direction and magnitude of any potential bias	14
Interpretation	20	Give a cautious overall interpretation of results considering objectives, limitations, multiplicity of analyses, results from similar studies, and other relevant evidence	11-14
Generalisability	21	Discuss the generalizability (external validity) of the study results	15
**Other information**			
Funding	22	Give the source of funding and the role of the funders for the present study and, if applicable, for the original study on which the present article is based	16

*Give information separately for cases and controls in case-control studies and, if applicable, for exposed and unexposed groups in cohort and cross-sectional studies.

Note: An Explanation and Elaboration article discusses each checklist item and gives methodological background and published examples of transparent reporting. The STROBE checklist is best used in conjunction with this article (freely available on the Web sites of PLoS Medicine at http://www.plosmedicine.org/, Annals of Internal Medicine at http://www.annals.org/, and Epidemiology at http://www.epidem.com/). Information on the STROBE Initiative is available at www.strobe-statement.org.

For peer review only
